# Supplementary material for: Mitochondrial protein carboxyl-terminal alanine-threonine tailing promotes human glioblastoma growth by regulating mitochondrial function
Source: eLife. 2026 Jan 29;13:RP99438. doi: 10.7554/eLife.99438 (PMC12854676; doi:10.7554/eLife.99438)
Supplement: Figure 1—source data 1. [file elife-99438-fig1-data1.zip › Figure 1-source data 1.pdf]

Figure 1B

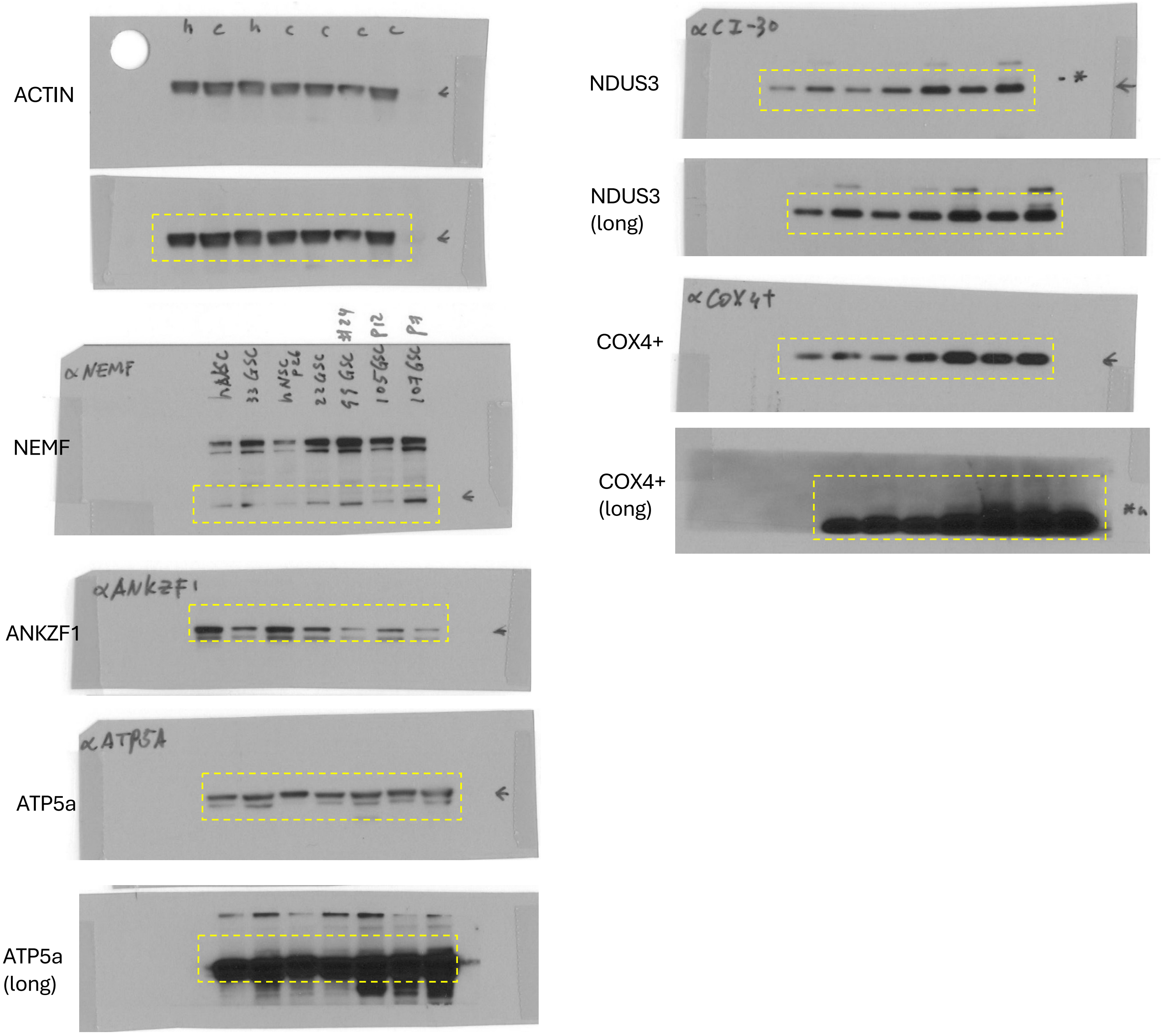

Figure 1, source data 1
Original membranes corresponding to Figure 1B. The sample labels are indicated above the NEMF blot.

Figure 1C

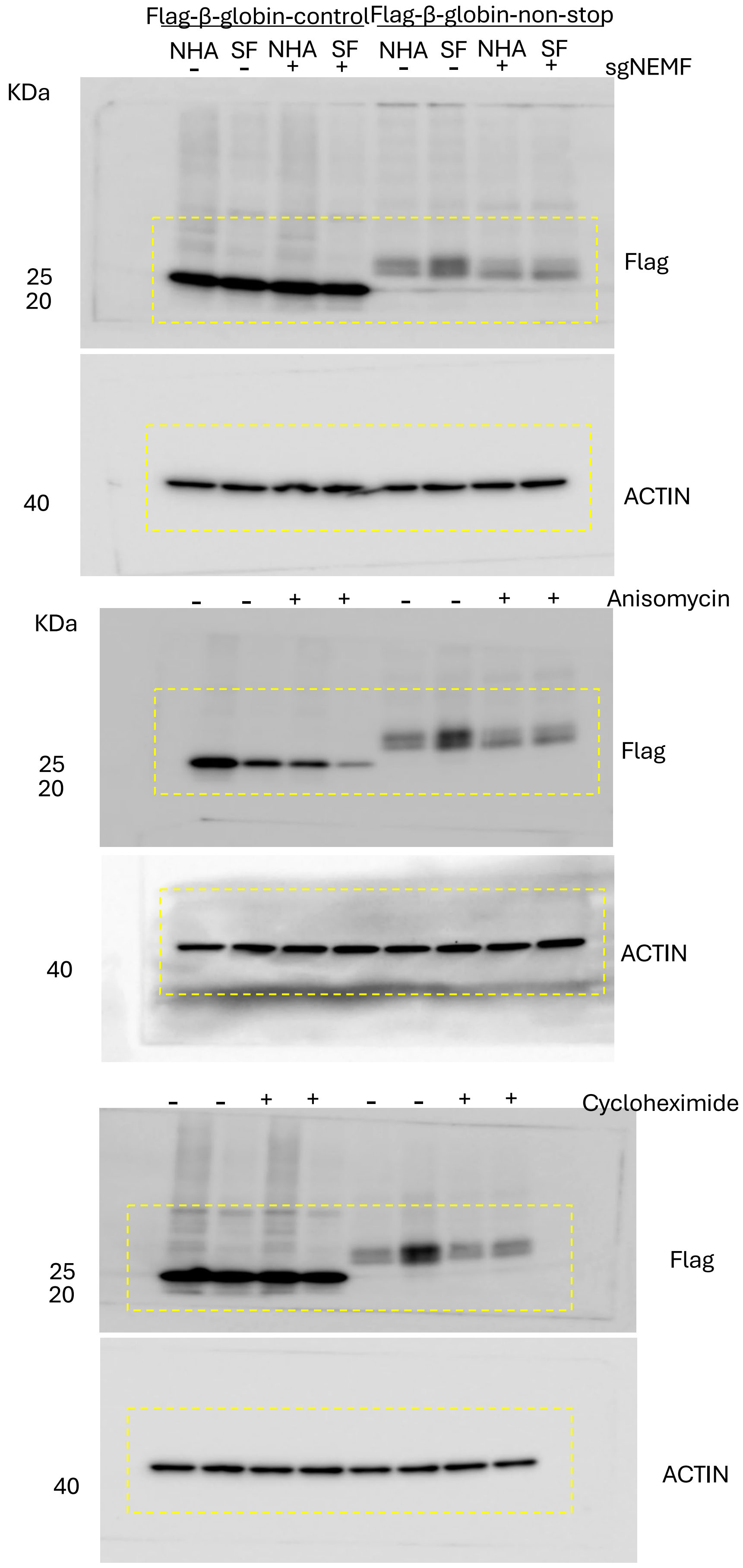

Figure 1, source data 1
Original membranes corresponding to Figure 1C.

Figure 1D

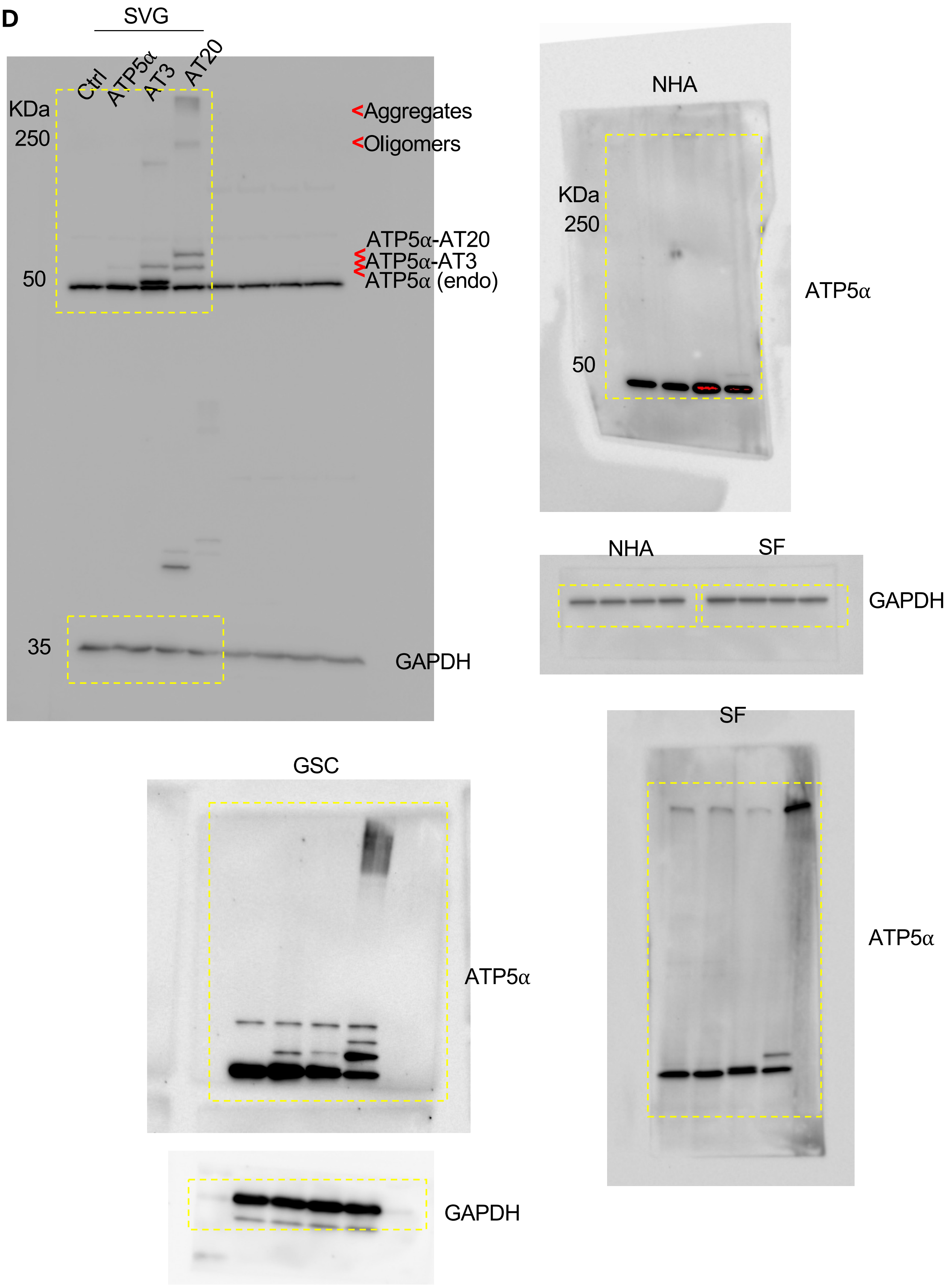

Figure 1, source data 1
Original membranes corresponding to Figure 1D.
