# Supplementary figures and images for: Mitochondrial protein carboxyl-terminal alanine-threonine tailing promotes human glioblastoma growth by regulating mitochondrial function
Source: eLife. 2026 Jan 29;13:RP99438. doi: 10.7554/eLife.99438 (PMC12854676; doi:10.7554/eLife.99438)

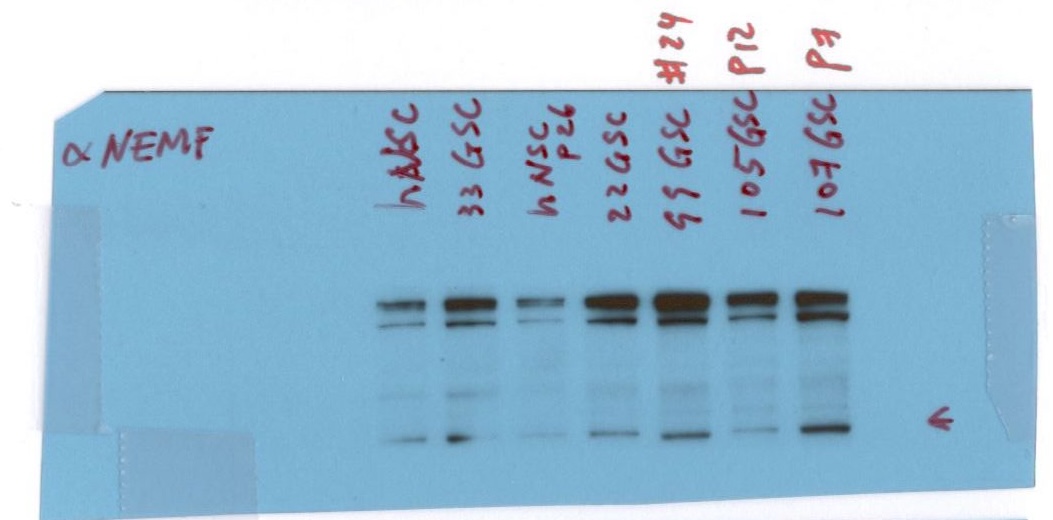

Supplement: Figure 1—source data 2. [file elife-99438-fig1-data2.zip › Figure 1-source data 2/Figure 1B NEMF.jpeg]

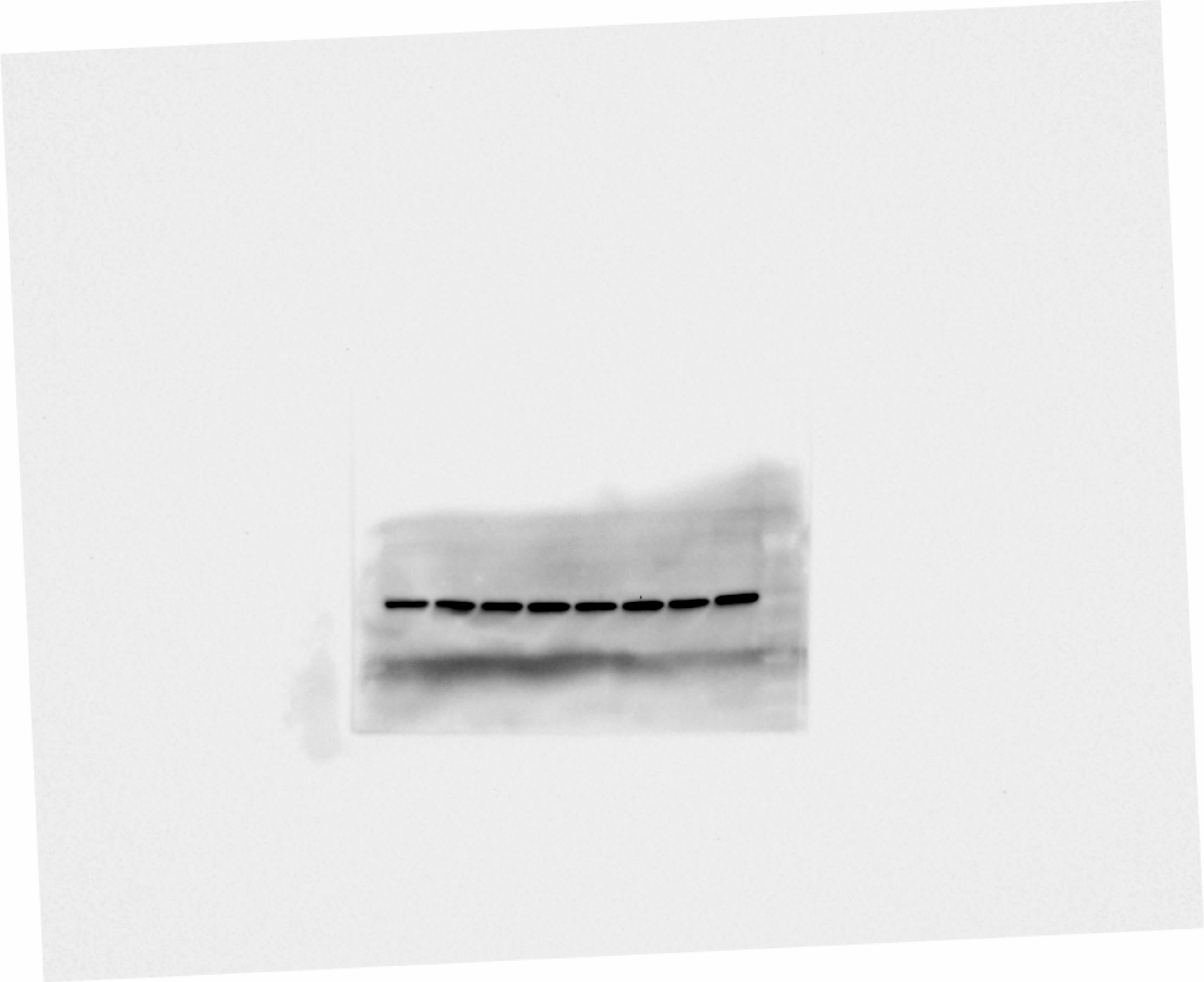

Supplement: Figure 1—source data 2. [file elife-99438-fig1-data2.zip › Figure 1-source data 2/Figure 1C anisomycin anti actin; 2024-05-03 11h01m37s.tif]

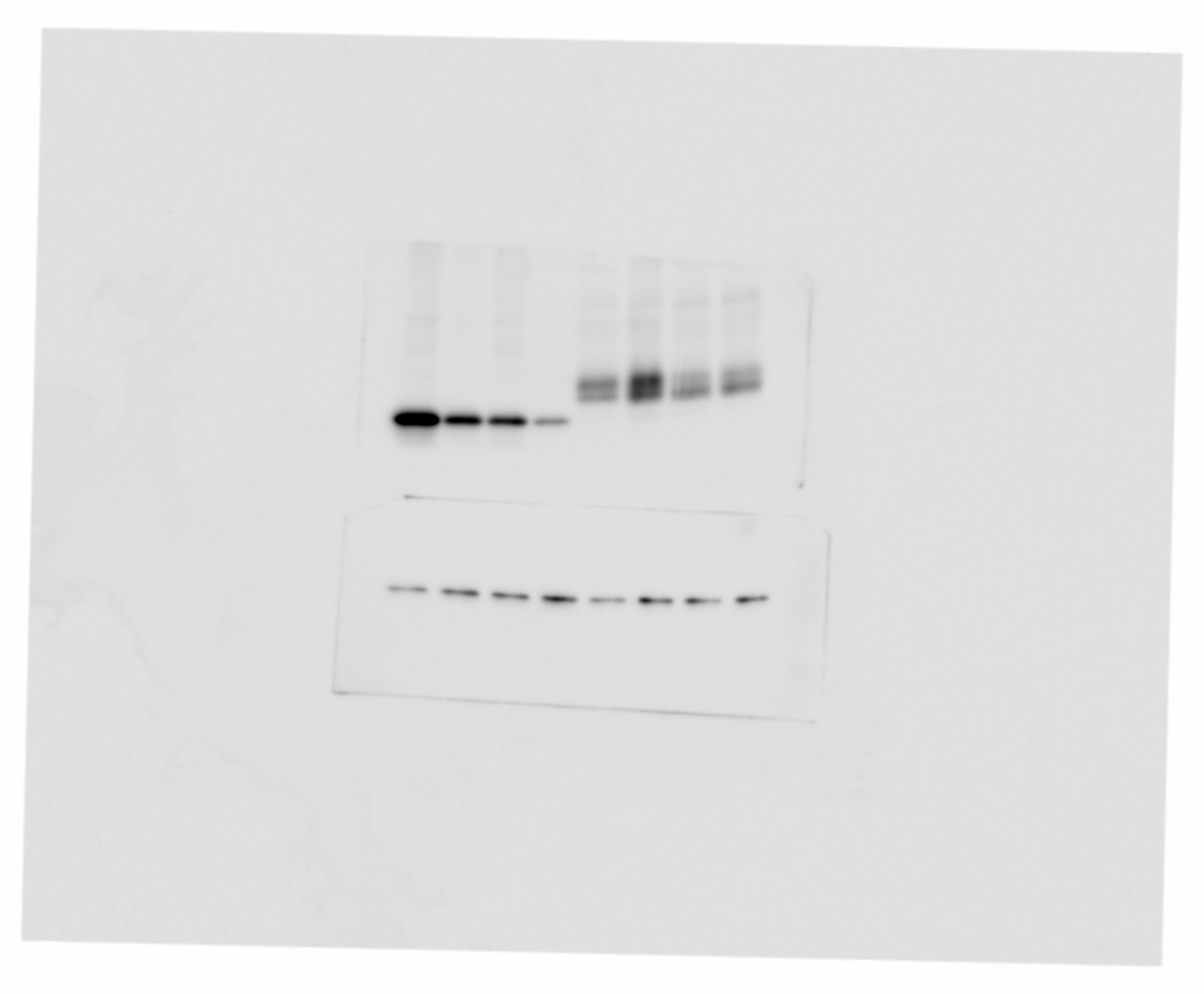

Supplement: Figure 1—source data 2. [file elife-99438-fig1-data2.zip › Figure 1-source data 2/Figure 1C anisomycin anti flag; 2025-05-15 13h09m25s.tif]

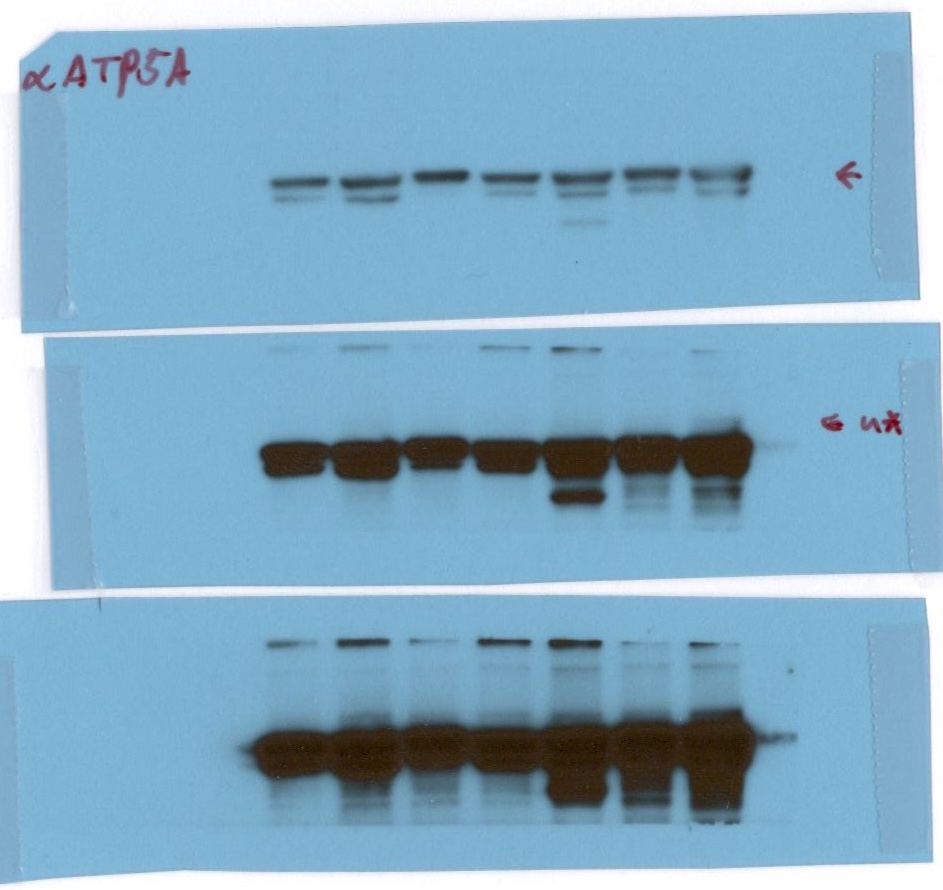

Supplement: Figure 1—source data 2. [file elife-99438-fig1-data2.zip › Figure 1-source data 2/Figure 1B ATP5a.jpeg]

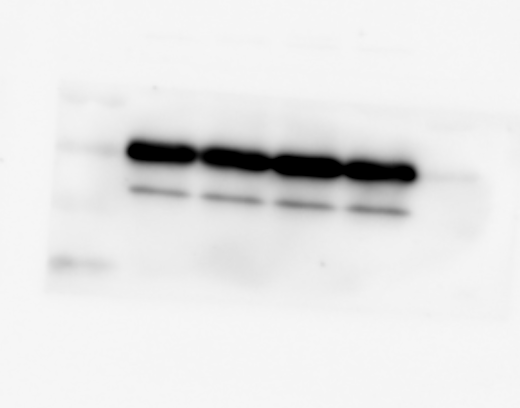

Supplement: Figure 1—source data 2. [file elife-99438-fig1-data2.zip › Figure 1-source data 2/Figure 1D GSC anti GAPDH; Ting 2023-02-16 16h15m15s.tif]

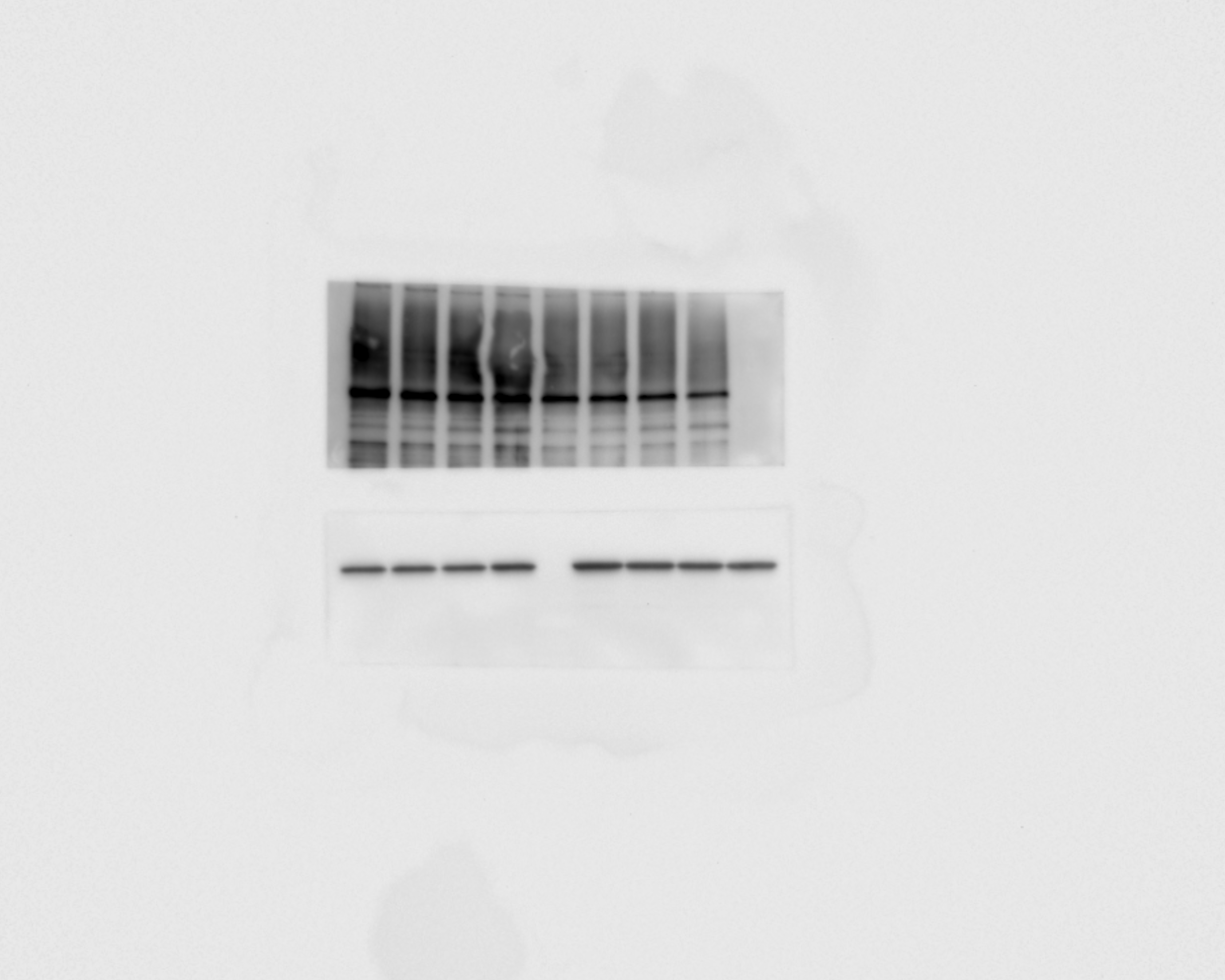

Supplement: Figure 1—source data 2. [file elife-99438-fig1-data2.zip › Figure 1-source data 2/Figure 1D NHA anti gapdh; SF anti gapdh; 2024-09-19 11h50m16s.tif]

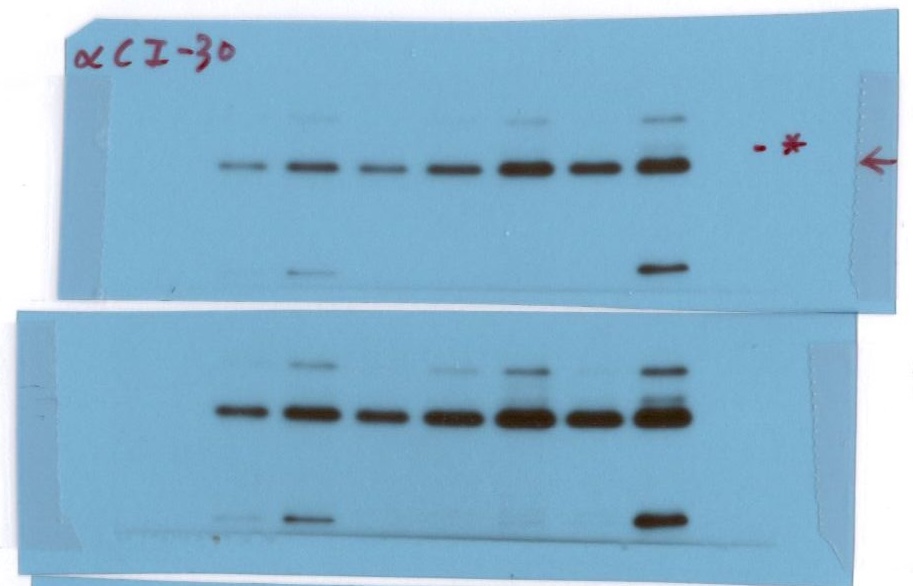

Supplement: Figure 1—source data 2. [file elife-99438-fig1-data2.zip › Figure 1-source data 2/Figure 1B NDUS3.jpeg]

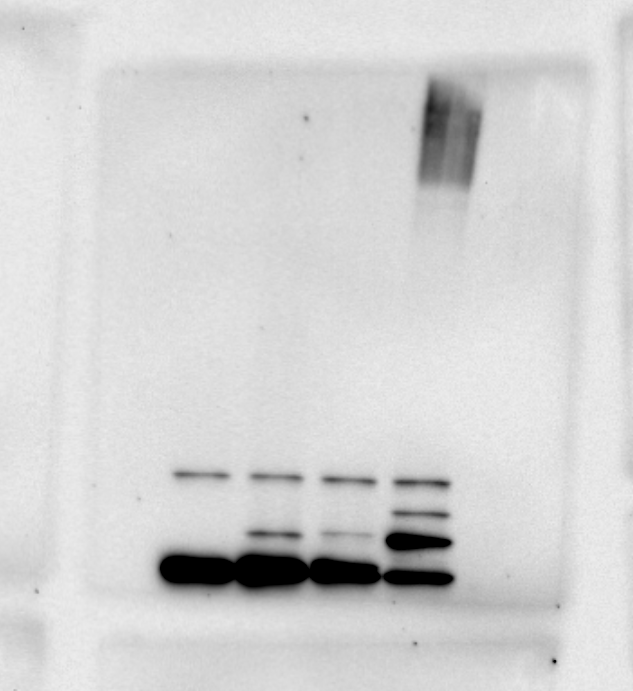

Supplement: Figure 1—source data 2. [file elife-99438-fig1-data2.zip › Figure 1-source data 2/Figure 1D GSC anti atp5a; Ting 2023-02-16 16h24m26s.tif]

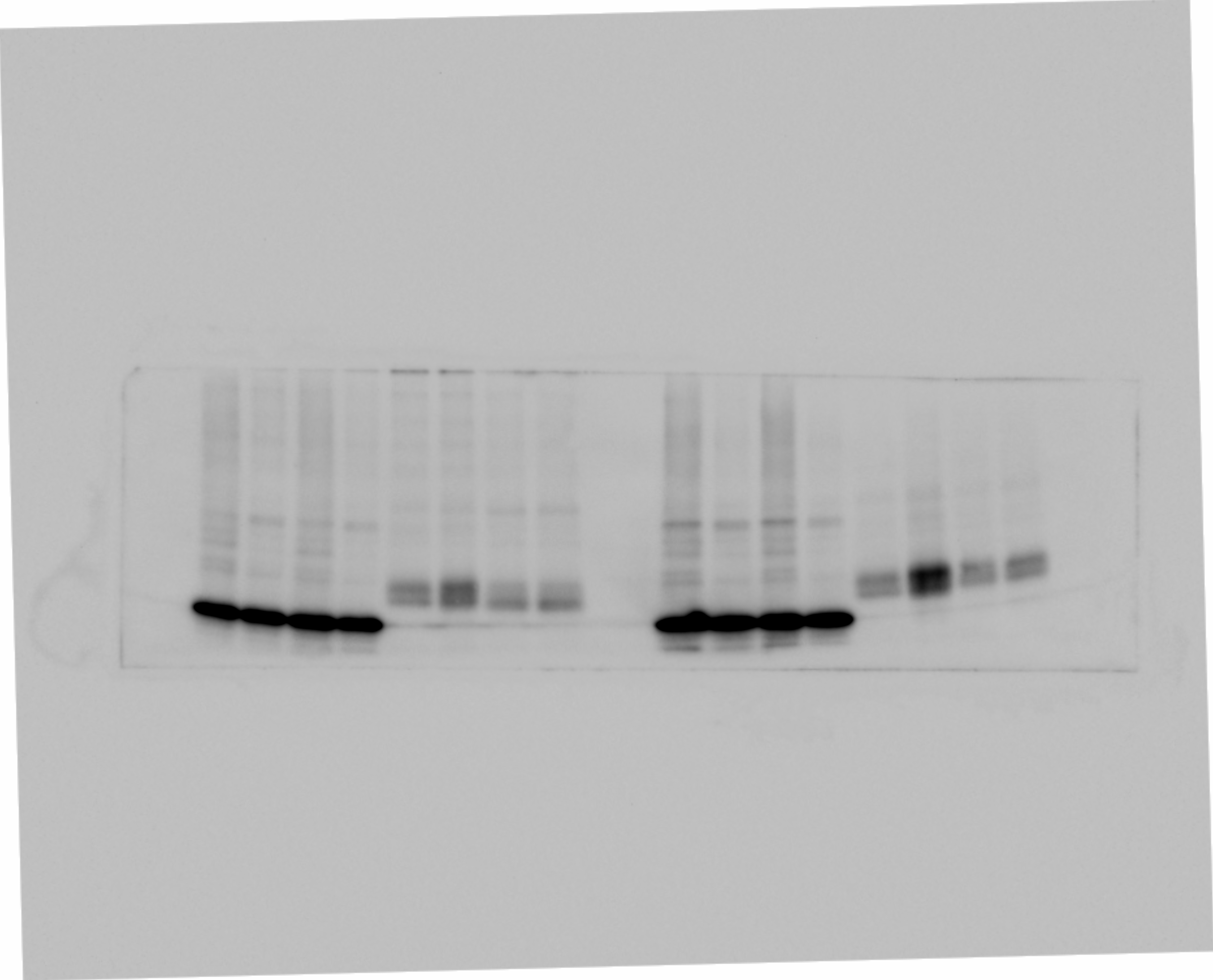

Supplement: Figure 1—source data 2. [file elife-99438-fig1-data2.zip › Figure 1-source data 2/Figure 1C sgNEMF anti flag; Cycloheximide anti flag; 2025-03-10 13h02m36s.tif]

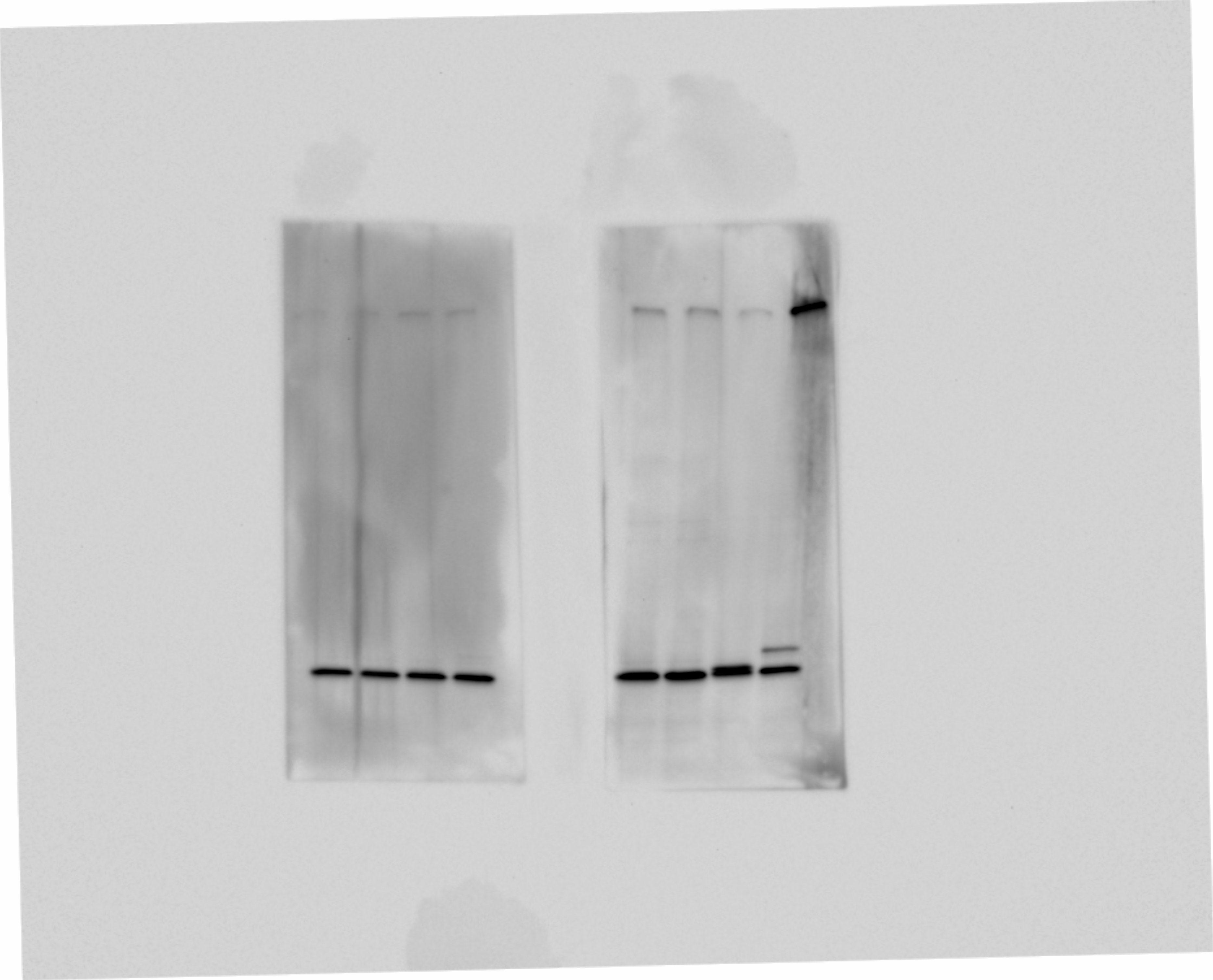

Supplement: Figure 1—source data 2. [file elife-99438-fig1-data2.zip › Figure 1-source data 2/Figure 1D SF anti atp5a; 2024-09-19 11h31m26s.tif]

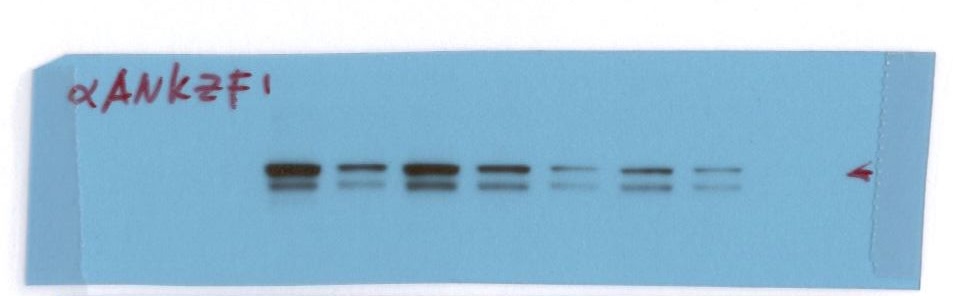

Supplement: Figure 1—source data 2. [file elife-99438-fig1-data2.zip › Figure 1-source data 2/Figure 1B ANKZF1.jpeg]

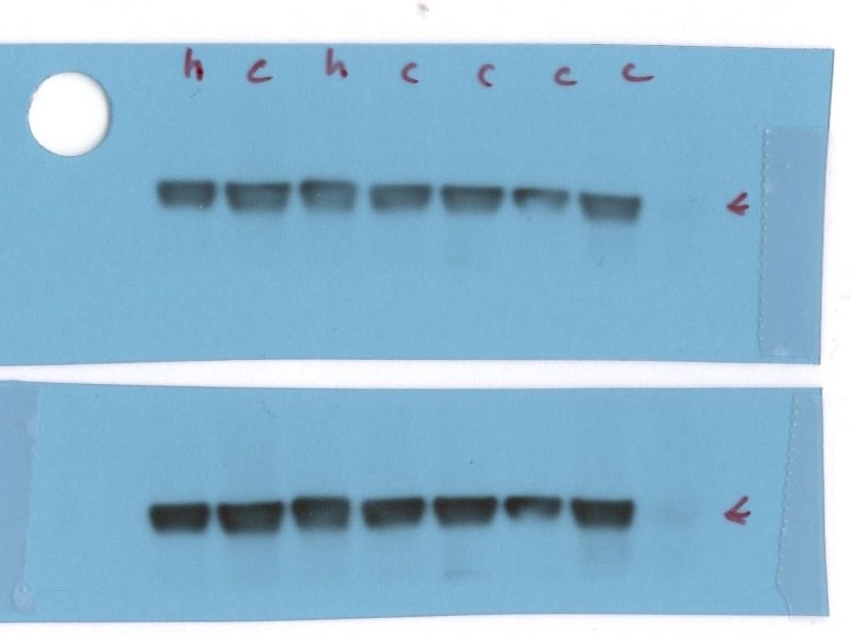

Supplement: Figure 1—source data 2. [file elife-99438-fig1-data2.zip › Figure 1-source data 2/Figure 1B ACTIN.jpeg]

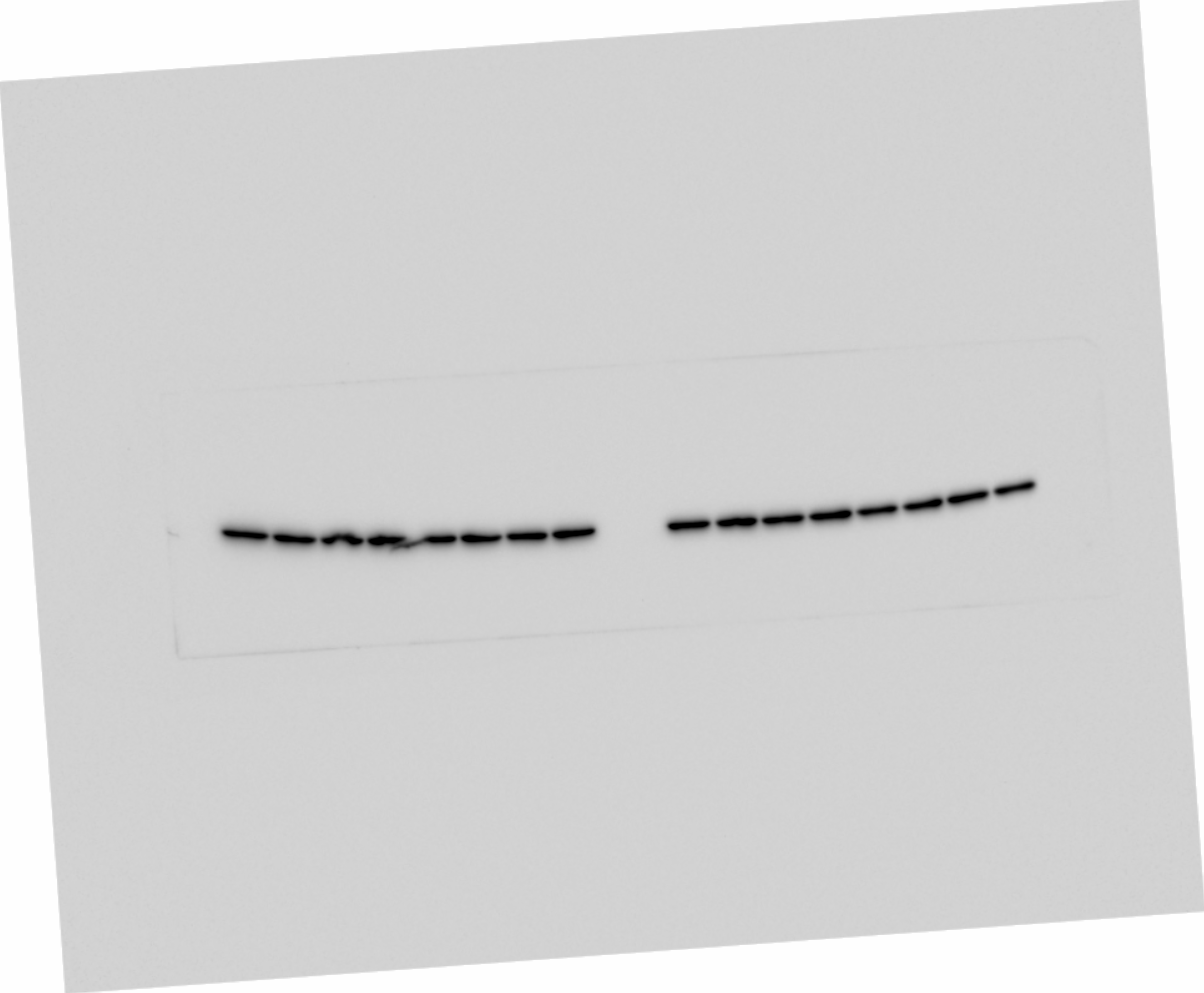

Supplement: Figure 1—source data 2. [file elife-99438-fig1-data2.zip › Figure 1-source data 2/Figure 1C sgNEMF anti actin; cycloheximide anti actin; 2025-03-10 12h57m45s.tif]

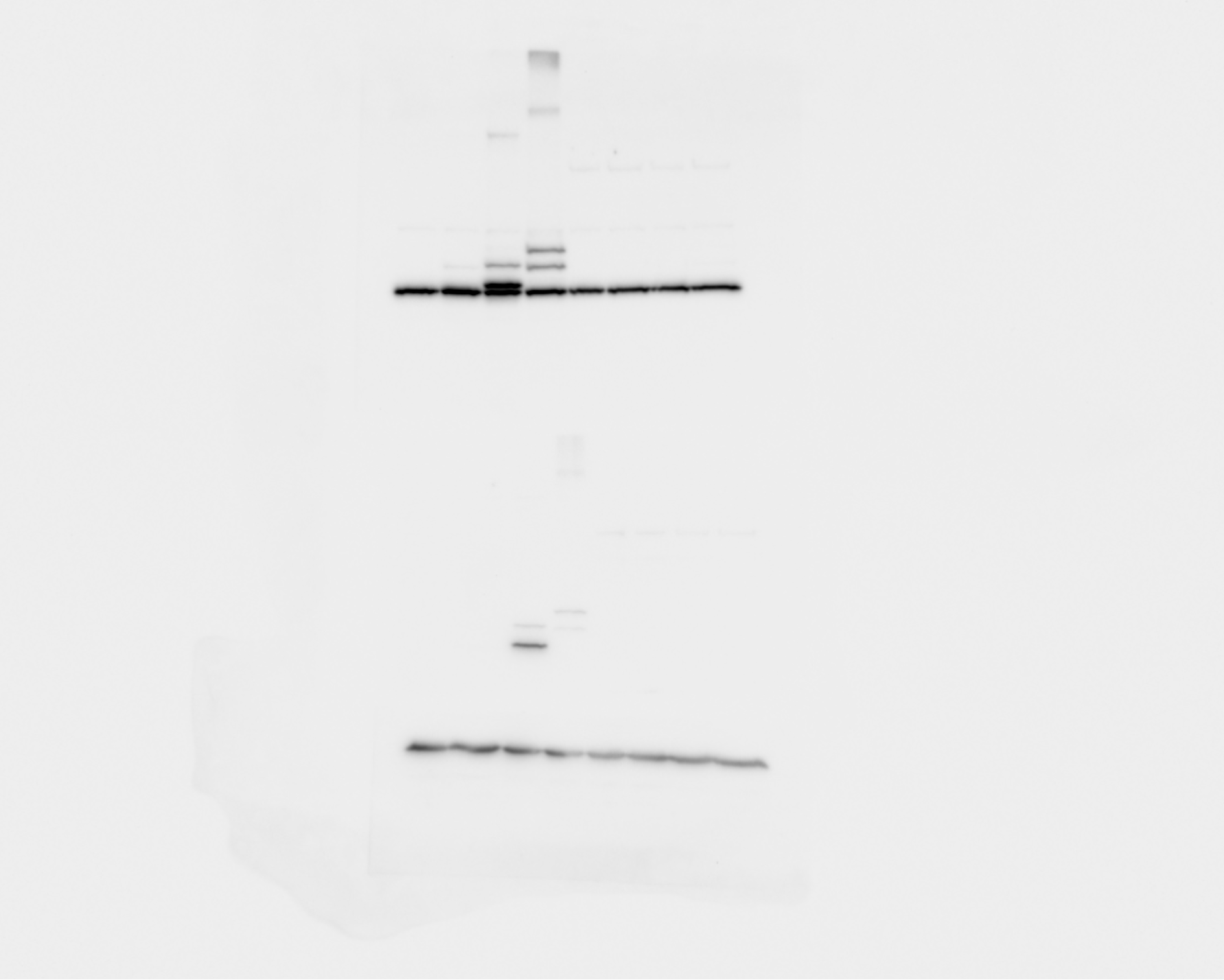

Supplement: Figure 1—source data 2. [file elife-99438-fig1-data2.zip › Figure 1-source data 2/Figure 1D SVG anti atp5a, GAPDH; Ting 2021-12-16 11h46m29s.tif]

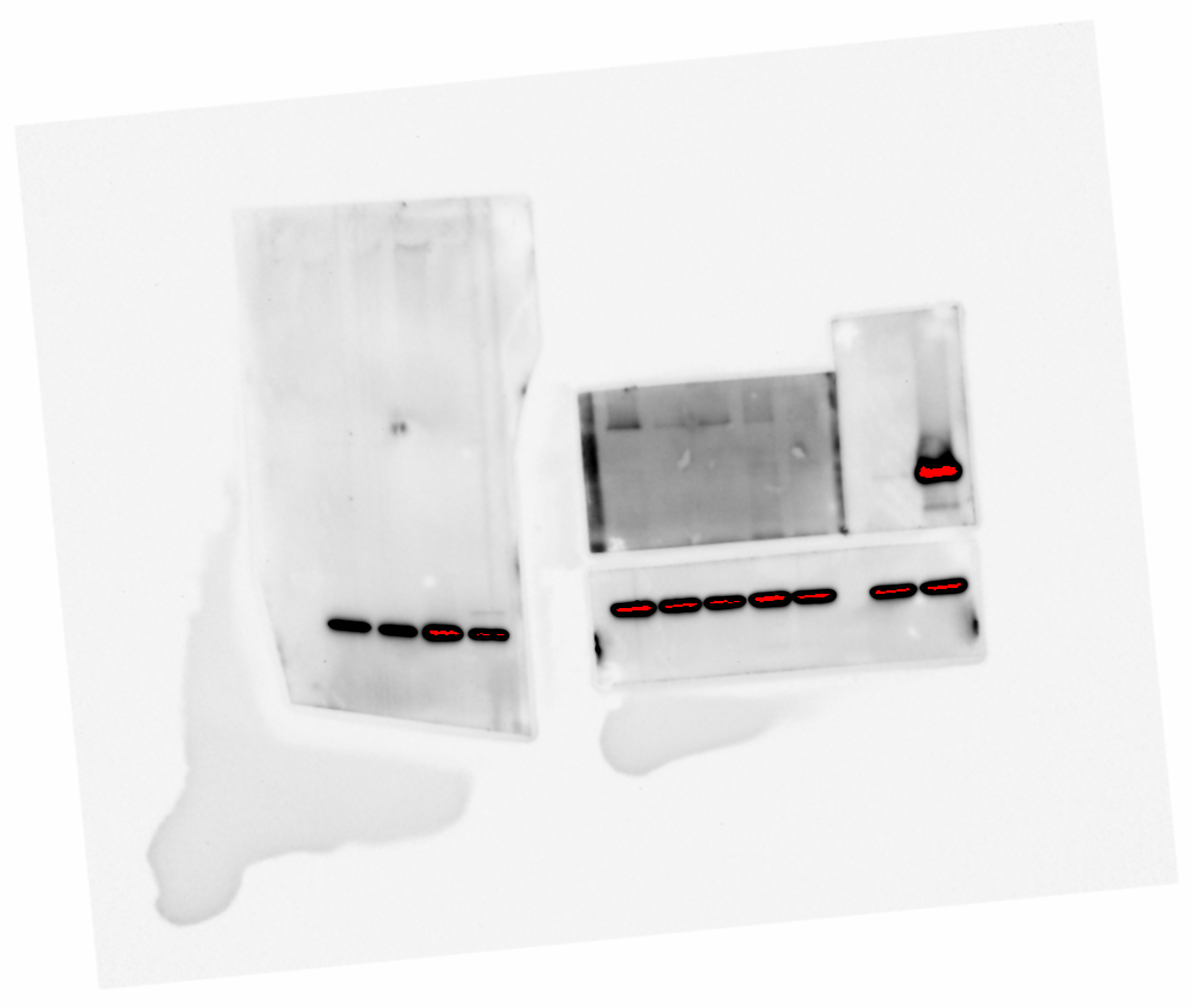

Supplement: Figure 1—source data 2. [file elife-99438-fig1-data2.zip › Figure 1-source data 2/Figure 1D NHA anti atp5a; 2025-05-15 13h04m43s.tif]

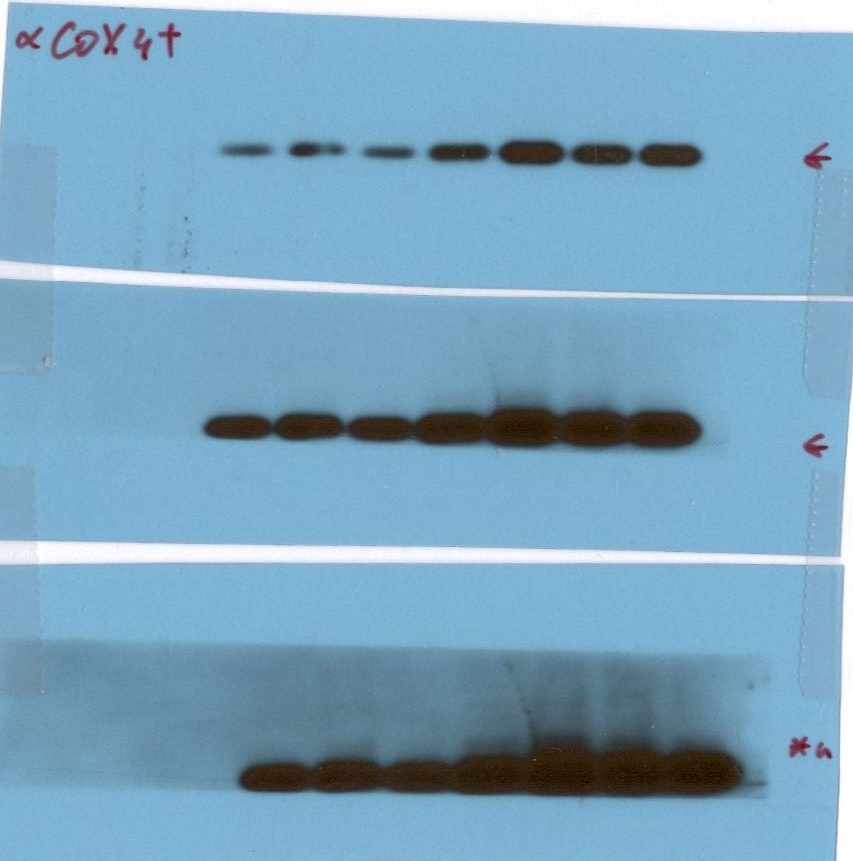

Supplement: Figure 1—source data 2. [file elife-99438-fig1-data2.zip › Figure 1-source data 2/Figure 1B COX4.jpeg]

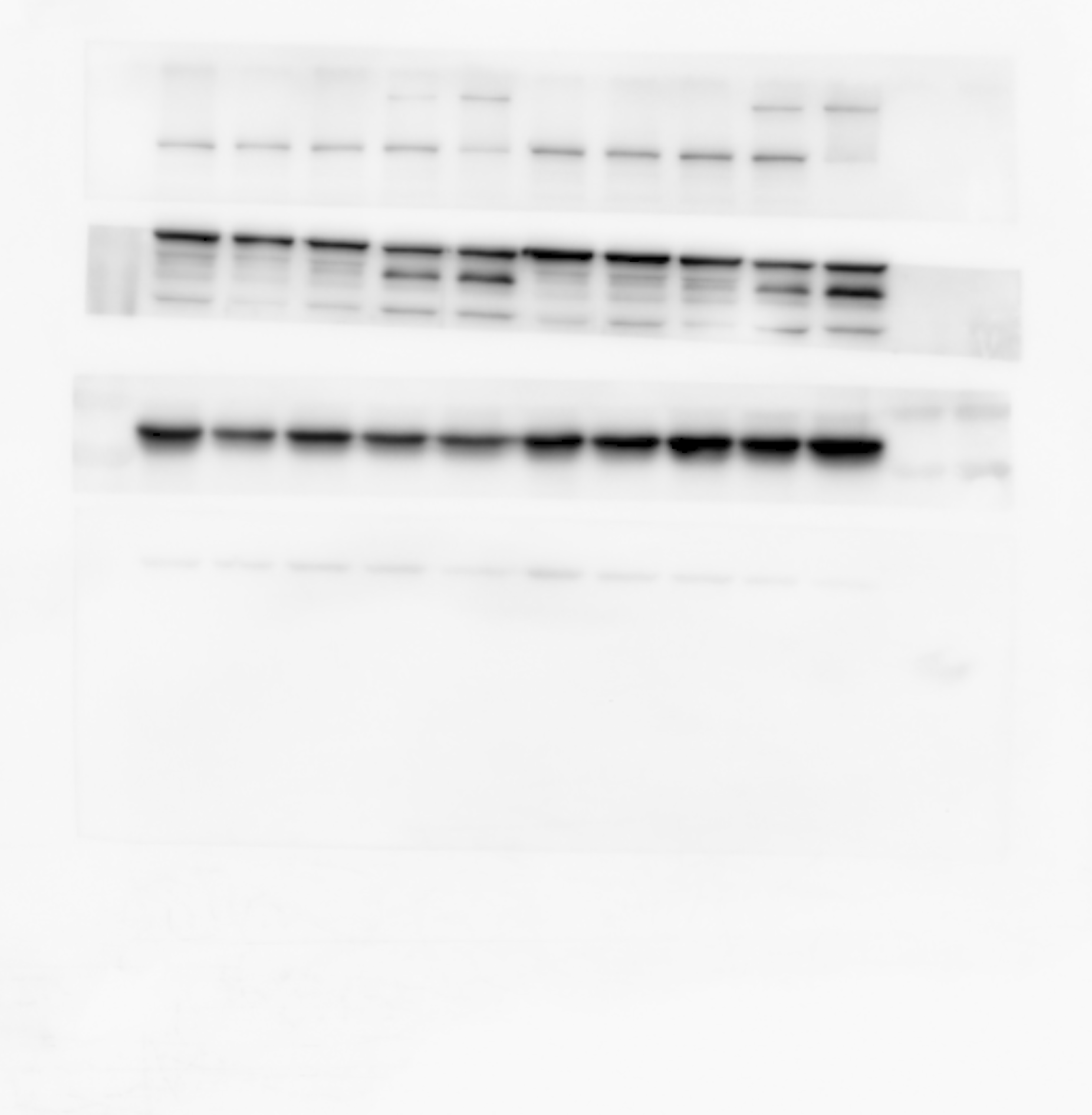

Supplement: Figure 1—figure supplement 1—source data 2. [file elife-99438-fig1-figsupp1-data2.zip › Figure 1-Figure supplement 1-source data 2/Figure S1C anti PELO and VCP; 2023-06-20 12h17m01s.tif]

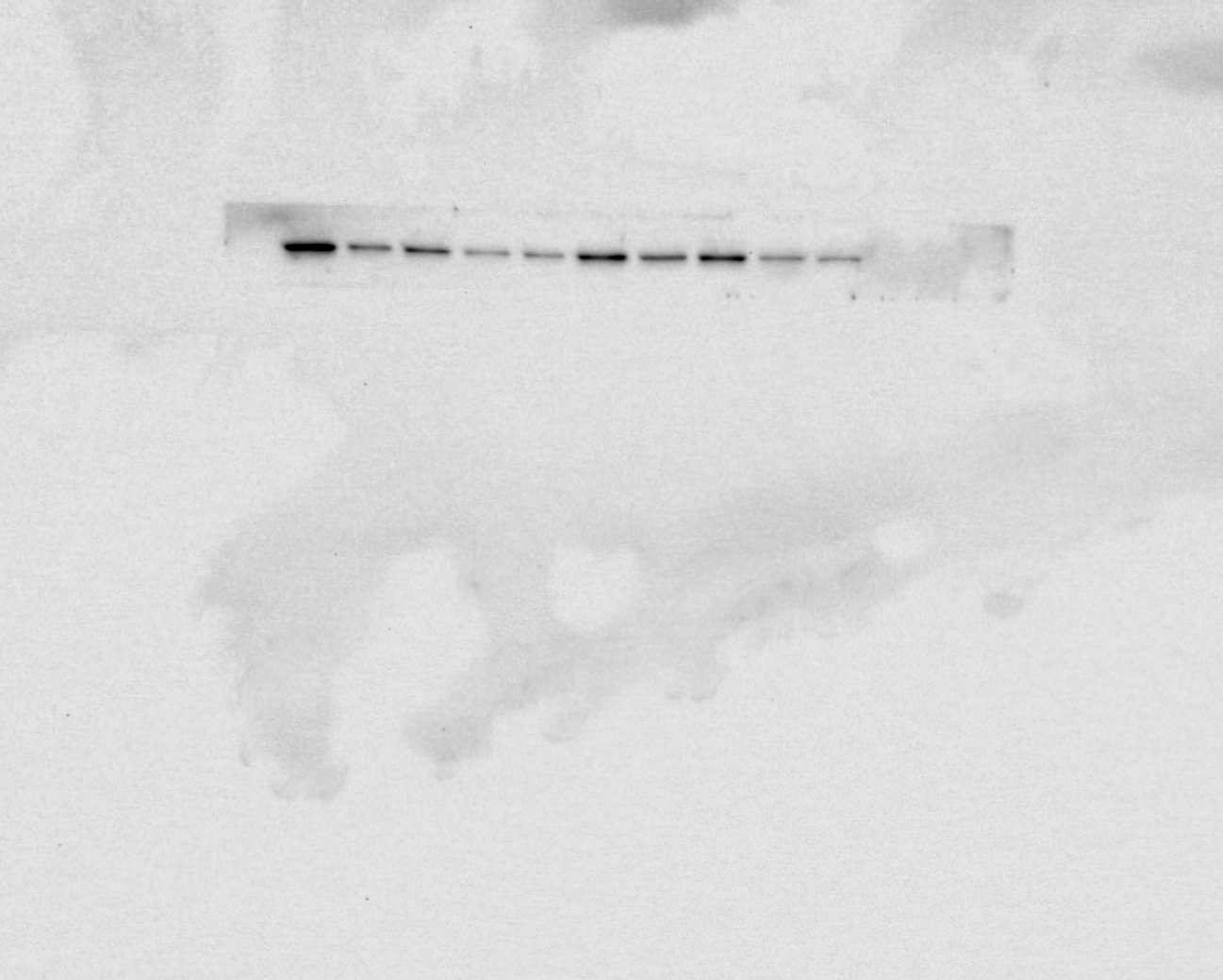

Supplement: Figure 1—figure supplement 1—source data 2. [file elife-99438-fig1-figsupp1-data2.zip › Figure 1-Figure supplement 1-source data 2/Figure S1C anti ANKZF1; Ting 2023-06-20 12h32m42s.tif]

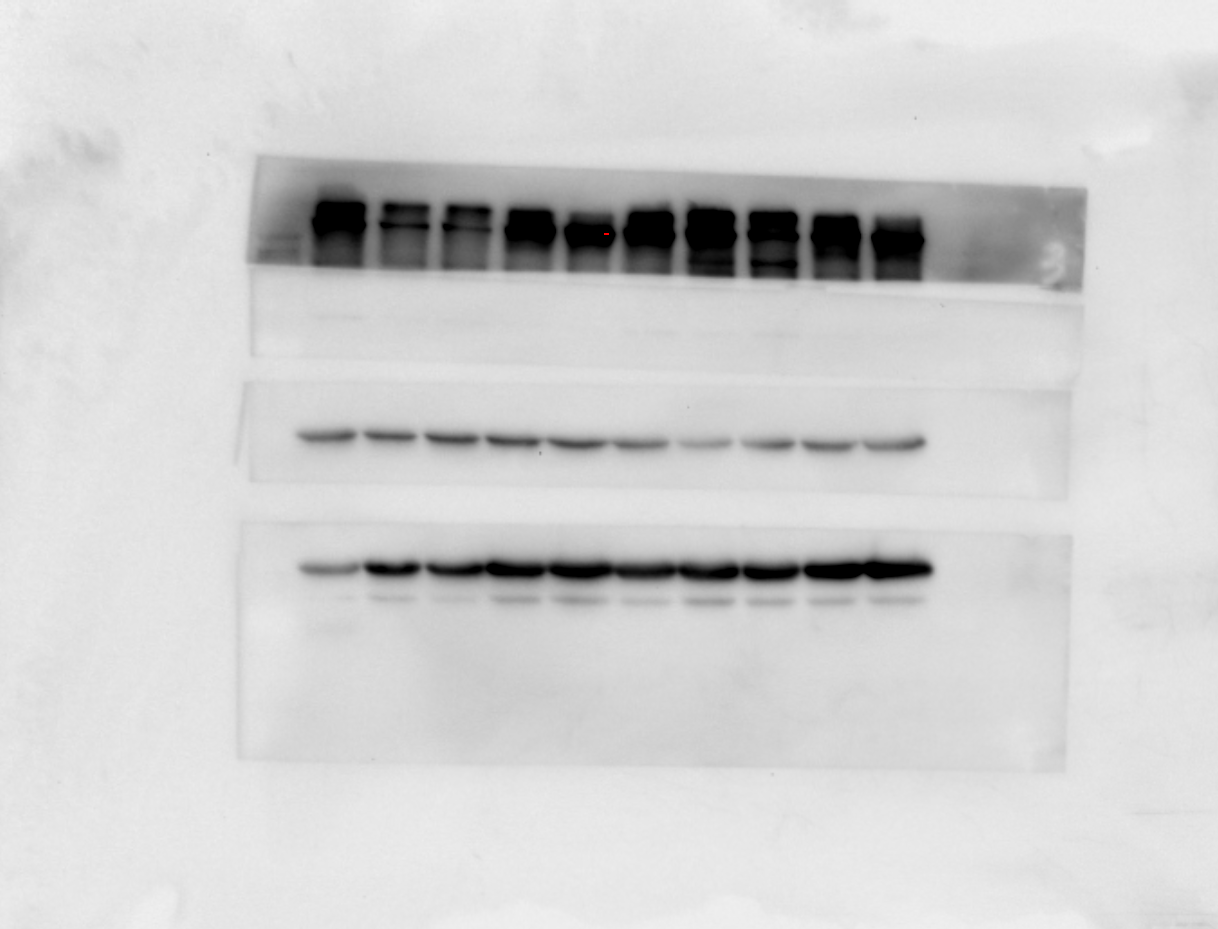

Supplement: Figure 1—figure supplement 1—source data 2. [file elife-99438-fig1-figsupp1-data2.zip › Figure 1-Figure supplement 1-source data 2/Figure S1C anti GAPDH; Ting 2023-06-20 12h18m13s.tif]

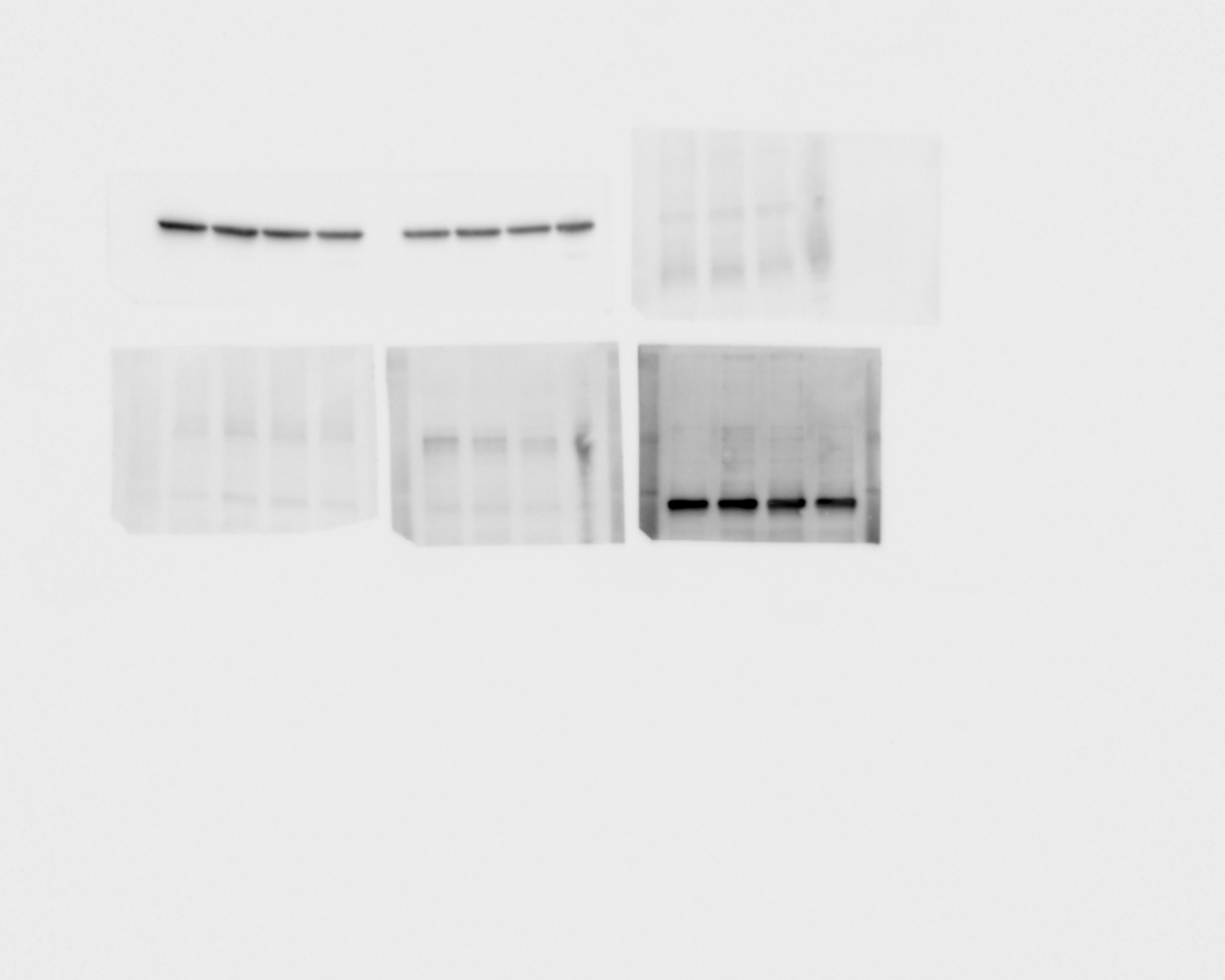

Supplement: Figure 1—figure supplement 1—source data 2. [file elife-99438-fig1-figsupp1-data2.zip › Figure 1-Figure supplement 1-source data 2/Figure S1C anti ASCC2; 2025-04-30 13h02m20s.tif]

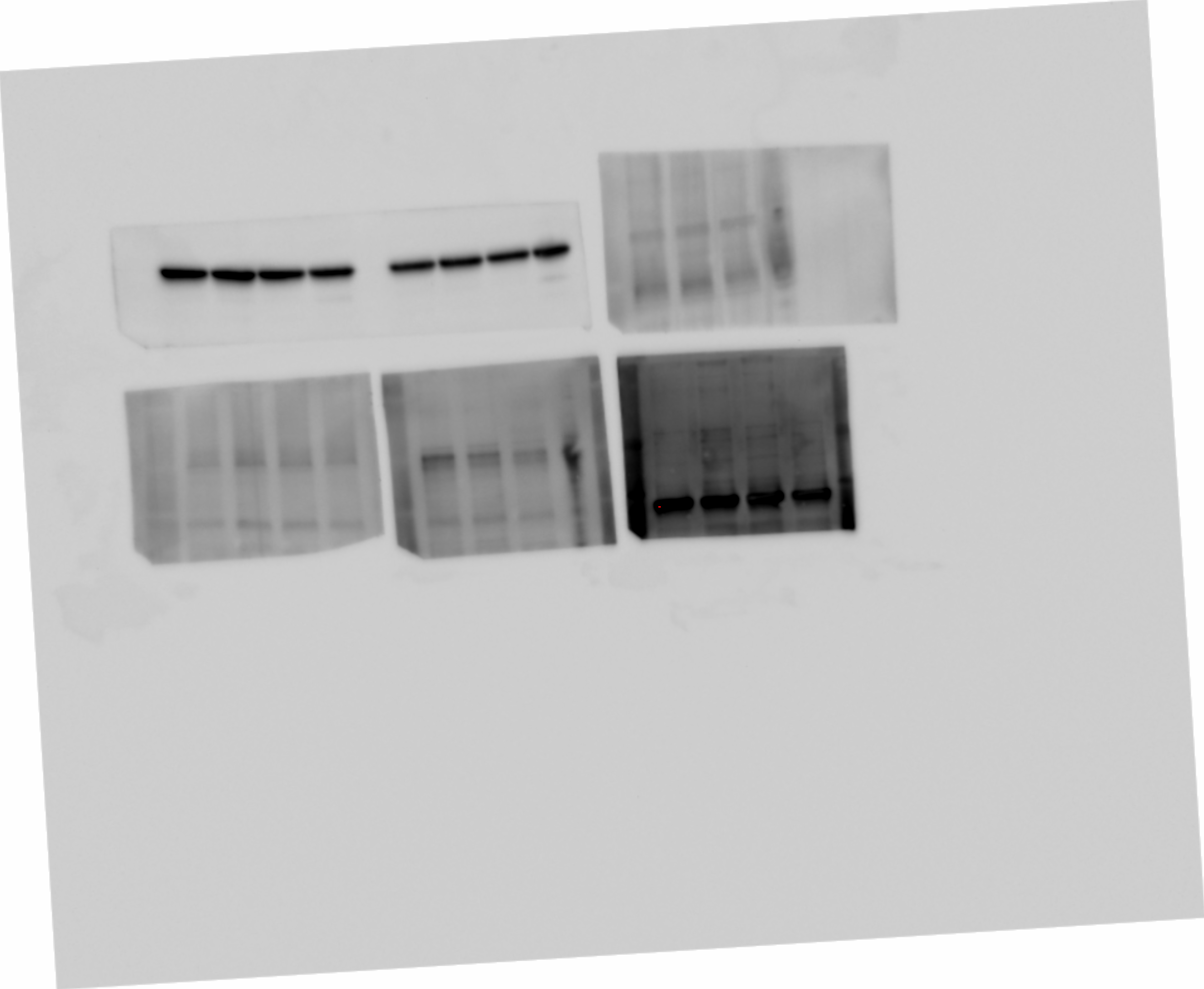

Supplement: Figure 1—figure supplement 1—source data 2. [file elife-99438-fig1-figsupp1-data2.zip › Figure 1-Figure supplement 1-source data 2/Figure S1C anti ACTIN; 2025-04-30 13h04m01s.tif]

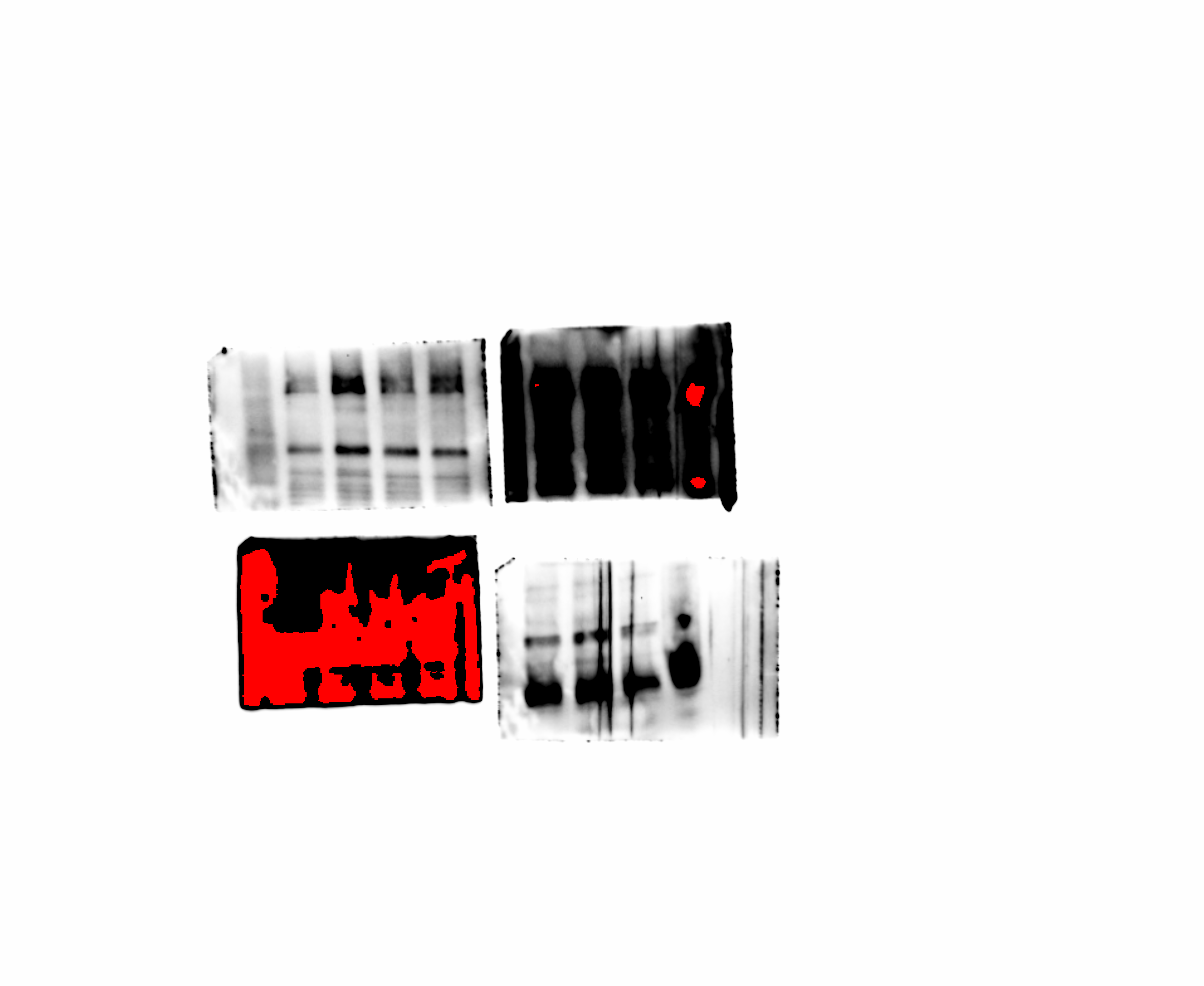

Supplement: Figure 1—figure supplement 1—source data 2. [file elife-99438-fig1-figsupp1-data2.zip › Figure 1-Figure supplement 1-source data 2/Figure S1C anti NEMF; 2025-05-01 12h54m56s.tif]

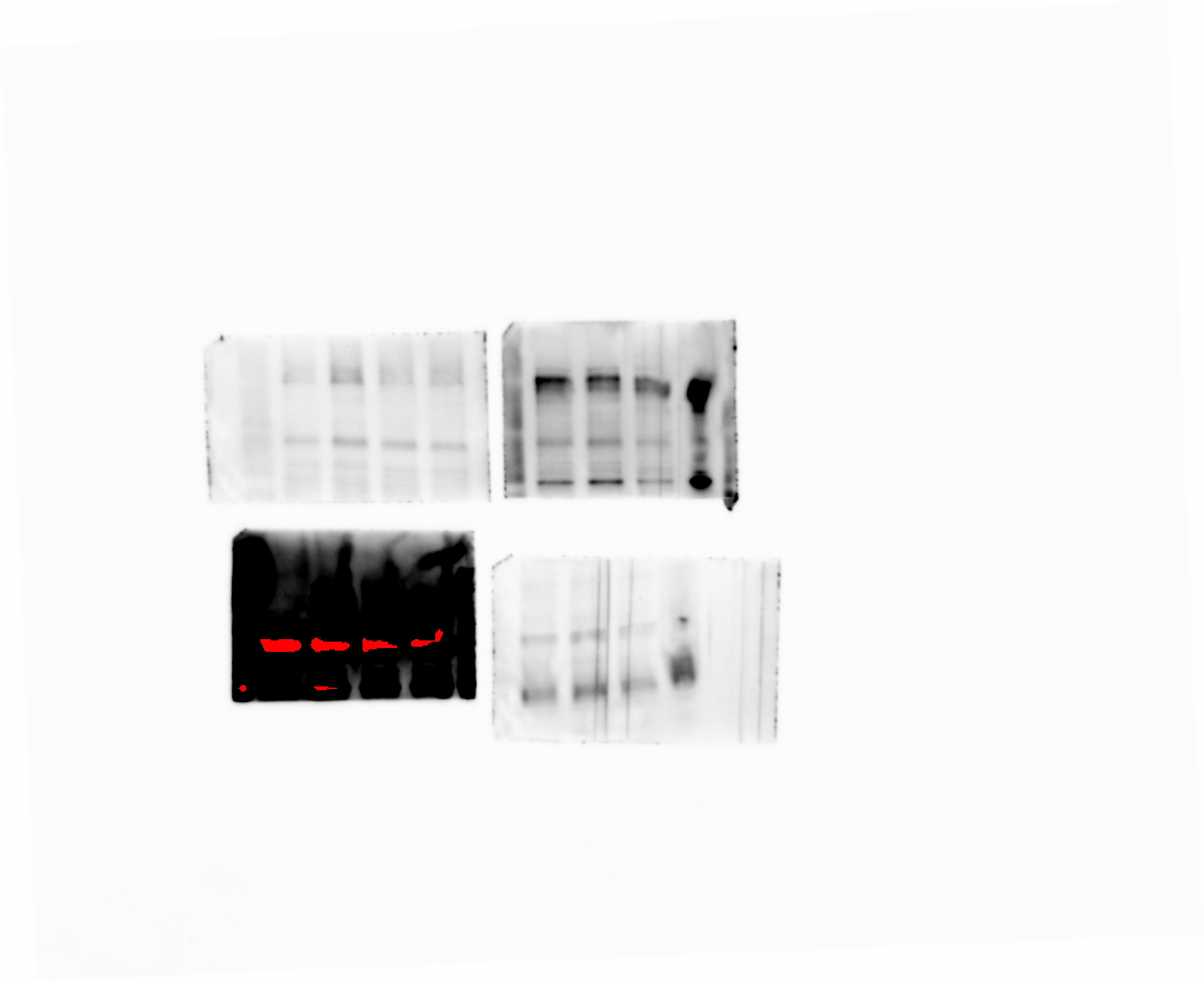

Supplement: Figure 1—figure supplement 1—source data 2. [file elife-99438-fig1-figsupp1-data2.zip › Figure 1-Figure supplement 1-source data 2/Figure S1C anti ZNF598; 2025-05-01 12h54m11s.tif]

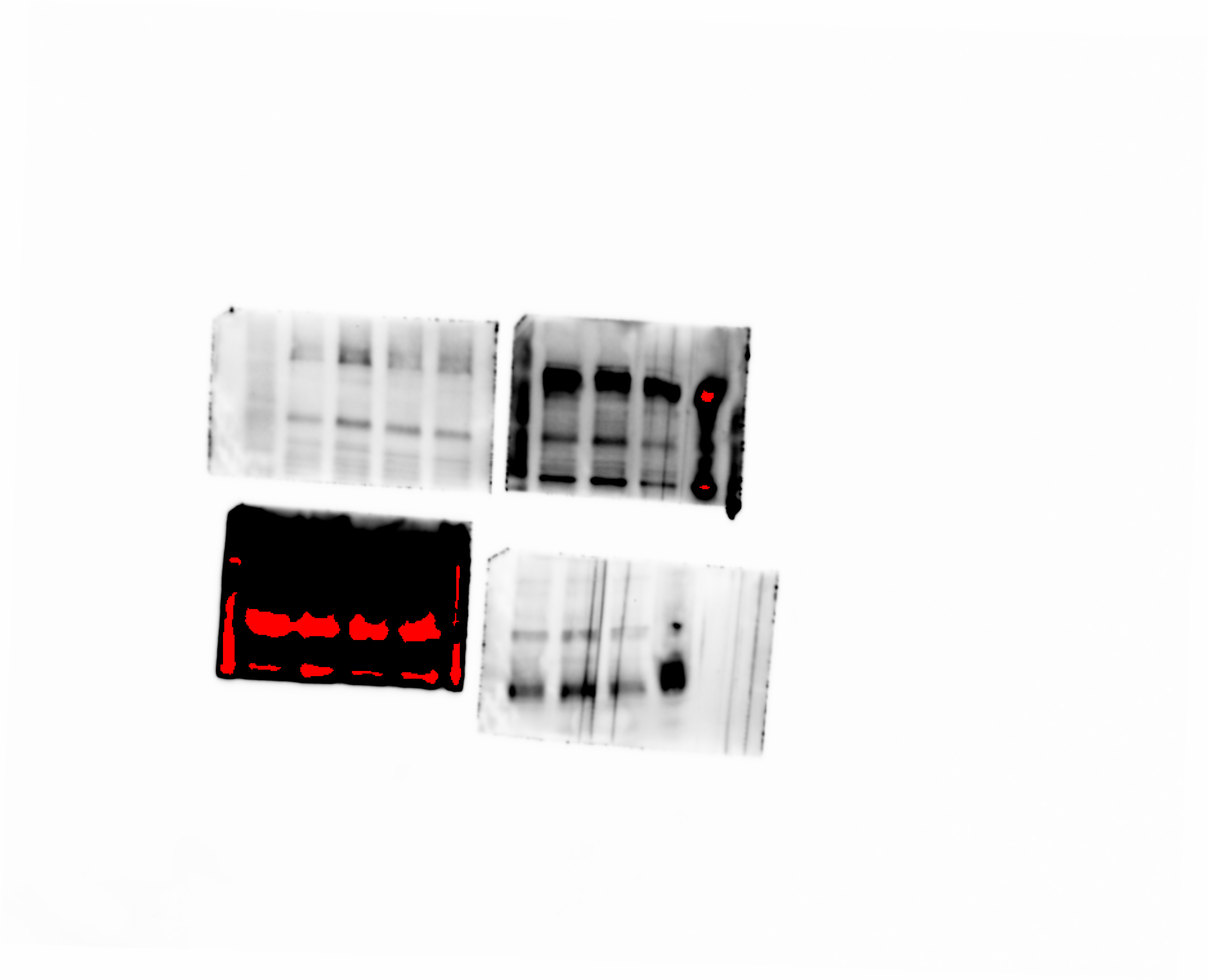

Supplement: Figure 1—figure supplement 1—source data 2. [file elife-99438-fig1-figsupp1-data2.zip › Figure 1-Figure supplement 1-source data 2/Figure S1C anti ABCE1; 2025-05-01 12h54m33s.tif]

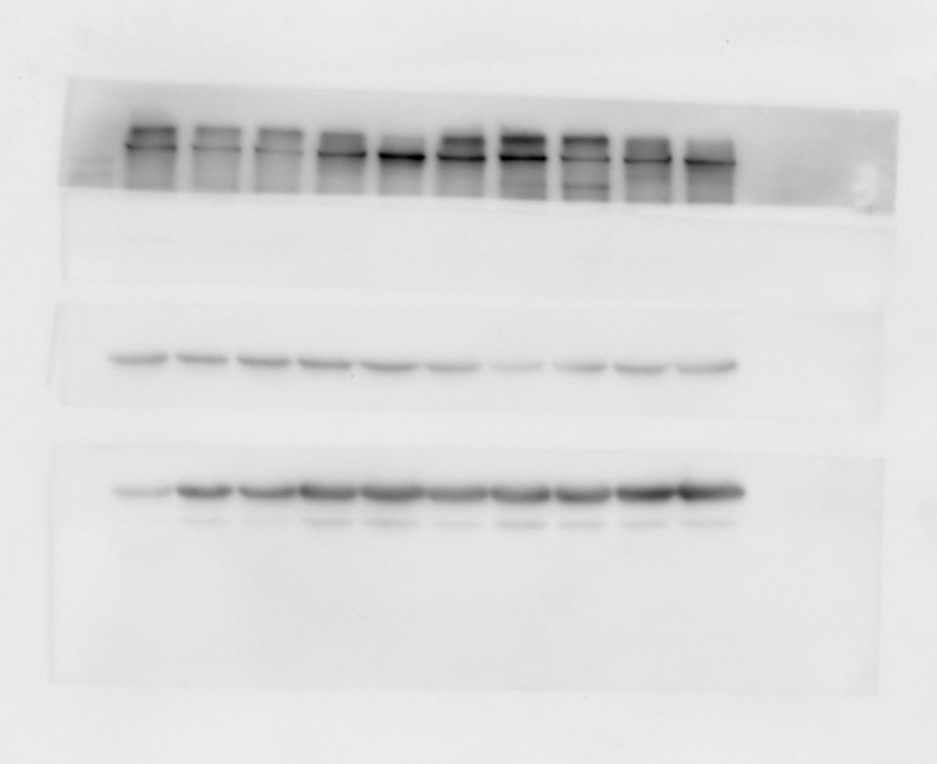

Supplement: Figure 1—figure supplement 1—source data 2. [file elife-99438-fig1-figsupp1-data2.zip › Figure 1-Figure supplement 1-source data 2/Figure S1C anti ASCC3; Ting 2023-06-20 12h18m13s.jpg]

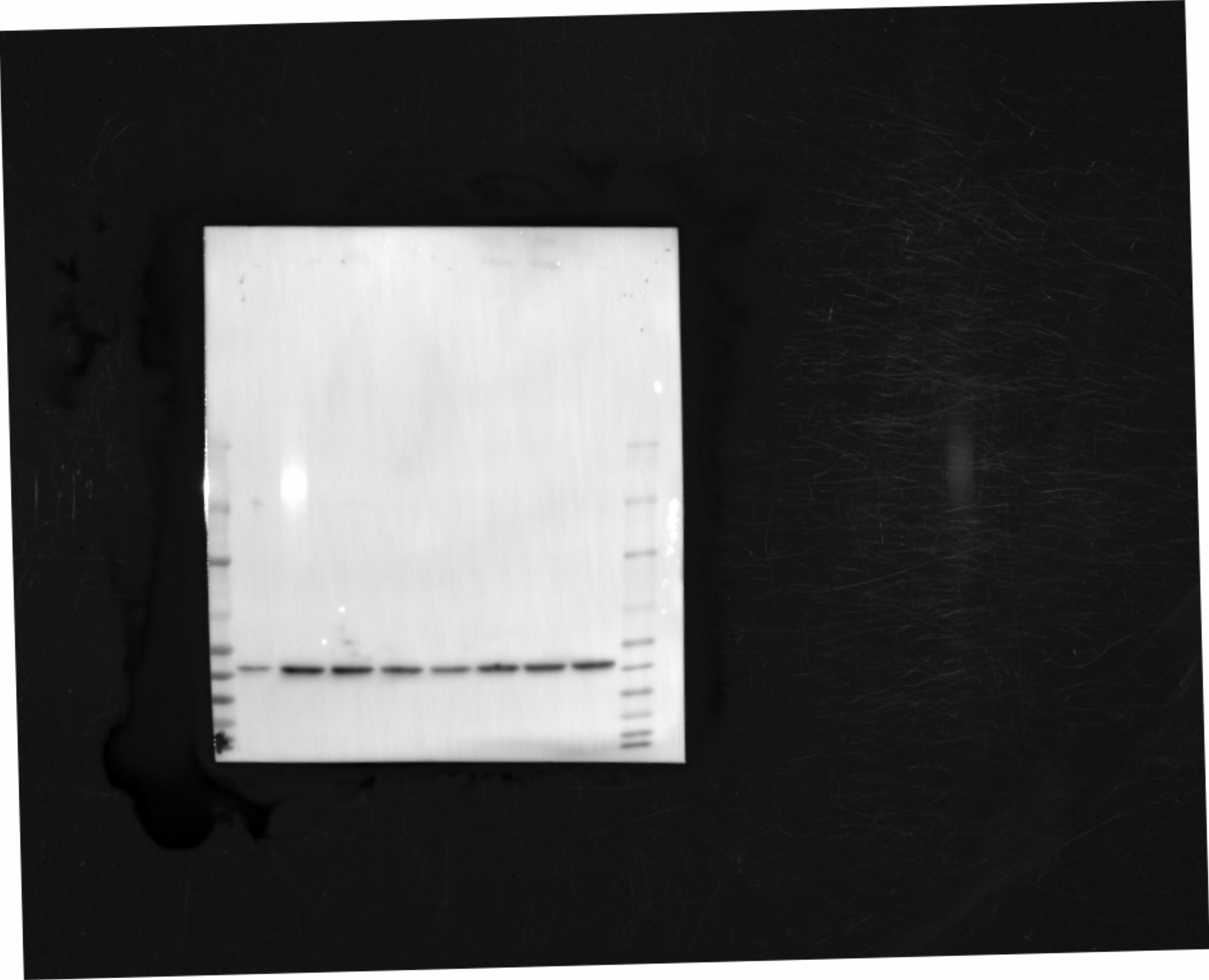

Supplement: Figure 1—figure supplement 2—source data 2. [file elife-99438-fig1-figsupp2-data2.zip › Figure 1-Figure supplement 2-source data 2/Figure S2E GS anti actin; 2025-01-16 13h17m38s.tif]

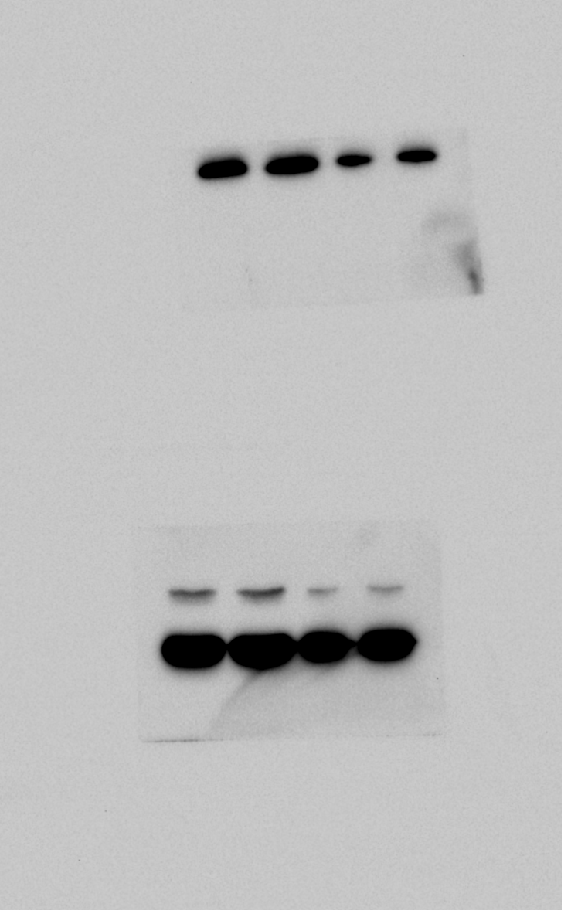

Supplement: Figure 1—figure supplement 2—source data 2. [file elife-99438-fig1-figsupp2-data2.zip › Figure 1-Figure supplement 2-source data 2/Figure S2A anti ATP5a short; 2026-01-06 13h55m27s.tif]

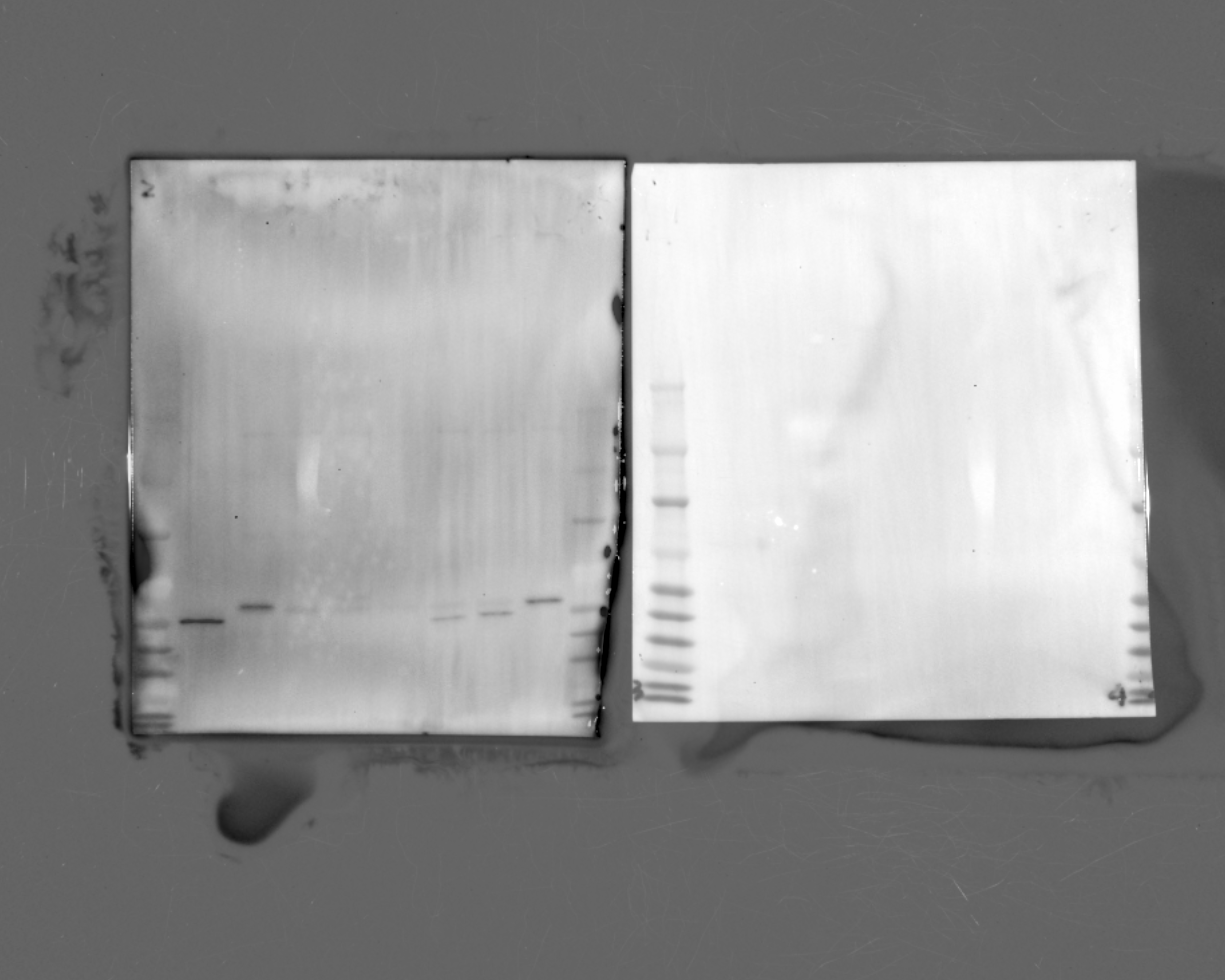

Supplement: Figure 1—figure supplement 2—source data 2. [file elife-99438-fig1-figsupp2-data2.zip › Figure 1-Figure supplement 2-source data 2/Figure S2E GS anti flag; 2025-01-16 13h04m44s.tif]

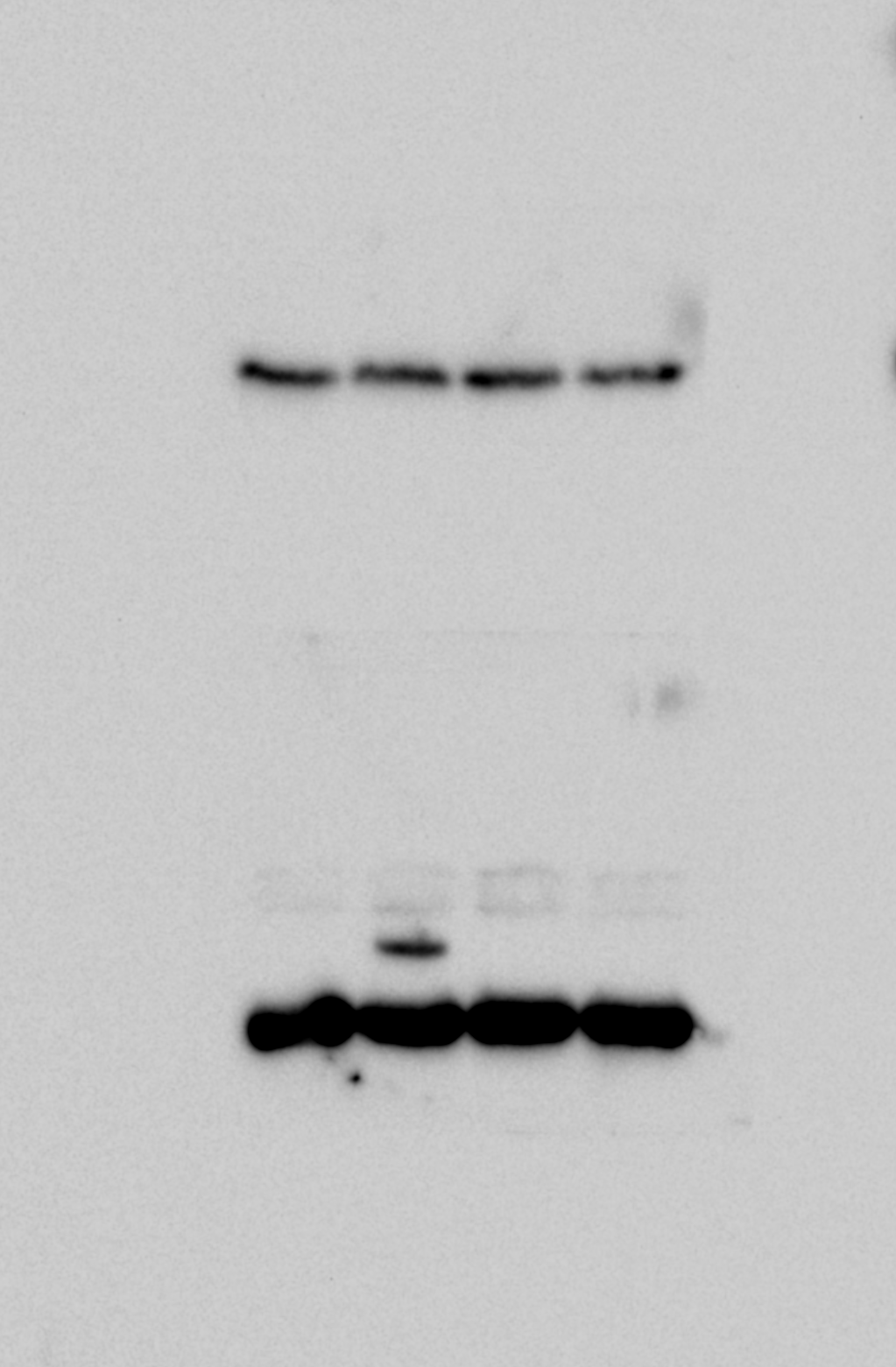

Supplement: Figure 1—figure supplement 2—source data 2. [file elife-99438-fig1-figsupp2-data2.zip › Figure 1-Figure supplement 2-source data 2/Figure S2B anti ATP5a, Actin; 2025-12-31 15h27m46s.tif]

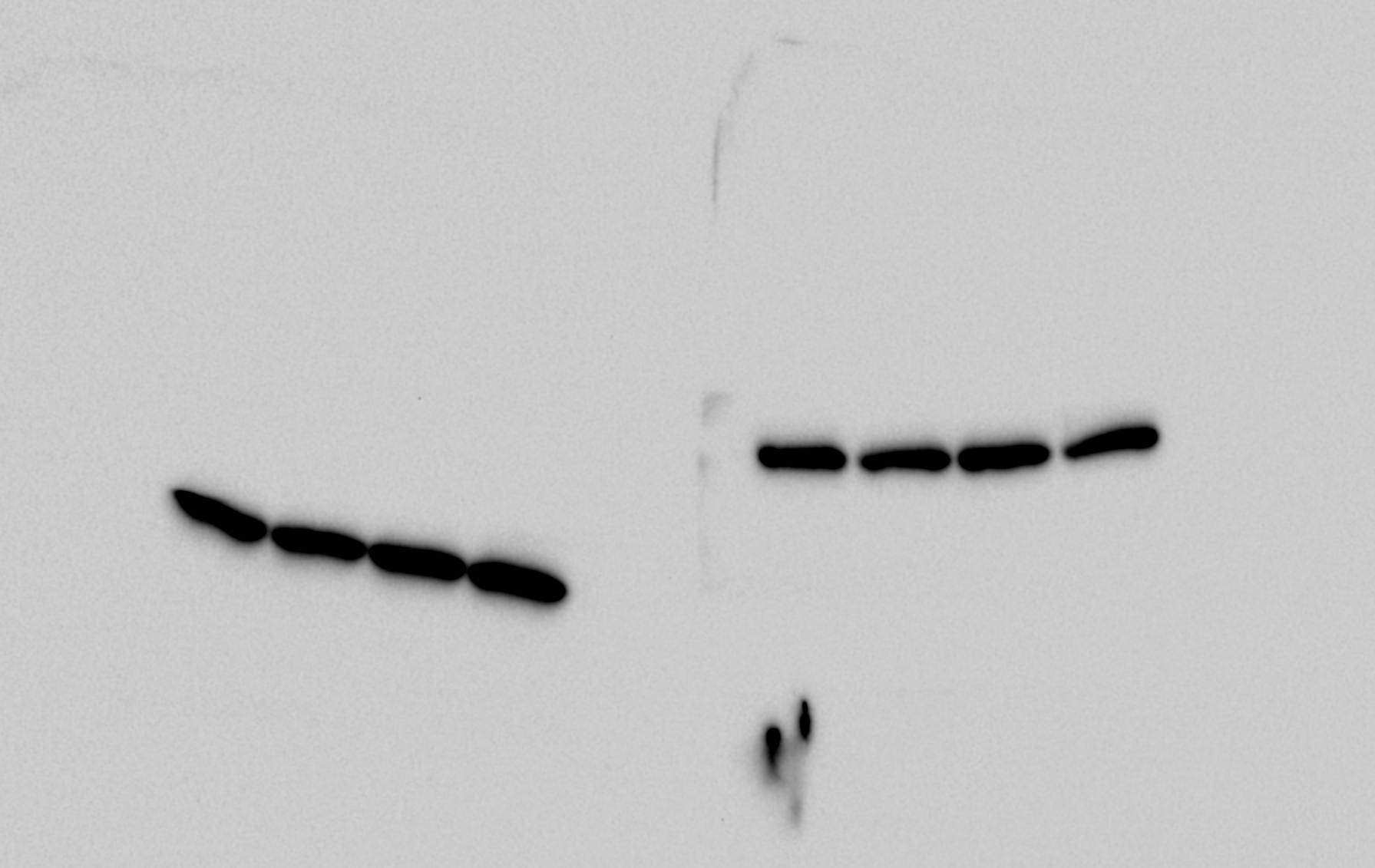

Supplement: Figure 1—figure supplement 2—source data 2. [file elife-99438-fig1-figsupp2-data2.zip › Figure 1-Figure supplement 2-source data 2/Figure S2A anti GAPDH long; 2025-12-31 15h27m46s.tif]

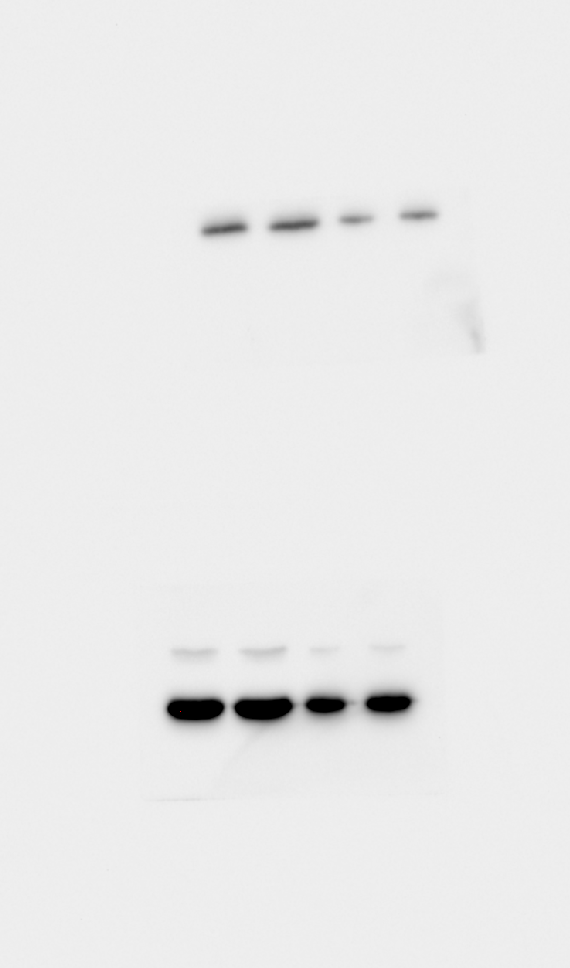

Supplement: Figure 1—figure supplement 2—source data 2. [file elife-99438-fig1-figsupp2-data2.zip › Figure 1-Figure supplement 2-source data 2/Figure S2A anti ATP5a long; 2026-01-06 13h54m56s.tif]

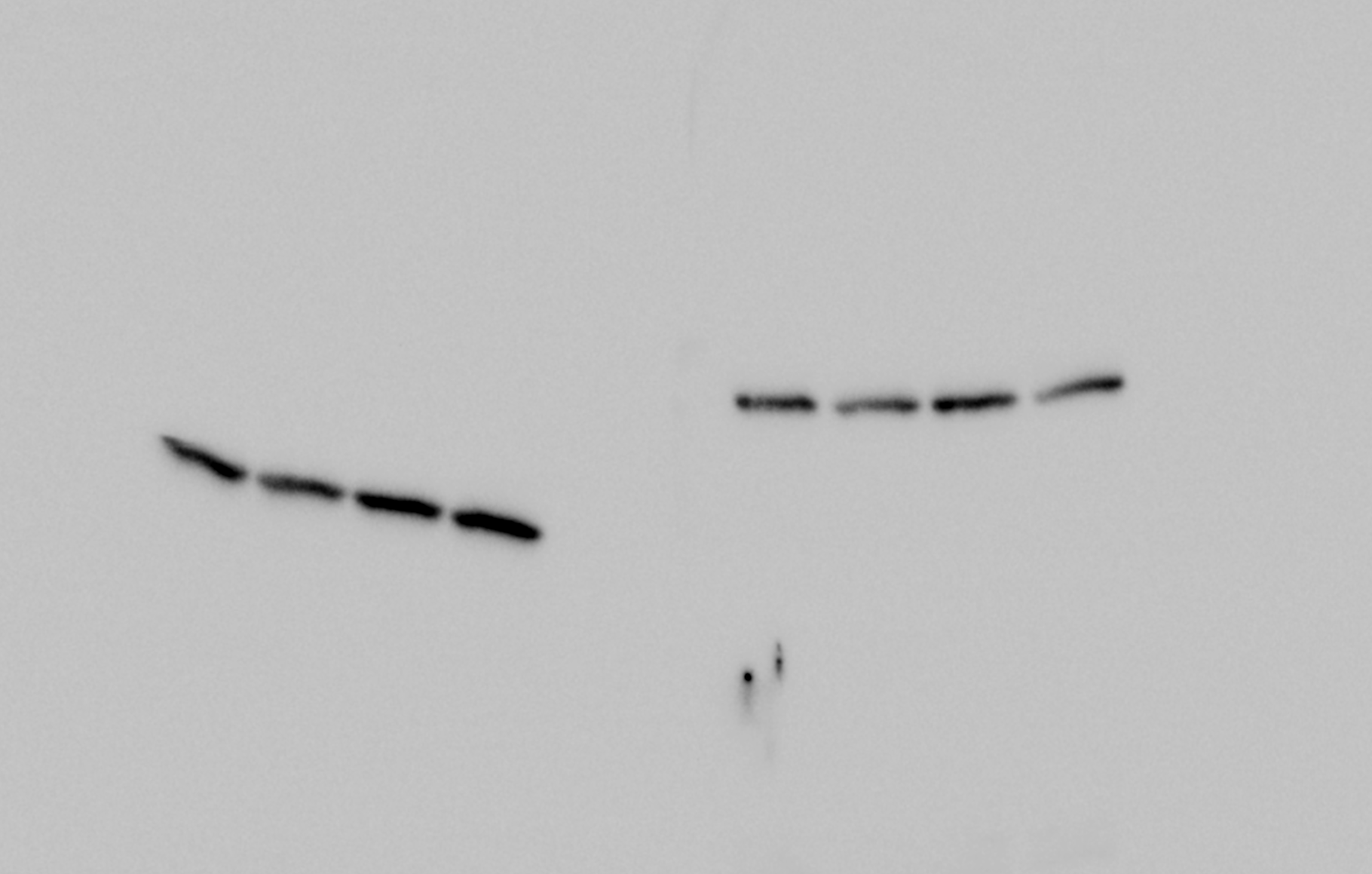

Supplement: Figure 1—figure supplement 2—source data 2. [file elife-99438-fig1-figsupp2-data2.zip › Figure 1-Figure supplement 2-source data 2/Figure S2A anti GAPDH short; 2025-12-31 15h25m39s.tif]

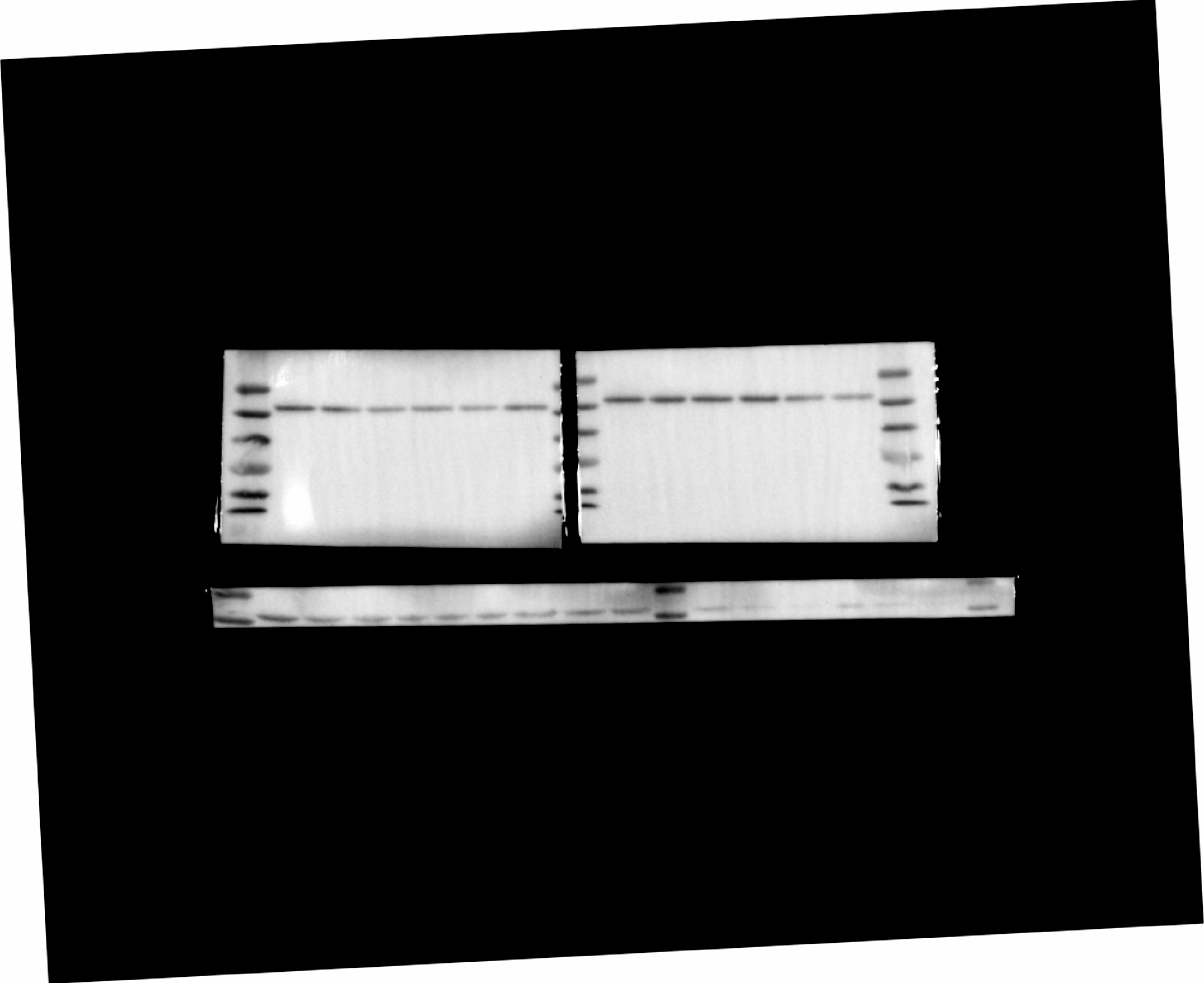

Supplement: Figure 2—source data 2. [file elife-99438-fig2-data2.zip › Figure 2-source data 2/Figure 2F anti actin; 2024-06-19 12h12m13s.tif]

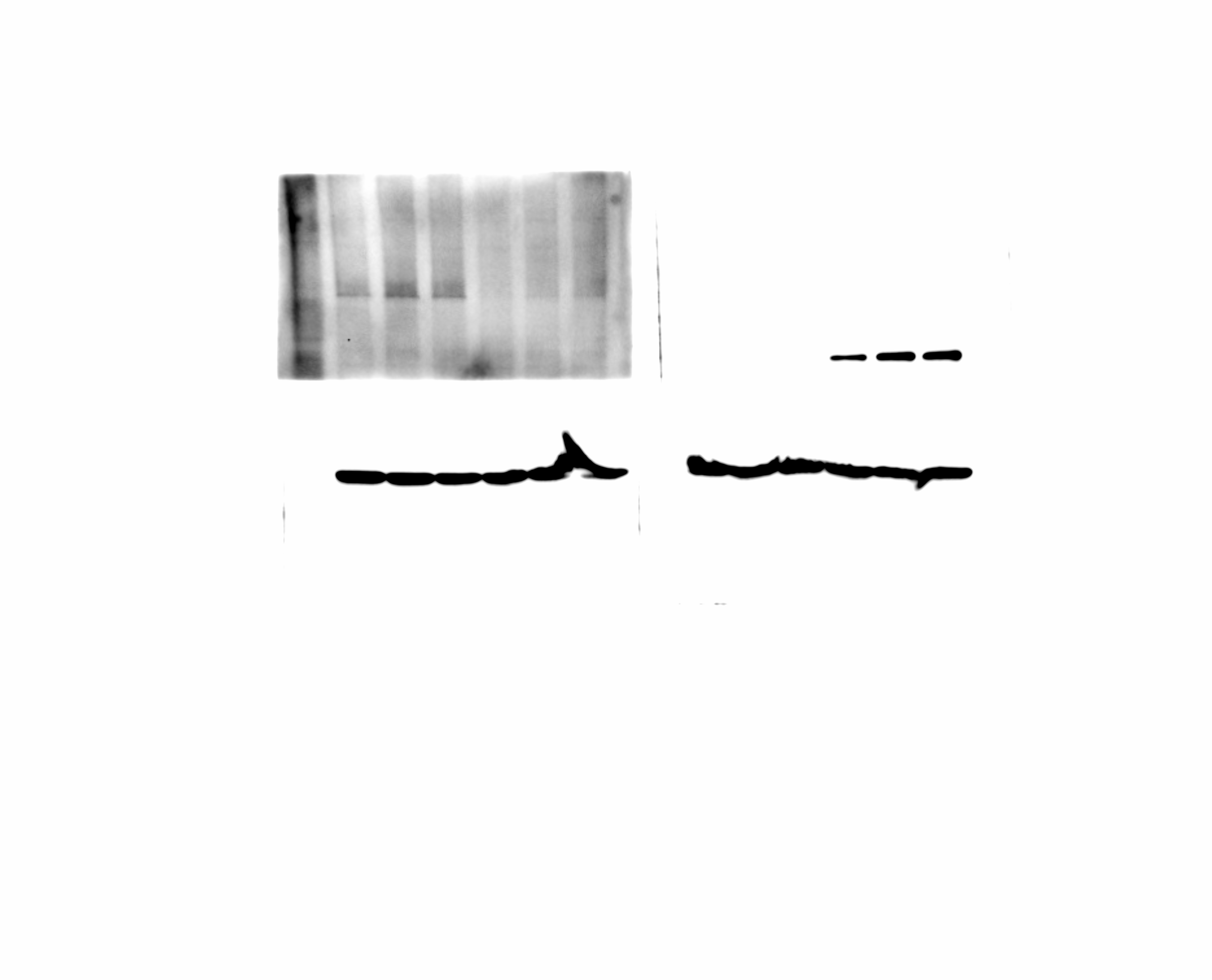

Supplement: Figure 2—source data 2. [file elife-99438-fig2-data2.zip › Figure 2-source data 2/Figure 2F anti nemf; 2024-06-10 15h05m49s.tif]

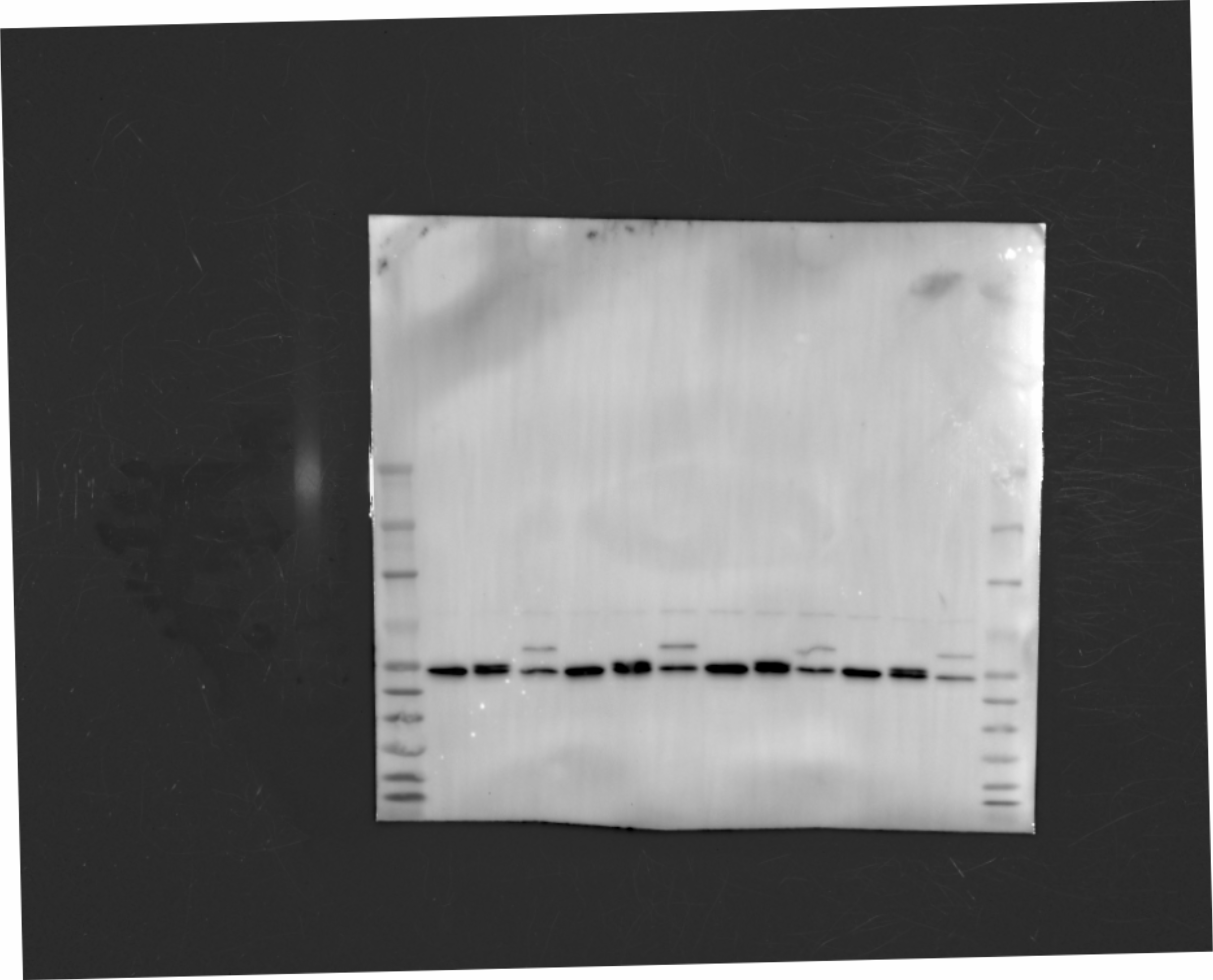

Supplement: Figure 2—source data 2. [file elife-99438-fig2-data2.zip › Figure 2-source data 2/Figure 2F anti atp5a; 2024-06-19 11h38m57s.tif]

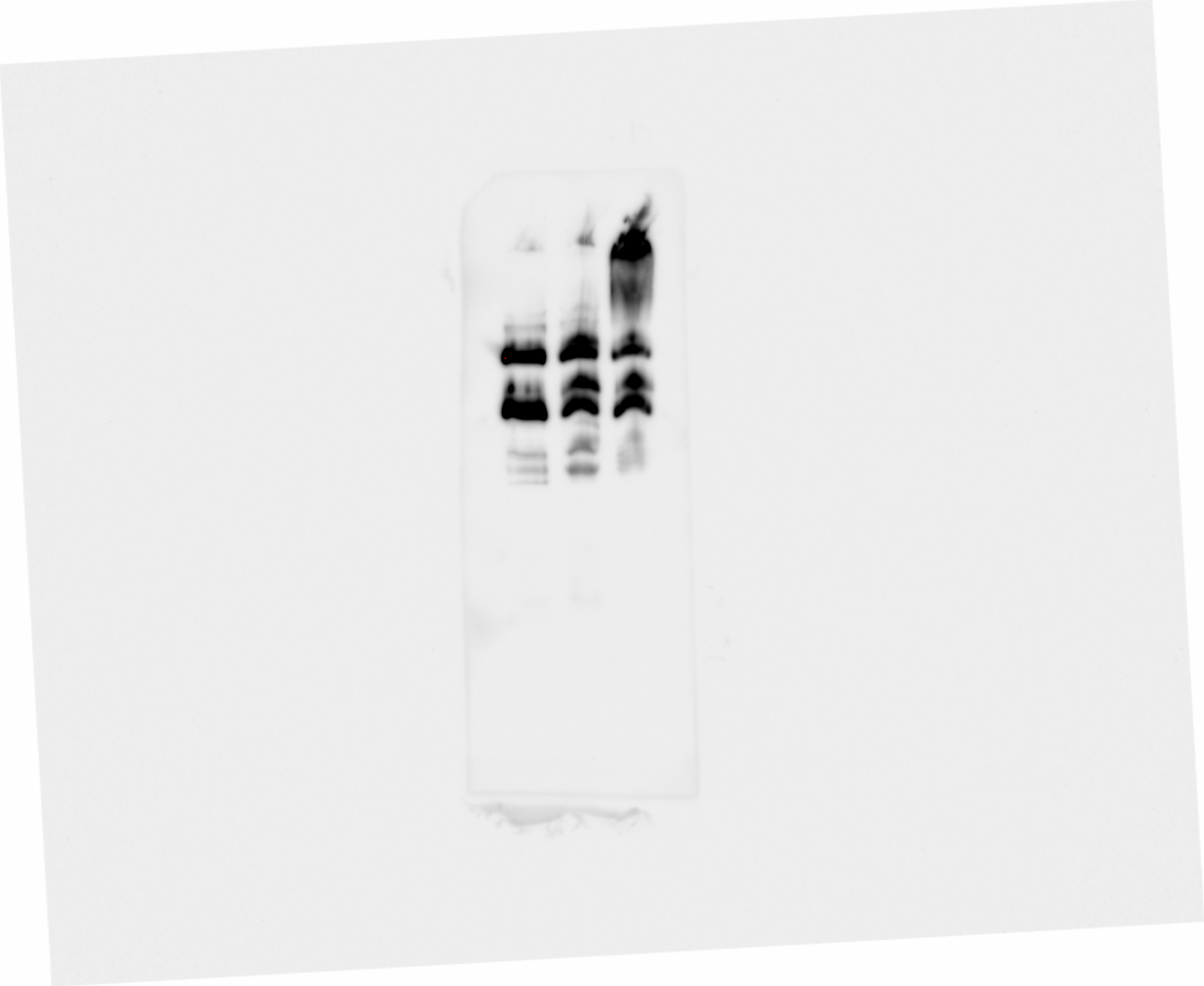

Supplement: Figure 2—source data 2. [file elife-99438-fig2-data2.zip › Figure 2-source data 2/Figure 2H SF anti atp5a; 2024-03-14 13h12m30s.tif]

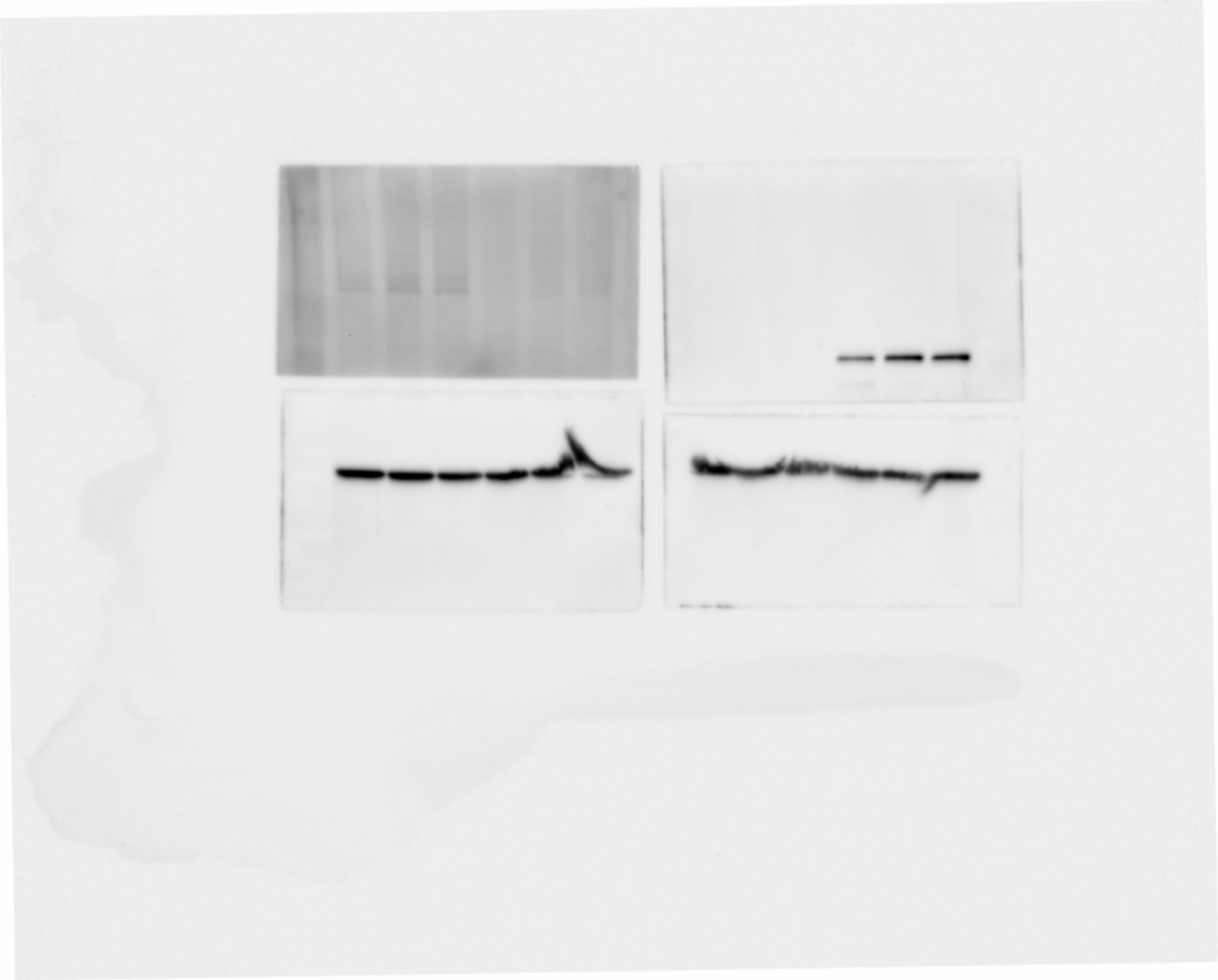

Supplement: Figure 2—source data 2. [file elife-99438-fig2-data2.zip › Figure 2-source data 2/Figure 2F anti ankzf1; 2024-06-10 15h04m10s.tif]

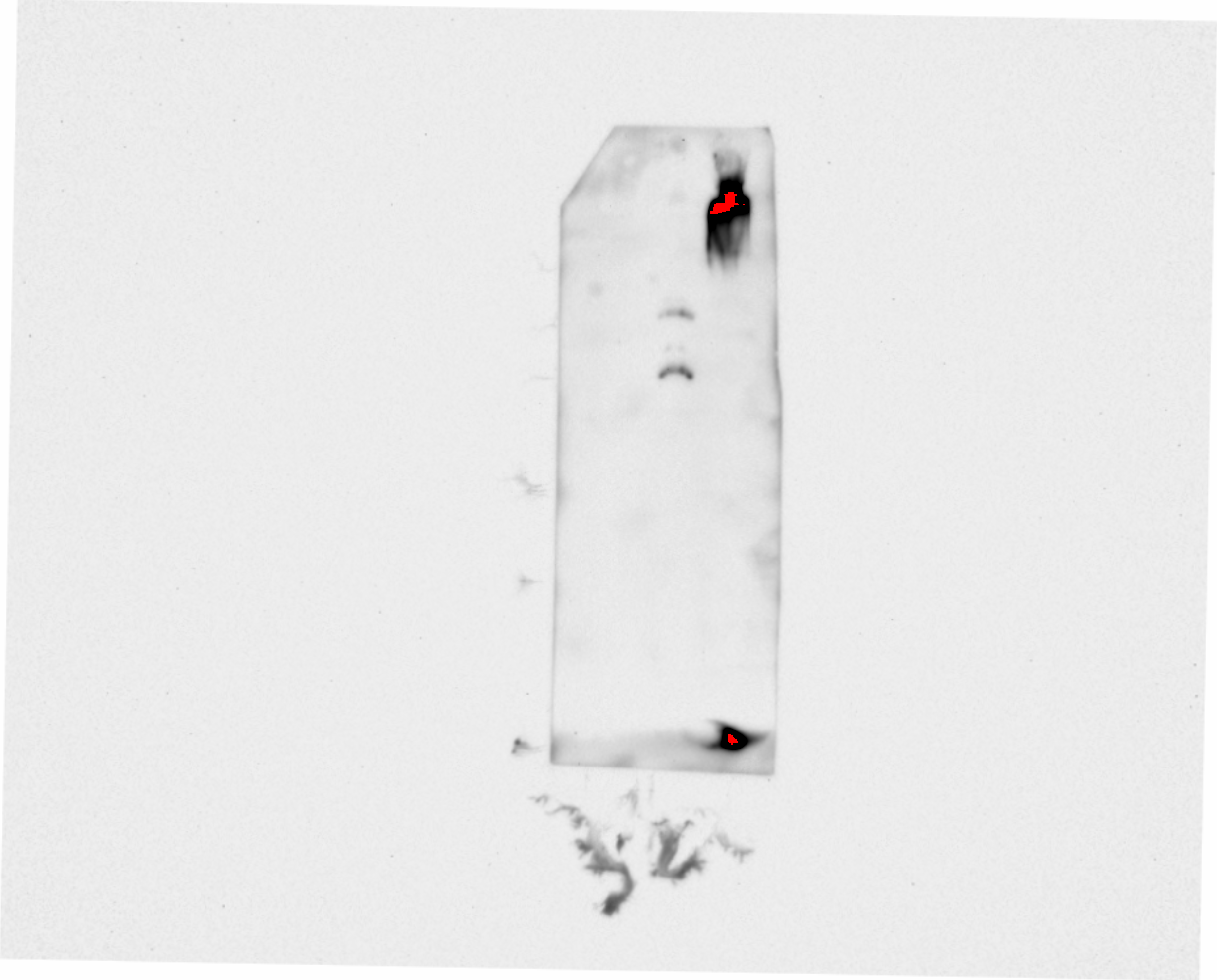

Supplement: Figure 2—source data 2. [file elife-99438-fig2-data2.zip › Figure 2-source data 2/Figure 2H SF anti flag; 2024-03-14 13h01m53s.tif]

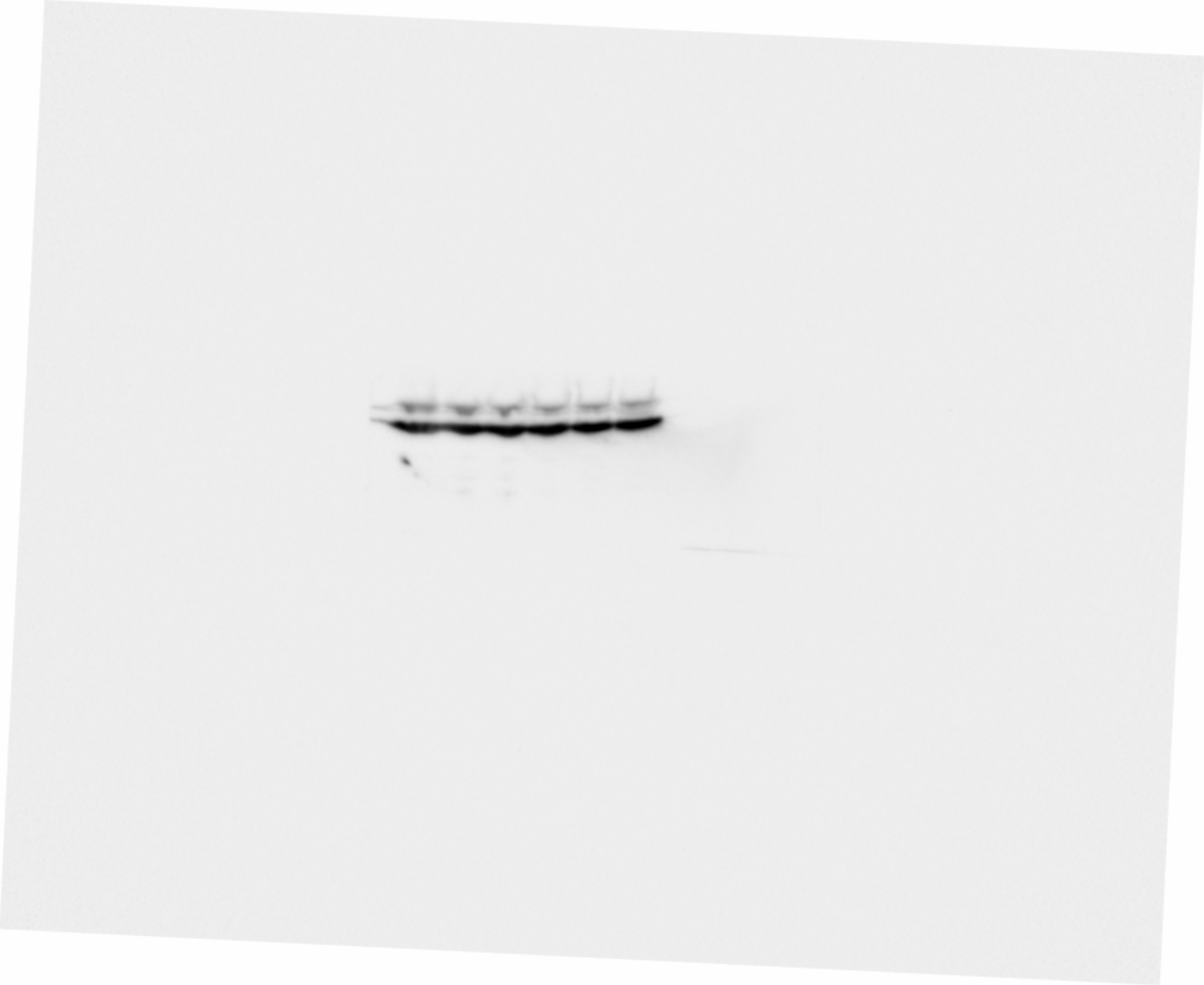

Supplement: Figure 2—figure supplement 1—source data 2. [file elife-99438-fig2-figsupp1-data2.zip › Figure 2-Figure supplement 1-source data 2/Figure S4D anti actin; 2024-04-01 12h43m49s.tif]

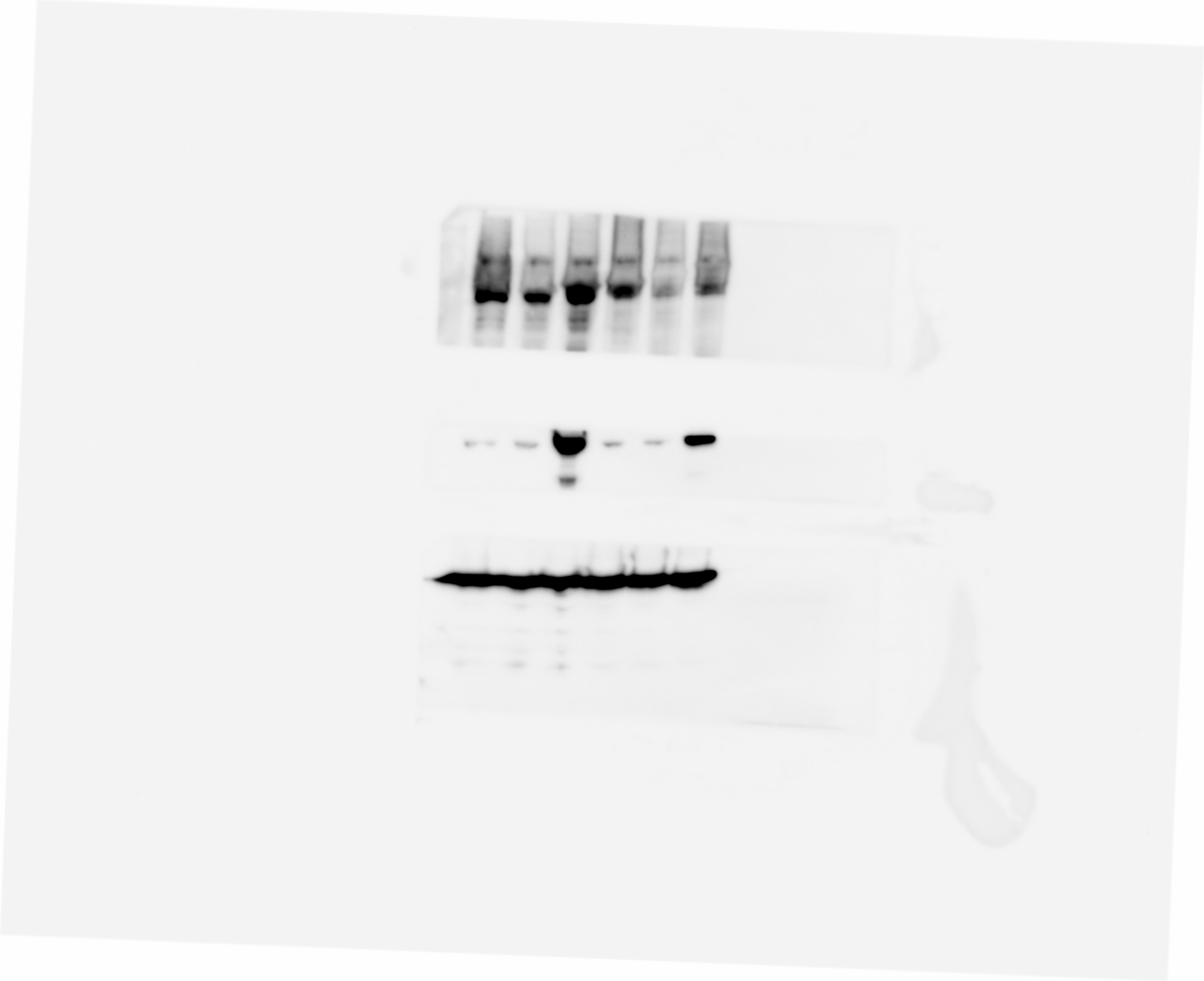

Supplement: Figure 2—figure supplement 1—source data 2. [file elife-99438-fig2-figsupp1-data2.zip › Figure 2-Figure supplement 1-source data 2/Figure S4D anti nemf; anti ankzf1; 2024-03-29 13h06m17s.tif]

Figure 3M

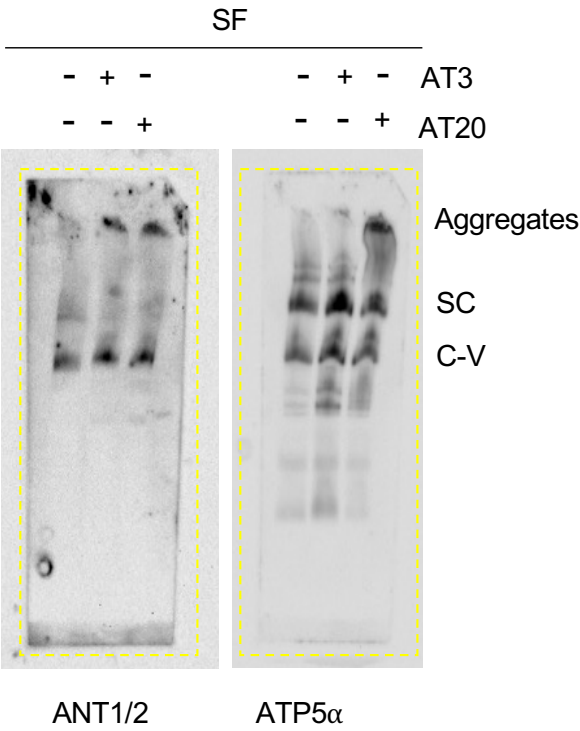

Figure 3, source data 1  
Original membranes corresponding to Figure 3M.

Supplement: Figure 3—source data 1. [file elife-99438-fig3-data1.zip › Figure 3-source data 1.pdf]

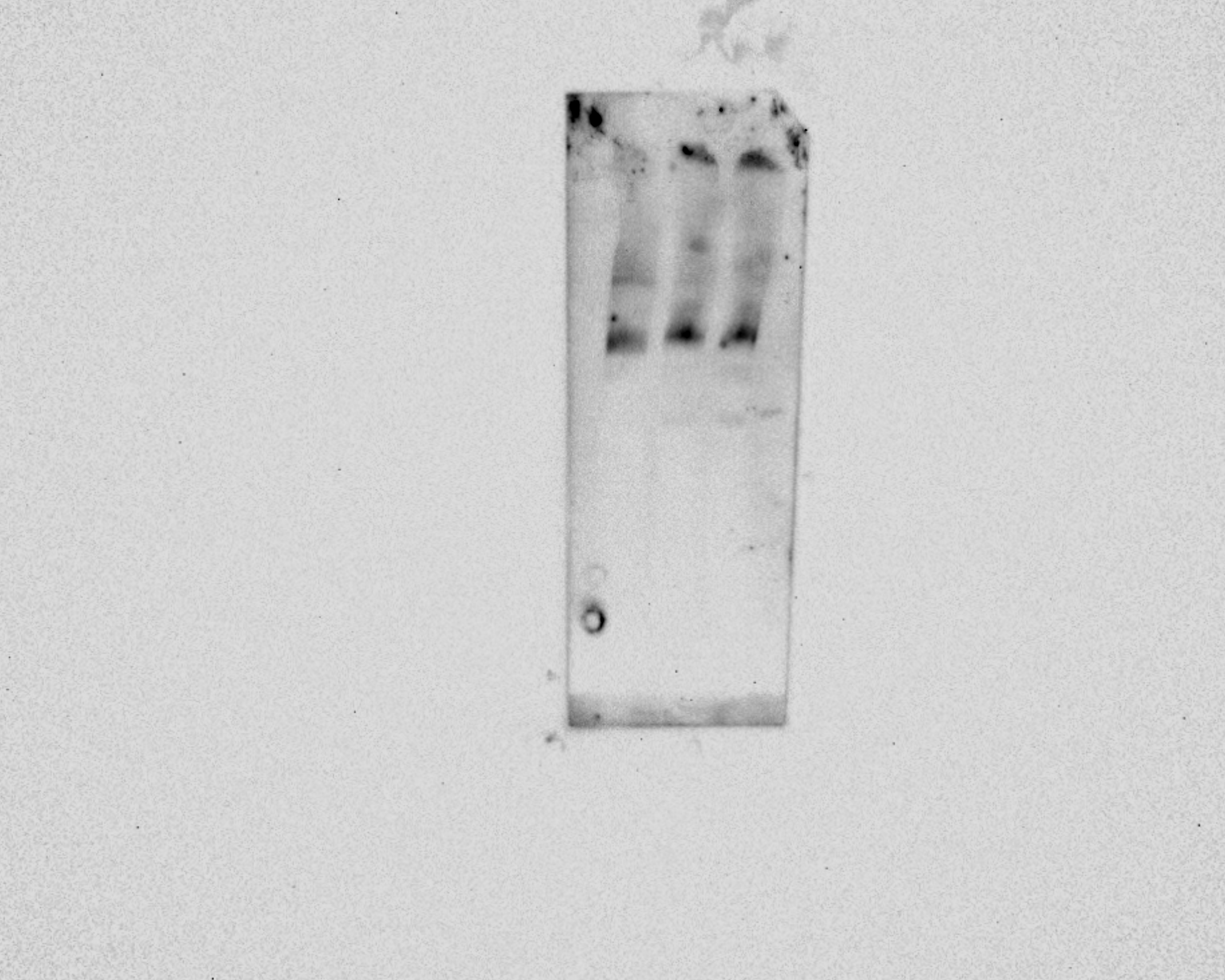

Supplement: Figure 3—source data 2. [file elife-99438-fig3-data2.zip › Figure 3_source data 2/Figure 3M anti ant; 2024-03-29 12h58m38s.tif]

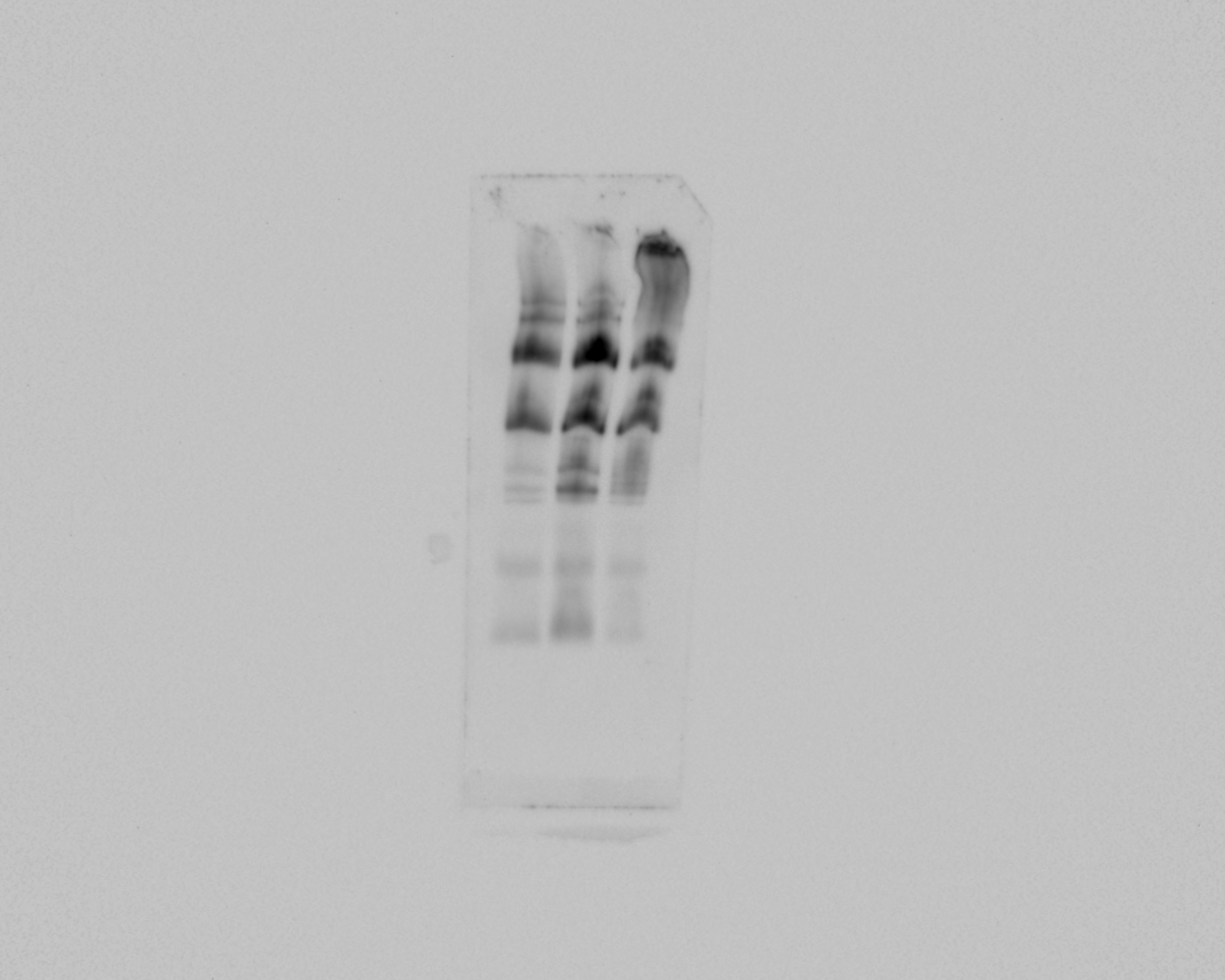

Supplement: Figure 3—source data 2. [file elife-99438-fig3-data2.zip › Figure 3_source data 2/Figure 3M anti atp5a; 2024-04-01 13h03m52s.tif]

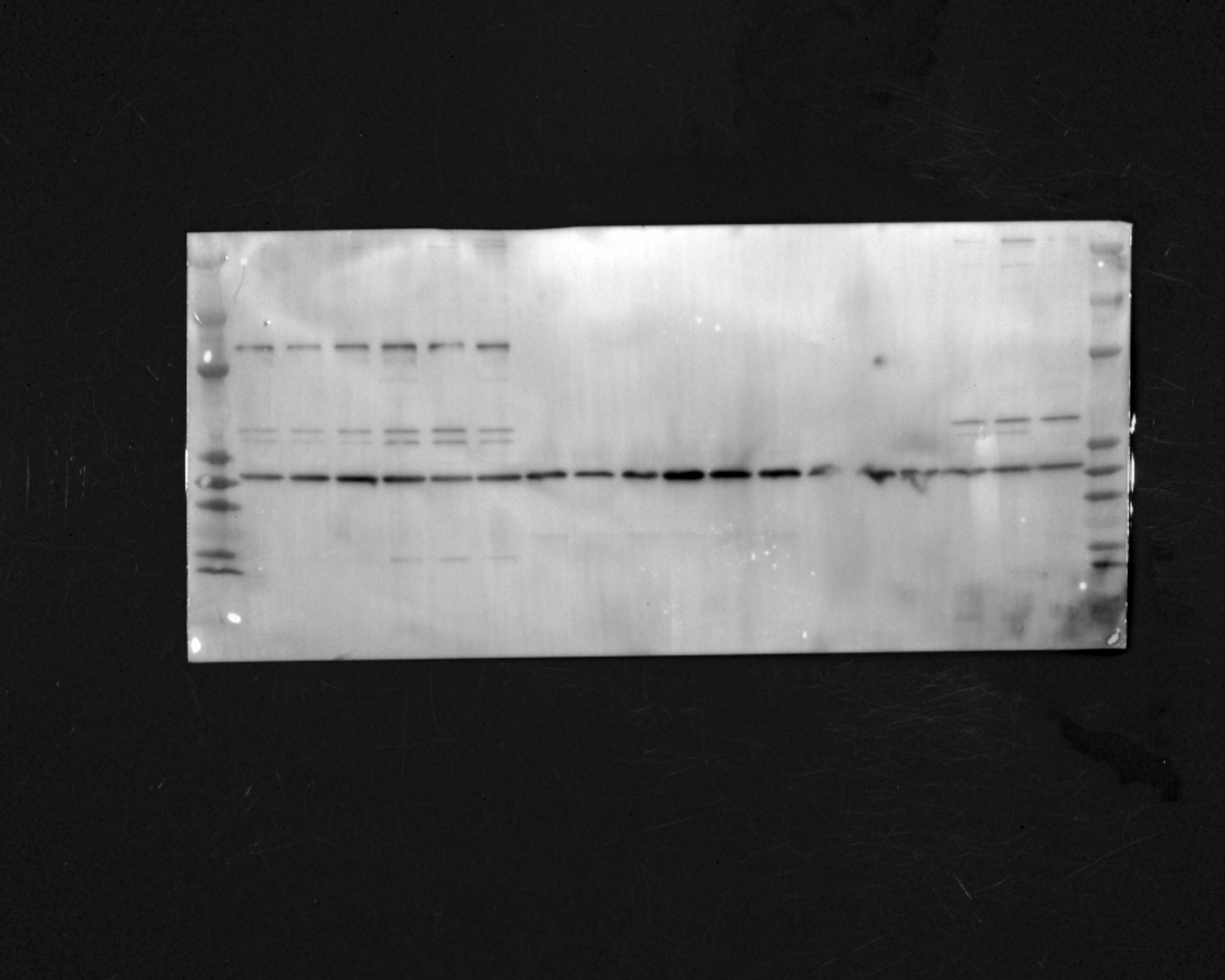

Supplement: Figure 3—figure supplement 2—source data 2. [file elife-99438-fig3-figsupp2-data2.zip › Figure 3-Figure supplement 2-source data 2/Figure S6C anti ATP5a; 2024-03-21 13h39m40s.tif]

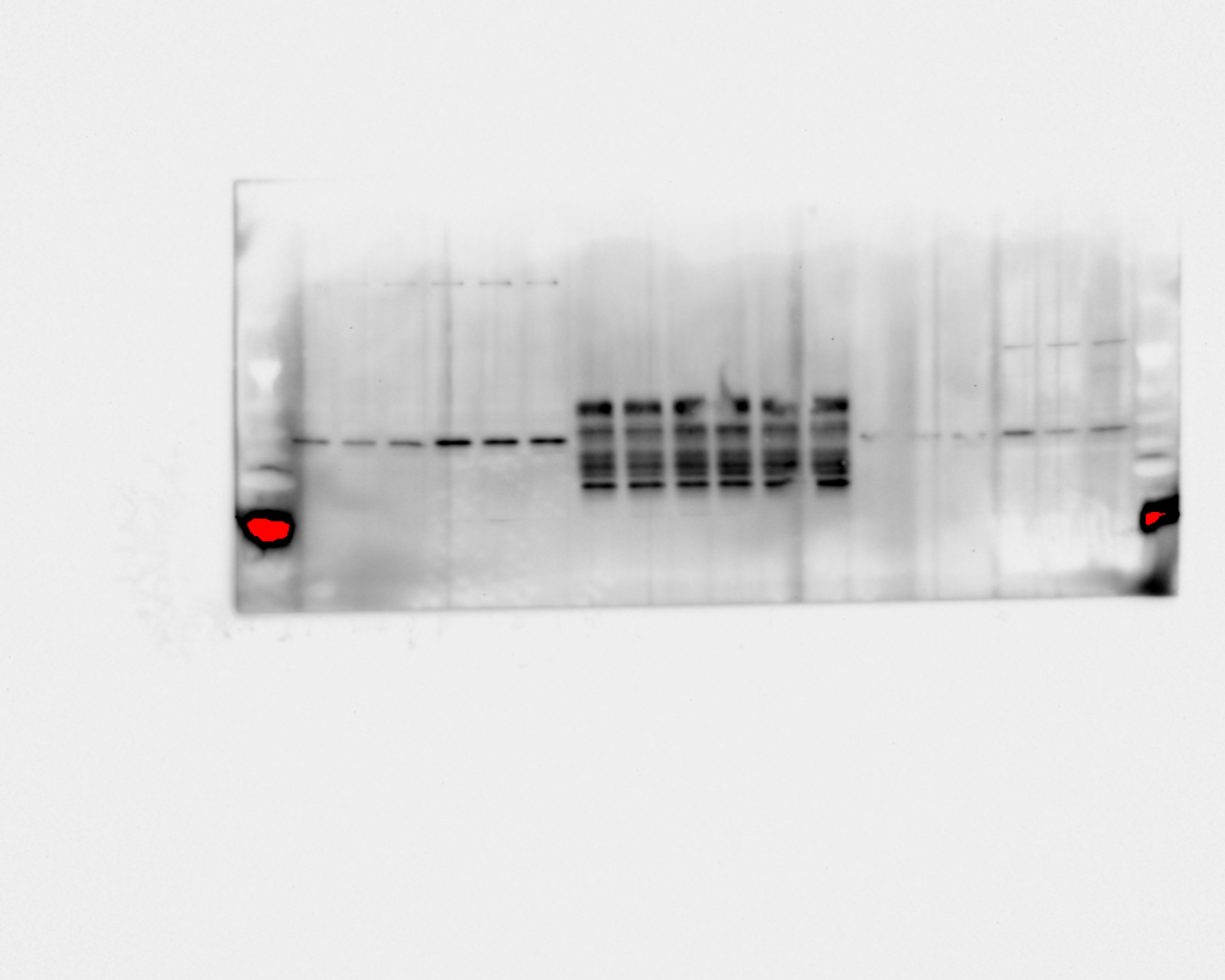

Supplement: Figure 3—figure supplement 2—source data 2. [file elife-99438-fig3-figsupp2-data2.zip › Figure 3-Figure supplement 2-source data 2/Figure S6C anti cypd; 2024-03-15 12h13m36s.tif]

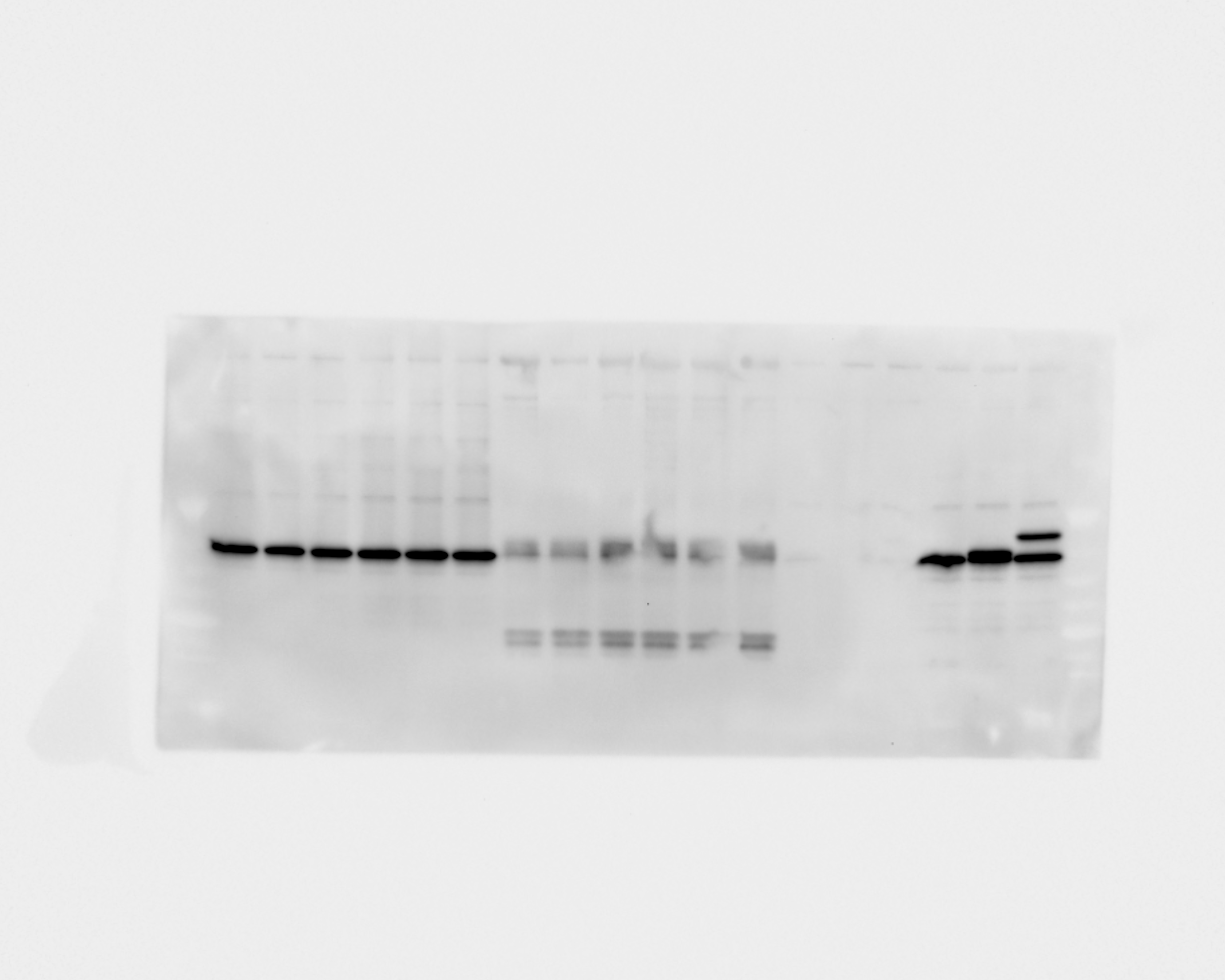

Supplement: Figure 3—figure supplement 2—source data 2. [file elife-99438-fig3-figsupp2-data2.zip › Figure 3-Figure supplement 2-source data 2/Figure S6D anti atp5a; 2024-03-18 13h29m36s.tif]

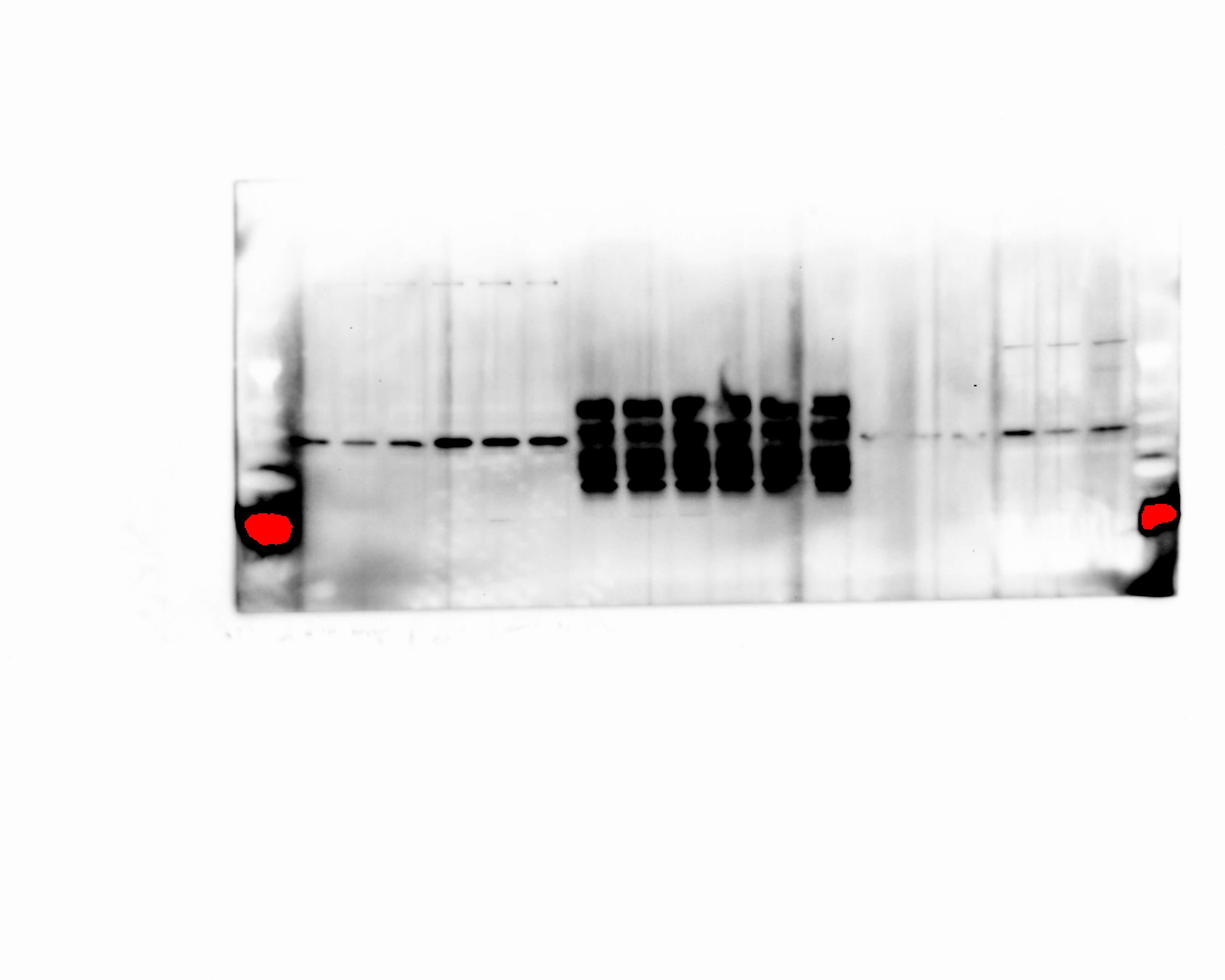

Supplement: Figure 3—figure supplement 2—source data 2. [file elife-99438-fig3-figsupp2-data2.zip › Figure 3-Figure supplement 2-source data 2/Figure S6D anti cypd; 2024-03-15 12h16m04s.tif]

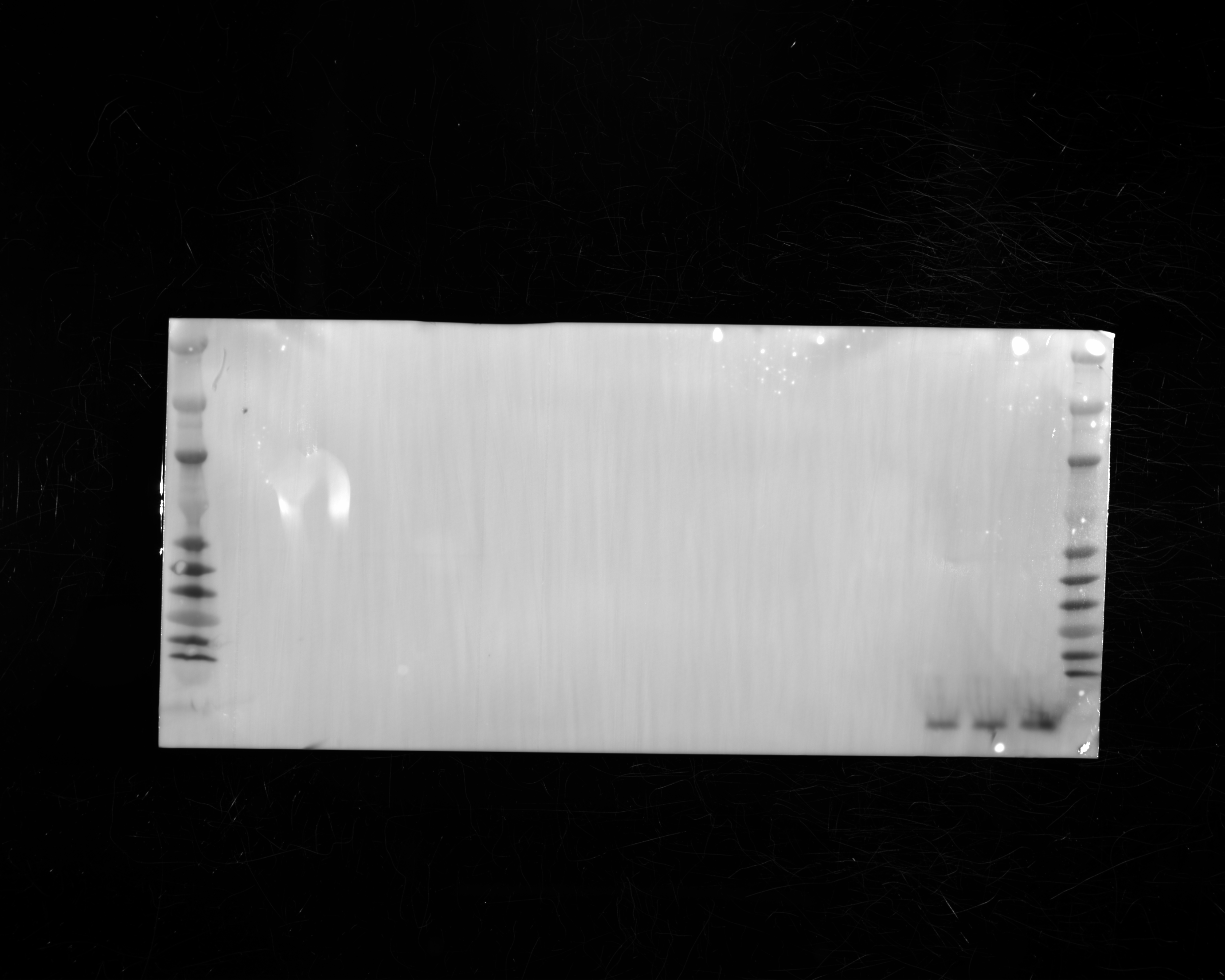

Supplement: Figure 3—figure supplement 2—source data 2. [file elife-99438-fig3-figsupp2-data2.zip › Figure 3-Figure supplement 2-source data 2/Figure S6D anti tom20; 2024-03-18 13h28m47s.tif]

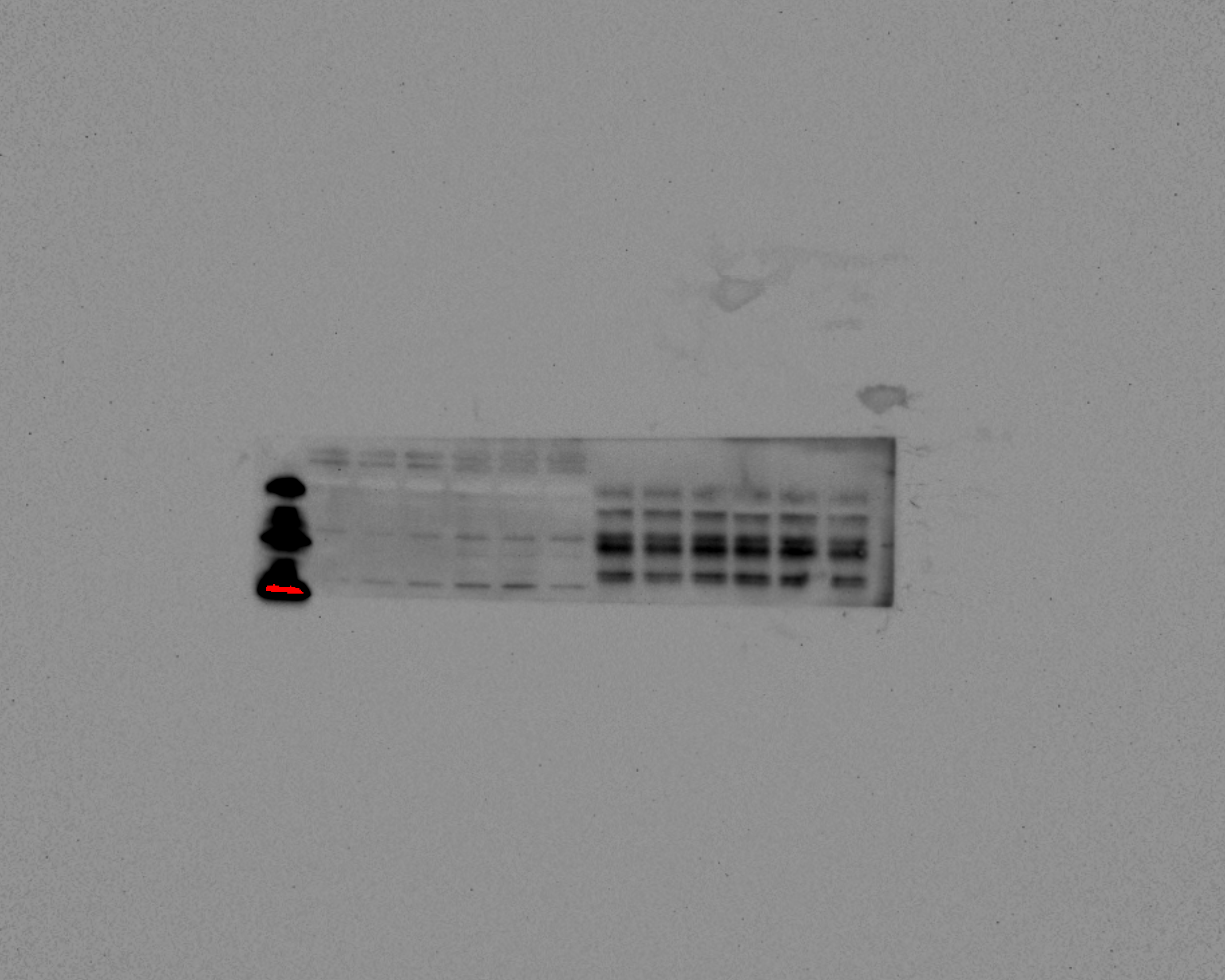

Supplement: Figure 3—figure supplement 2—source data 2. [file elife-99438-fig3-figsupp2-data2.zip › Figure 3-Figure supplement 2-source data 2/Figure S6C anti ant; 2024-04-01 13h13m14s.tif]

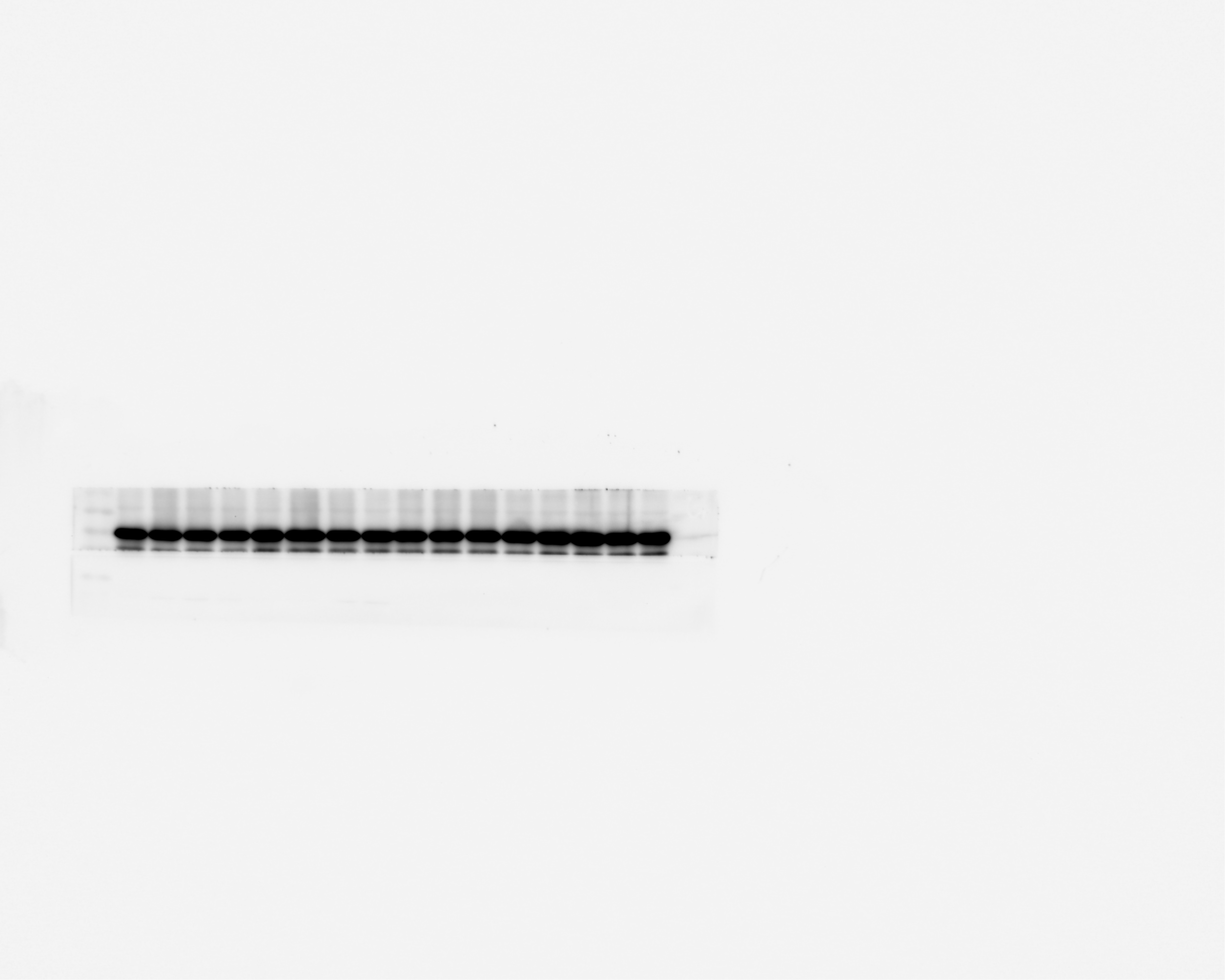

Supplement: Figure 4—figure supplement 2—source data 2. [file elife-99438-fig4-figsupp2-data2.zip › Figure 4-Figure supplement 2-source data 2/Figure S8C anti GAPDH; Ting 2023-04-06 11h34m41s.tif]

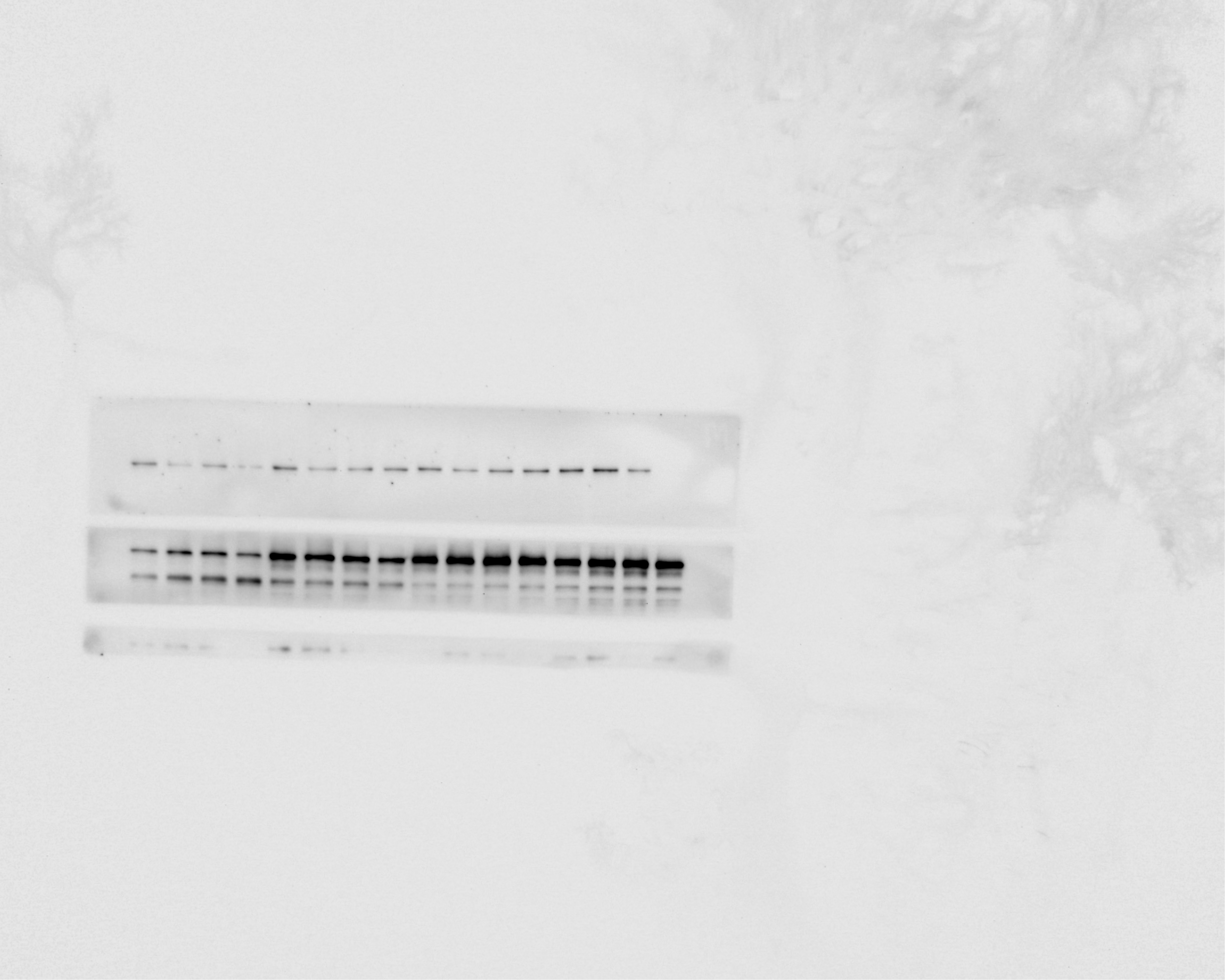

Supplement: Figure 4—figure supplement 2—source data 2. [file elife-99438-fig4-figsupp2-data2.zip › Figure 4-Figure supplement 2-source data 2/Figure S8C anti PARP; Ting 2023-04-13 13h42m59s.tif]

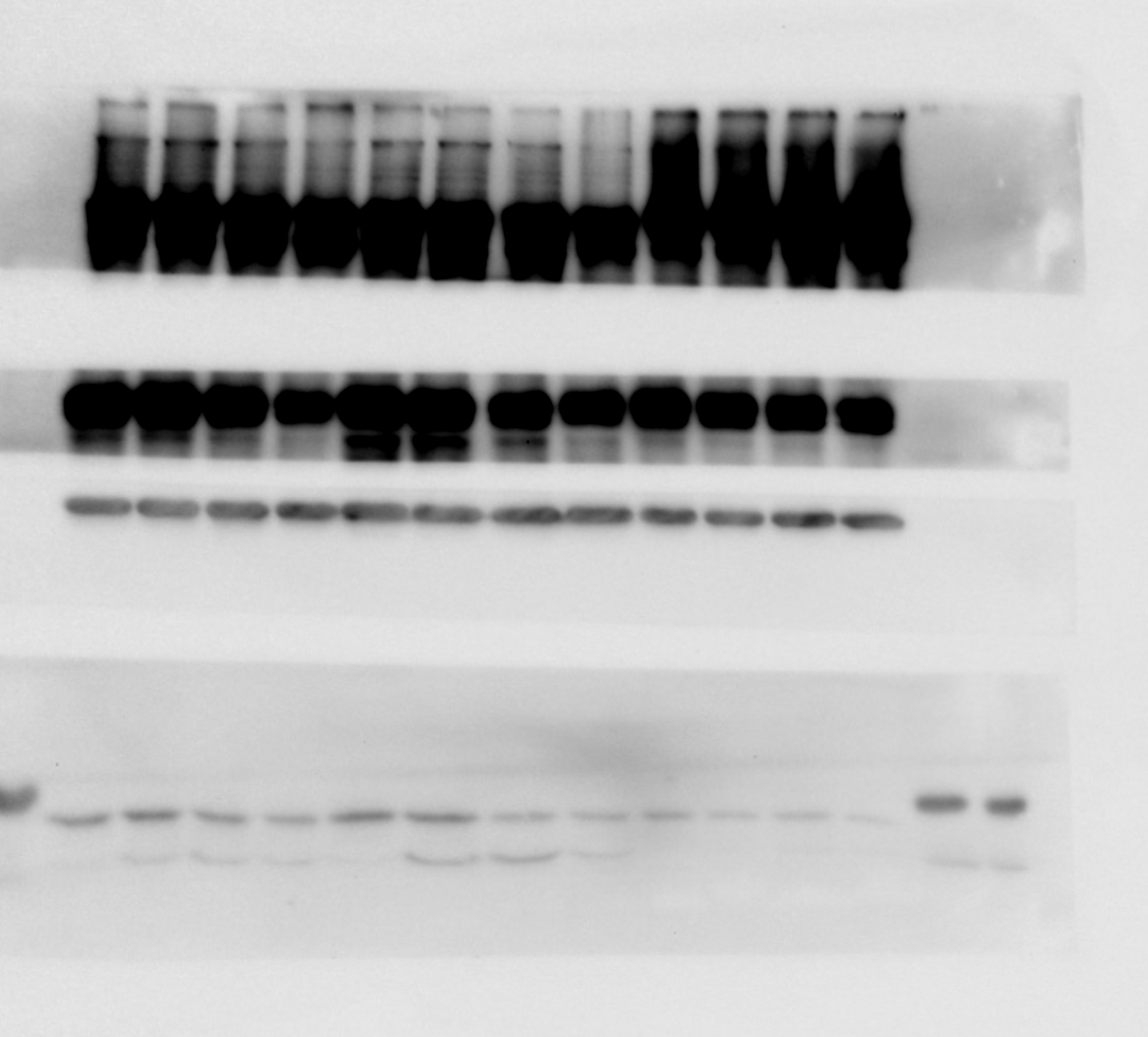

Supplement: Figure 4—figure supplement 2—source data 2. [file elife-99438-fig4-figsupp2-data2.zip › Figure 4-Figure supplement 2-source data 2/Figure S8C anti ACTIN; Ting 2023-07-06 10h01m21s.tif]

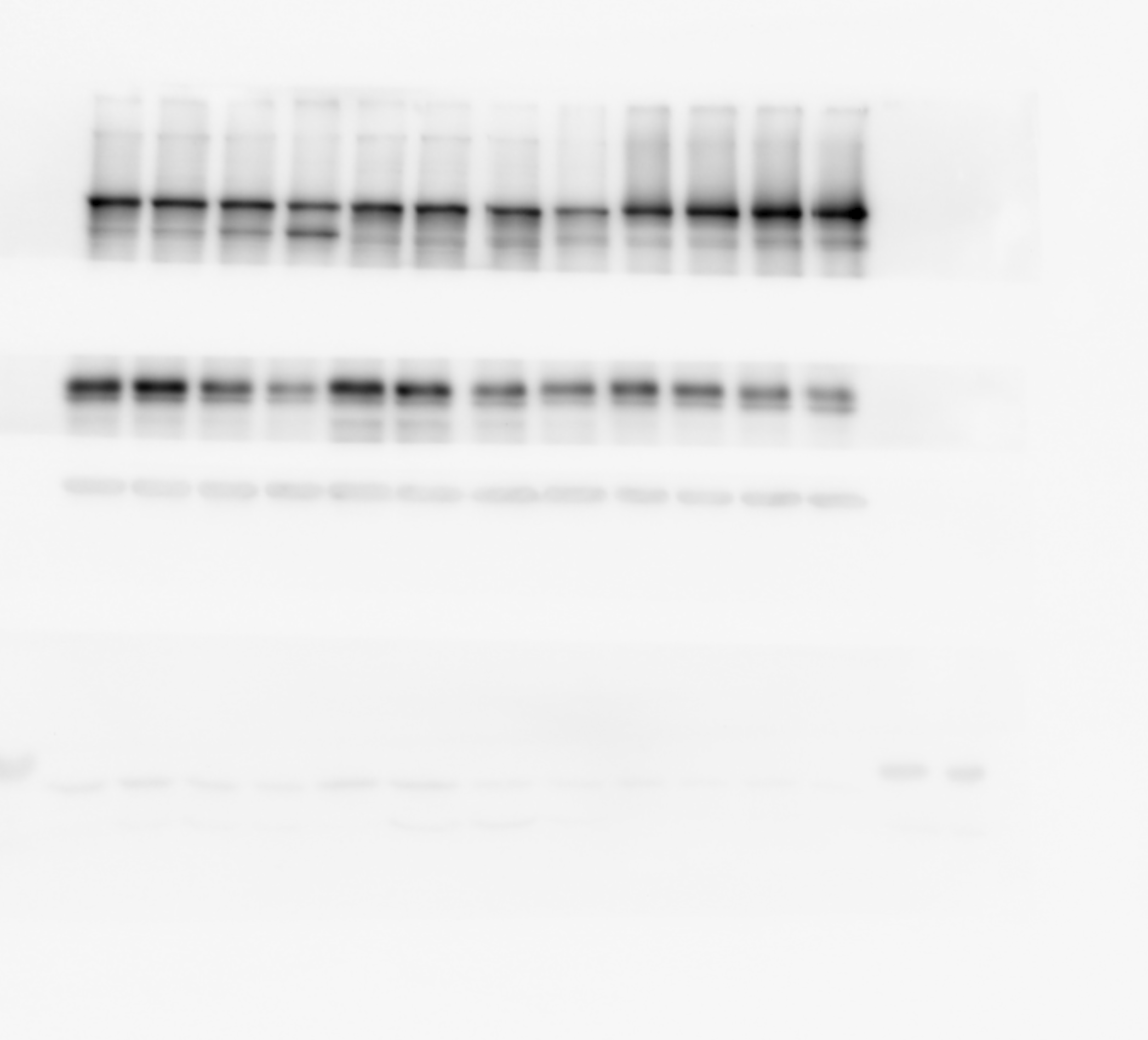

Supplement: Figure 4—figure supplement 2—source data 2. [file elife-99438-fig4-figsupp2-data2.zip › Figure 4-Figure supplement 2-source data 2/Figure S8C anti PARP (2); Ting 2023-07-06 10h00m45s.tif]

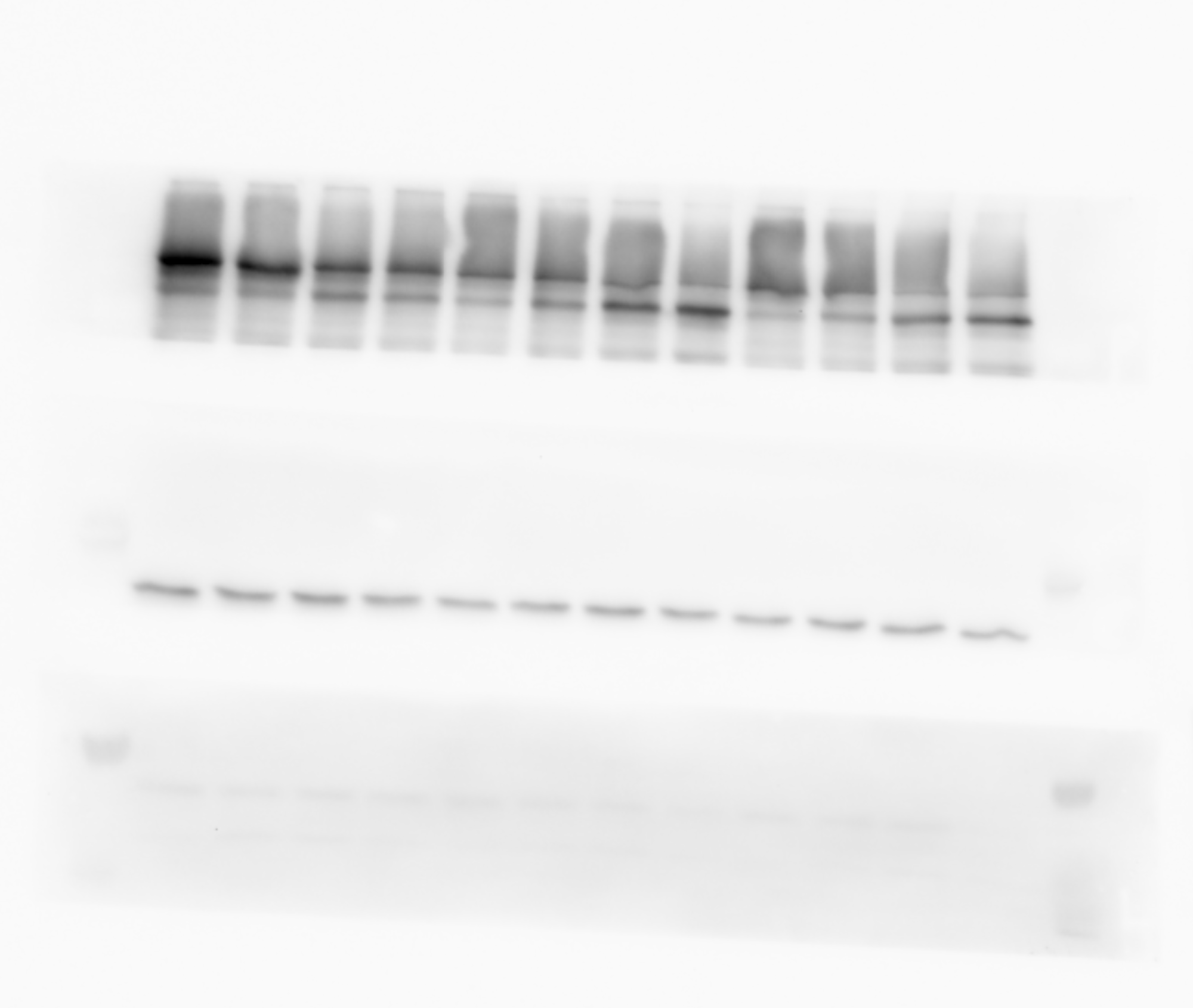

Supplement: Figure 5—figure supplement 1—source data 2. [file elife-99438-fig5-figsupp1-data2.zip › Figure 5-Figure supplement 1-source data 2/Figure S9B aniso anti PARP; Ting 2023-09-27 14h08m34s.tif]

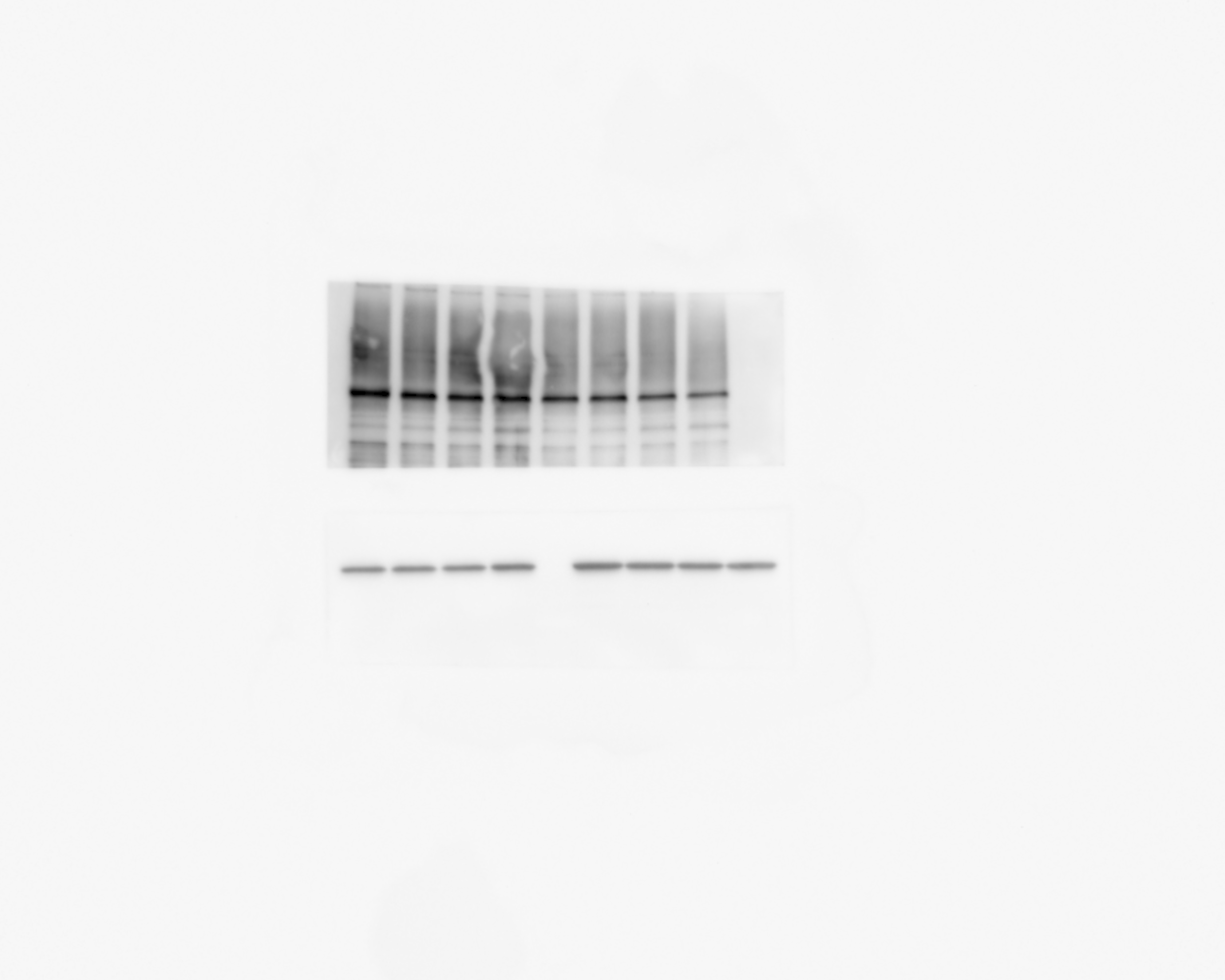

Supplement: Figure 5—figure supplement 1—source data 2. [file elife-99438-fig5-figsupp1-data2.zip › Figure 5-Figure supplement 1-source data 2/Figure S9C cycloheximide anti PARP; 2024-09-19 11h50m04s.tif]

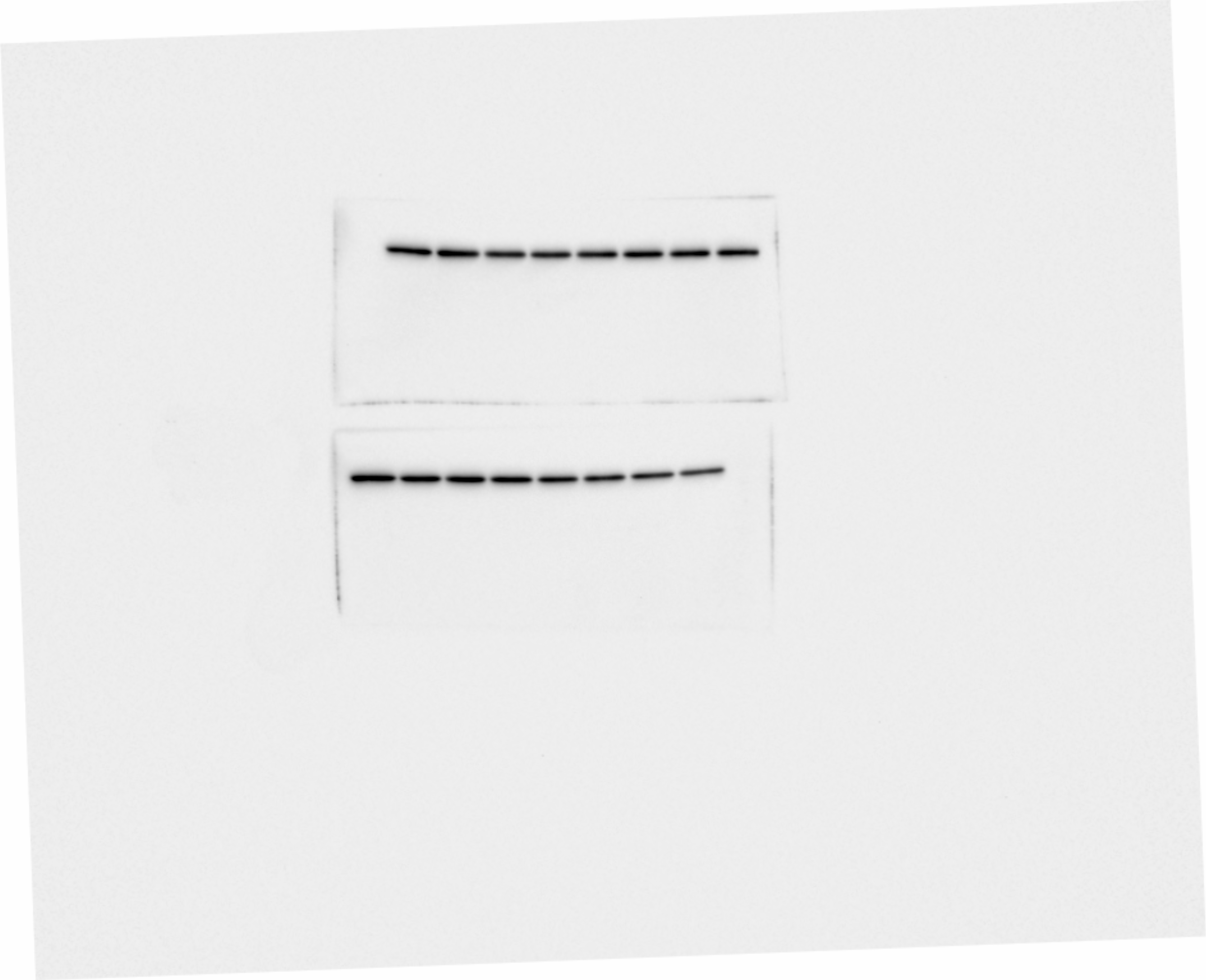

Supplement: Figure 5—figure supplement 1—source data 2. [file elife-99438-fig5-figsupp1-data2.zip › Figure 5-Figure supplement 1-source data 2/Figure S9C cycloheximide anti ACTIN; 2024-09-20 13h21m23s.tif]

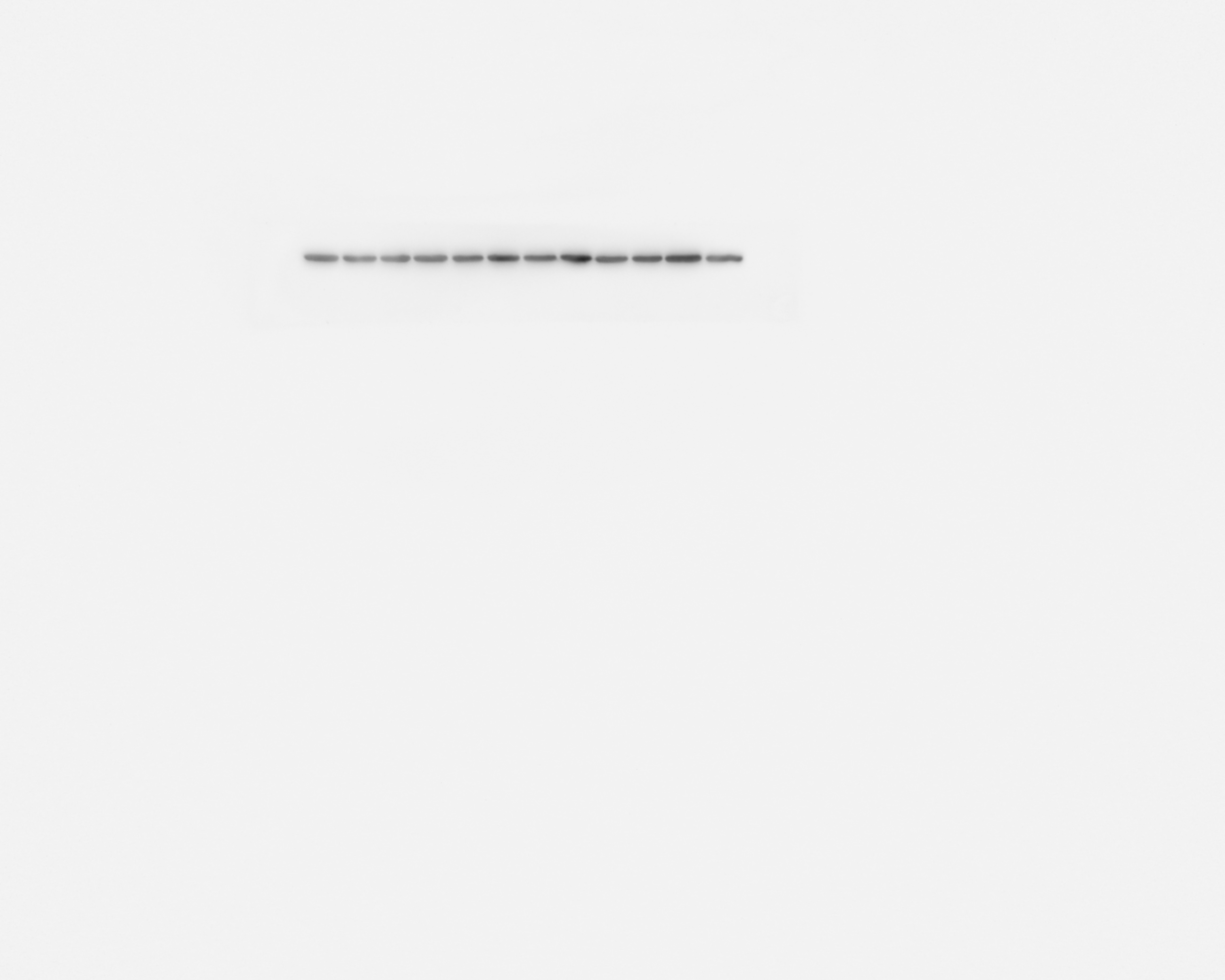

Supplement: Figure 5—figure supplement 1—source data 2. [file elife-99438-fig5-figsupp1-data2.zip › Figure 5-Figure supplement 1-source data 2/Figure S9B aniso anti ACTIN; Ting 2023-09-28 15h37m05s.tif]
