# Supplementary material for: Mitochondrial protein carboxyl-terminal alanine-threonine tailing promotes human glioblastoma growth by regulating mitochondrial function
Source: eLife. 2026 Jan 29;13:RP99438. doi: 10.7554/eLife.99438 (PMC12854676; doi:10.7554/eLife.99438)
Supplement: Figure 1—figure supplement 2—source data 1. [file elife-99438-fig1-figsupp2-data1.zip › Figure 1-Figure supplement 2-source data 1.pdf]

Figure 1 – Figure Supplement 2A

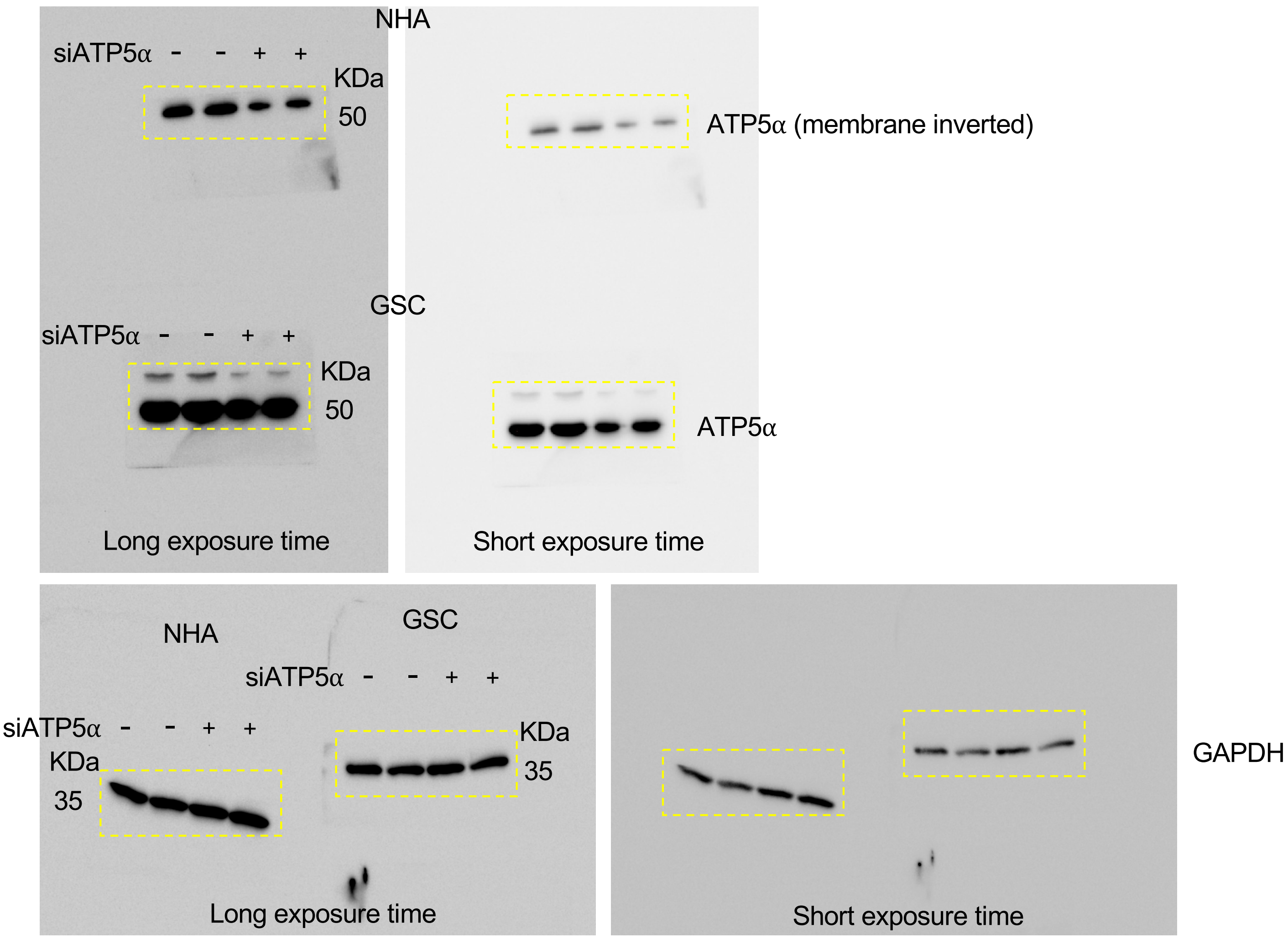

Figure 1-Figure Supplement 2, source data 1
Original membranes corresponding to Figure 1-Figure Supplement 2A.

Figure 1 – Figure Supplement 2B

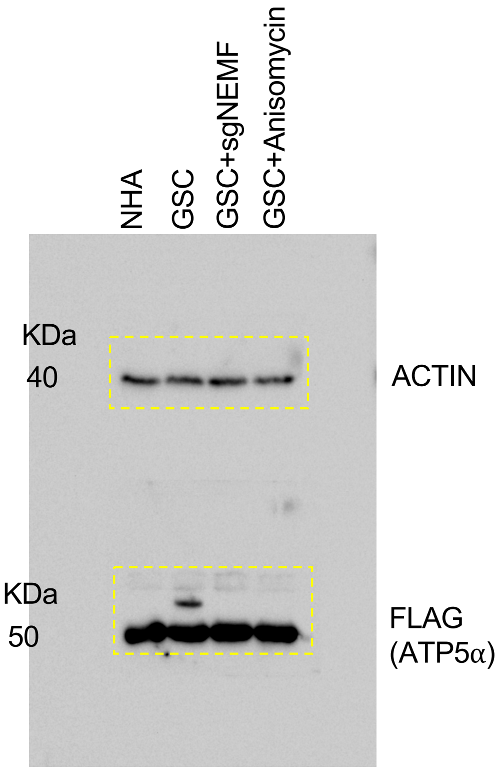

Figure 1-Figure Supplement 2, source data 1
Original membranes corresponding to Figure 1-Figure Supplement 2B.

Figure 1 – Figure Supplement 2E

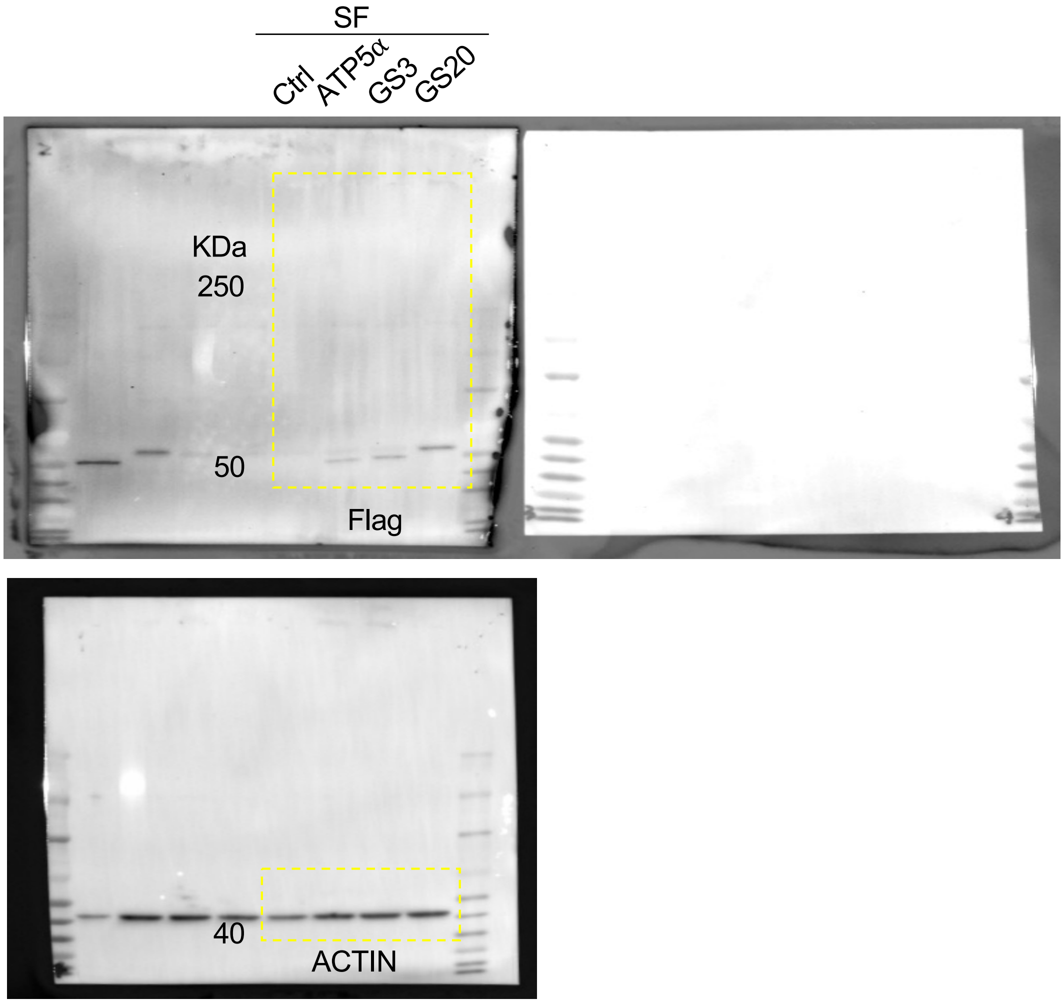

Figure 1-Figure Supplement 2, source data 1

Original membranes corresponding to Figure 1-Figure Supplement 2E.
