# Supplementary material for: Mitochondrial protein carboxyl-terminal alanine-threonine tailing promotes human glioblastoma growth by regulating mitochondrial function
Source: eLife. 2026 Jan 29;13:RP99438. doi: 10.7554/eLife.99438 (PMC12854676; doi:10.7554/eLife.99438)
Supplement: Figure 3—figure supplement 2—source data 1. [file elife-99438-fig3-figsupp2-data1.zip › Figure 3-Figure supplement 2-source data 1.pdf]

Figure 3 – Figure Supplement 2C

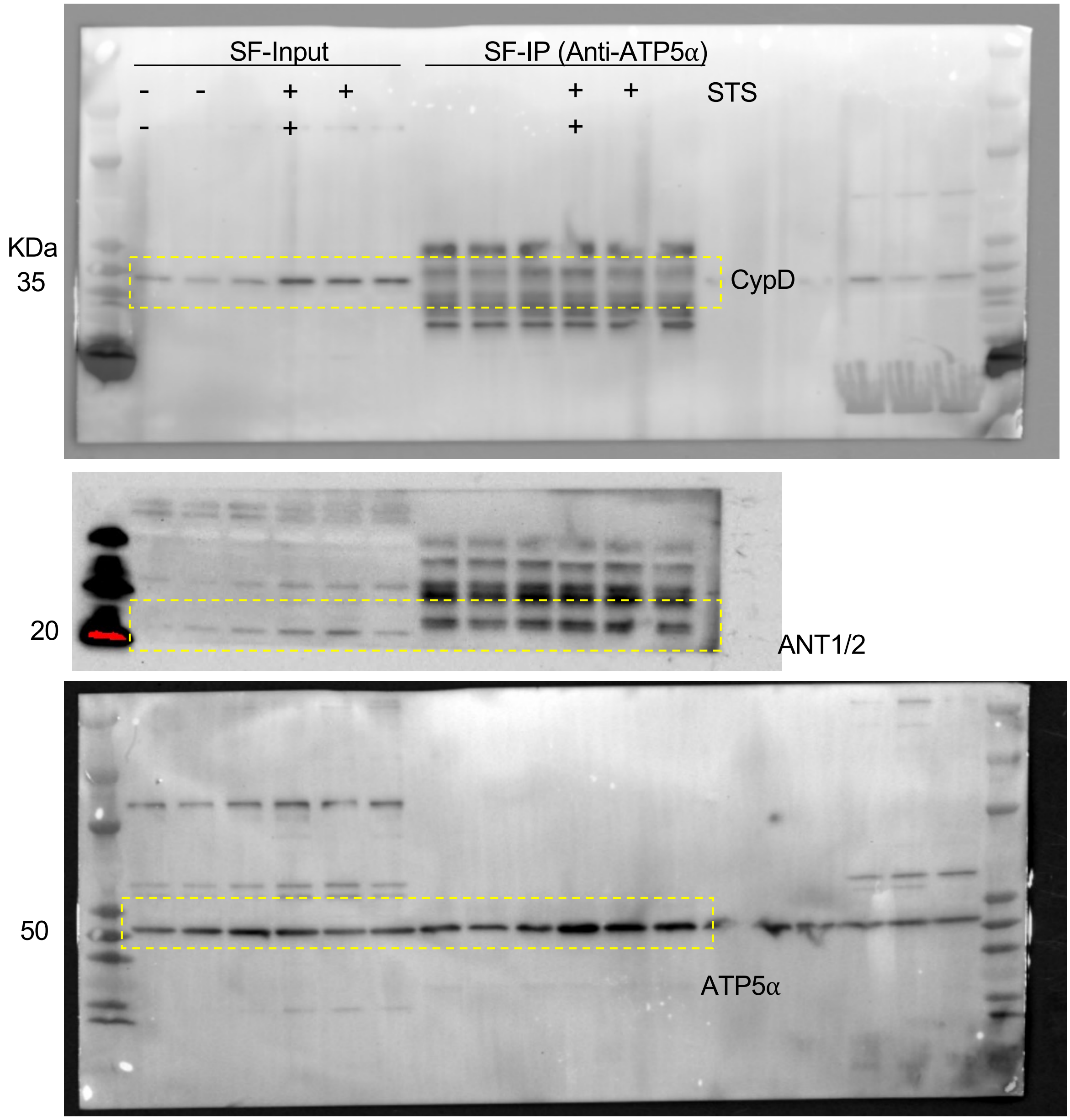

Figure 3-Figure Supplement 2, source data 1

Original membranes corresponding to Figure 3-Figure Supplement 2C.

Figure 3 – Figure Supplement 2D

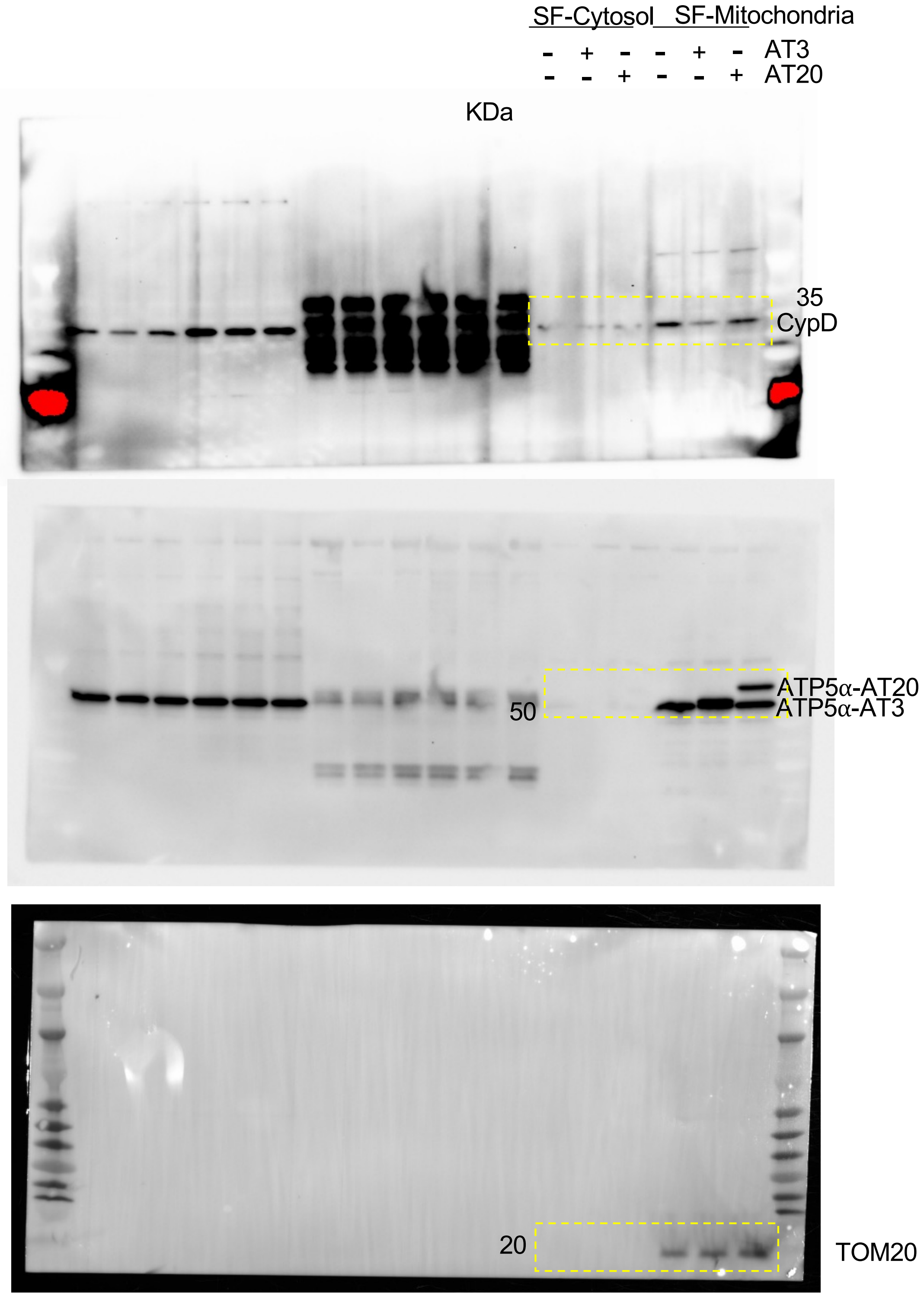

Figure 3-Figure Supplement 2, source data 1
Original membranes corresponding to Figure 3-Figure Supplement 2D.
