# Supplementary material for: Mitochondrial protein carboxyl-terminal alanine-threonine tailing promotes human glioblastoma growth by regulating mitochondrial function
Source: eLife. 2026 Jan 29;13:RP99438. doi: 10.7554/eLife.99438 (PMC12854676; doi:10.7554/eLife.99438)
Supplement: Figure 5—figure supplement 1—source data 1. [file elife-99438-fig5-figsupp1-data1.zip › Figure 5-Figure supplement 1-source data 1.pdf]

Figure 5 – Figure Supplement 1B

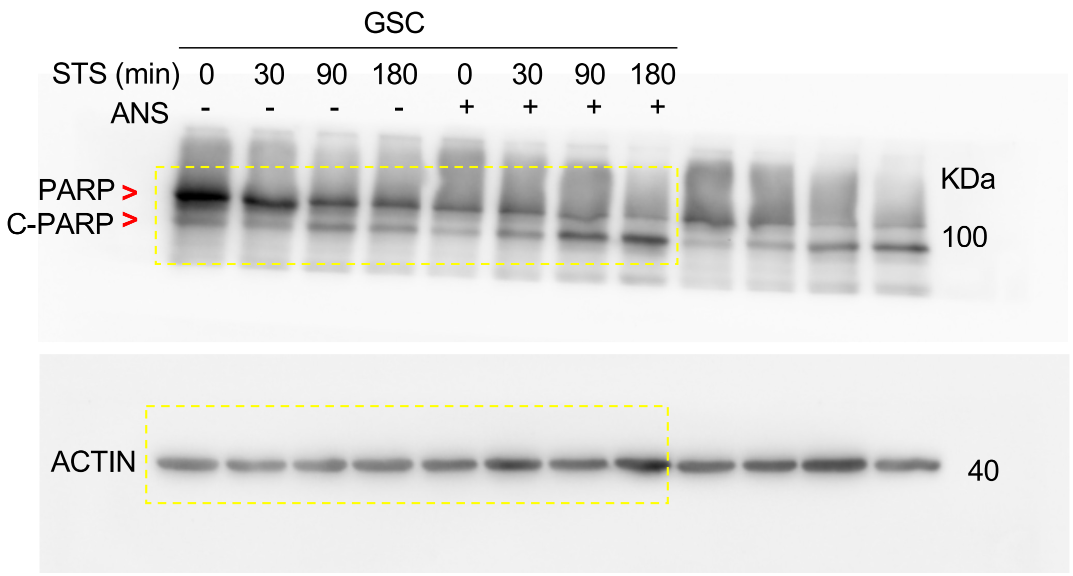

Figure 5-Figure Supplement 1, source data 1
Original membranes corresponding to Figure 5-Figure Supplement 1B.

Figure 5 – Figure Supplement 1C

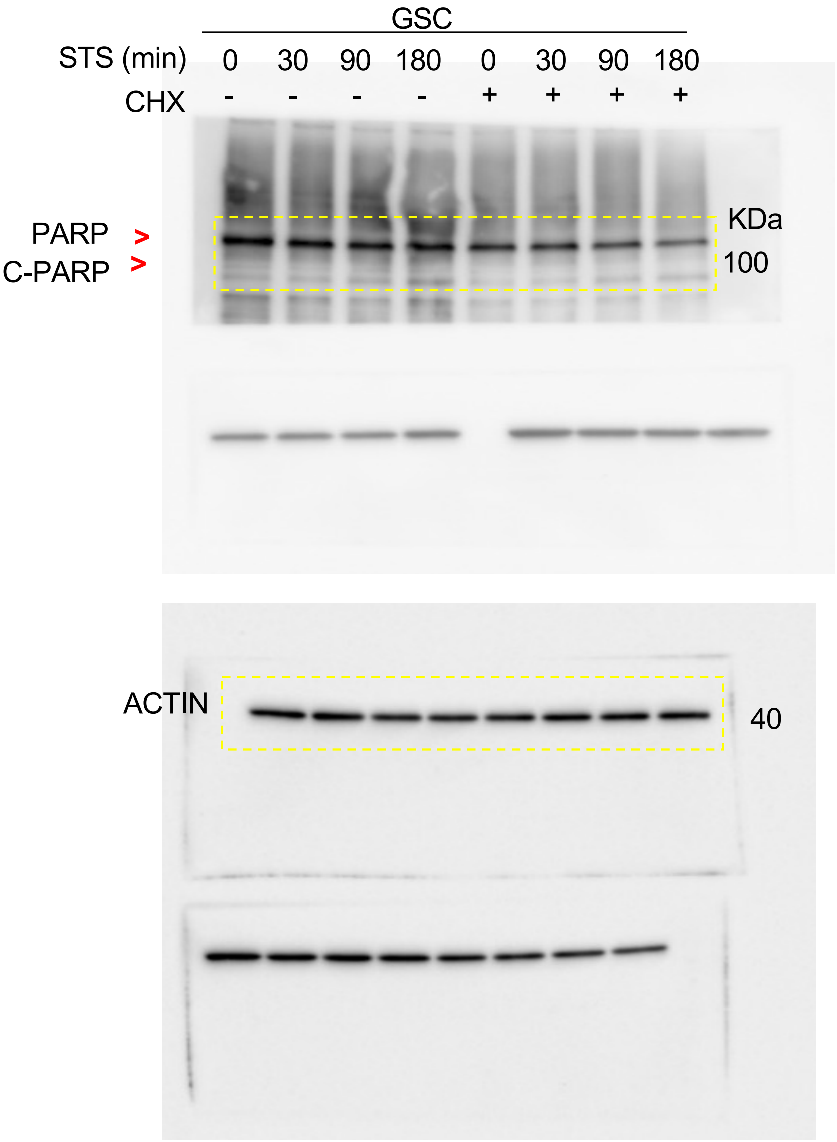

Figure 5-Figure Supplement 1, source data 1
Original membranes corresponding to Figure 5-Figure Supplement 1C.
